# Phylogeny, biogeography and diversification patterns of side-necked turtles (Testudines: Pleurodira)

**DOI:** 10.1098/rsos.171773

**Published:** 2018-03-28

**Authors:** Gabriel S. Ferreira, Mario Bronzati, Max C. Langer, Juliana Sterli

**Affiliations:** 1 Biology Department, FFCLRP, University of São Paulo, Ribeirão Preto, Brazil; 2 Senckenberg Center for Human Evolution and Palaeoenvironment (HEP) at Eberhard Karls Universität, Sigwartstraße 10, 72076 Tübingen, Germany; 3 Fachbereich Geowissenschaften der Eberhard Karls Universität Tübingen, Hölderlinstraße 12, 72074 Tübingen, Germany; 4 Bayerische Staatssammlung für Paläontologie und Geologie, Richard-Wagner-Strasse 10, 80333 Munich, Germany; 5 Department of Earth and Enviromental Sciences, Ludwig–Maximilians–Universität, Richard-Wagner-Strasse 10, 80333 Munich, Germany; 6 CONICET-Museo Paleontológico Egidio Feruglio, Fontana 140, 9100 Trelew, Chubut, Argentina

**Keywords:** Pleurodira, historical biogeography, BioGeoBEARS, transoceanic dispersal, diversity

## Abstract

Pleurodires or side-necked turtles are today restricted to freshwater environments of South America, Africa–Madagascar and Australia, but in the past they were distributed much more broadly, being found also on Eurasia, India and North America, and marine environments. Two hypotheses were proposed to explain this distribution; in the first, vicariance would have shaped the current geographical distribution and, in the second, extinctions constrained a previously widespread distribution. Here, we aim to reconstruct pleurodiran biogeographic history and diversification patterns based on a new phylogenetic hypothesis recovered from the analysis of the largest morphological dataset yet compiled for the lineage, testing which biogeographical process prevailed during its evolutionary history. The resulting topology generally agrees with previous hypotheses of the group and shows that most diversification shifts were related to the exploration of new niches, e.g. littoral or marine radiations. In addition, as other turtles, pleurodires do not seem to have been much affected by either the Cretaceous–Palaeogene or the Eocene–Oligocene mass extinctions. The biogeographic analyses highlight the predominance of both anagenetic and cladogenetic dispersal events and support the importance of transoceanic dispersals as a more common driver of area changes than previously thought, agreeing with previous studies with other non-turtle lineages.

## 1. Introduction

The turtle crown group Testudines is composed of two lineages with extant taxa [1]: cryptodires or hidden-necked turtles, and pleurodires or side-necked turtles. Although early studies (e.g. [2,3]) proposed a Triassic origin for the crown group, more recent analyses suggest a maximum age of *ca* 165 Ma (Middle Jurassic) [4,5]. Stem-pleurodires are known from Late Jurassic and Early Cretaceous deposits [6], but the oldest records of the crown group come from the Barremian (Early Cretaceous) of Brazil [7,8].

Pleurodires today represent a small fraction of the diversity of Testudines (93 of 356 species; [9]) and are restricted to freshwater environments (although some chelids seem to tolerate higher levels of salinity; [10,11]) of Africa, Australia, Madagascar and South America [12]. Their fossil record, however, reveals a much broader distribution, including Eurasia, India and North America (e.g. [12–14]), as well as taxa adapted to marine (at least coastal) environments (e.g. [12,15,16]). The crown group includes three lineages with extant representatives, Chelidae, Pelomedusidae and Podocnemididae, and three extinct groups, Araripemydidae, Euraxemyidade and Bothremydidae [12,13,17]. Podocnemididae and Bothremydidae are by far the most abundant pleurodires in the fossil record, and a proposed peak in pleurodire diversity during the Cretaceous and Paleocene seems to be related to the diversification of those two groups [12].

The biogeographic distribution of extant pleurodires, which are restricted to some areas of the Southern Hemisphere, has been the subject of many investigations. A classical viewpoint (e.g. [18,19]) is that the disjunct Pelomedusoides (Pelomedusidae + Podocnemididae; see the electronic supplementary material for definitions of pleurodiran clades) distribution represents the outcome of vicariant events, in this case caused by the break-up of the supercontinent Gondwana. Contrary to those interpretations, Noonan [20], using a phylogenetic hypothesis based on molecular data combined with that of Meylan [21] from fossil taxa, suggested that the distribution of extant pelomedusoids is a remnant of a much more widespread pattern, shaped by large-scale extinctions. Later, Romano & Azevedo [22], based on a reanalysis of the morphological data matrix of de la Fuente [23], adding more fossil taxa, corroborated the idea of vicariant events shaping the biogeography of pelomedusoids. As for chelids, even though molecular (e.g. [5,24–28]) and morphological (e.g. [17,29,30]) results disagree regarding the position of the Australian and South American taxa, both hypotheses return similar biogeographic interpretations, where the group starts to diversify prior to the separation of Australia from the remaining of Gondwana, suggesting a widespread distribution for the group before this vicariant event [31,32].

The above-mentioned studies were, however, conducted prior to several fossil findings that greatly increased our knowledge of pleurodiran taxonomic, morphological and distributional diversities (e.g. [8,14,16]). Additionally, although some large phylogenetic analyses have been conducted (e.g. [12–14]), sampling a variety of fossil taxa, these were restricted to some pleurodiran subclades, and no phylogenetic analysis including a comprehensive sample of all lineages of the group has been, to our knowledge, so far conducted. Considering that reliable phylogenetic frameworks are necessary to conduct numerical diversity and biogeographic analyses [33–35], a well-sampled phylogenetic hypothesis for Pleurodira is needed to test the previously proposed evolutionary scenarios.

To fulfil the above-mentioned requirement, we compiled the largest morphological matrix including a broad taxon sample of all pleurodiran lineages, obtaining, to our knowledge, the most inclusive phylogenetic hypothesis so far proposed for the clade. Based on this new hypothesis, we conducted diversification and historical biogeography analyses to explore the evolution of the side-necked turtles.

## 2. Material and methods

### 2.1. Phylogenetic analyses and time-scaled trees

A new taxon–character matrix (101 taxa × 245 characters) for Pleurodira was built using Mesquite v. 3.0 [36]. The character list is largely based on the extensive studies of Gaffney *et al*. [12,13], with additions from other sources (e.g. [14,23,37–42]), especially those focusing on chelids [17,29,43], and 18 new characters proposed here. Special effort was made to better sample post-cranial structures, resulting in 97 characters from that partition (39.5% of the total), more than in any previous study (e.g. [12] and [14] had 29.7% and 36.4% of post-cranial characters, respectively). Twelve characters were interpreted as forming morphoclines and ordered accordingly (see the electronic supplementary material for additional information about the phylogenetic analysis).

The taxon sample was conceived to incorporate all pleurodiran lineages, namely Chelidae, Pelomedusidae, Araripemydidae, Euraxemydidae, Bothremydidae and Podocnemididae, including 98 crown pleurodires as terminal taxa (see the electronic supplementary material for the complete taxon list). Previously, the largest matrices for pleurodires comprised 43 [8] and 91 [14] in-group taxa (although the latter study employed a reduced version, with 70 in-group taxa, in the main analyses). The non-Testudines Testudinata *Proganochelys quenstedti* and the stem-pleurodires *Notoemys laticentralis* and *Platychelys oberndorferi* composed the outgroup taxa.

The resulting matrix was analysed under the parsimony criterion in TNT v. 1.1 [44] via a heuristic search with the following settings: 2000 replicates of Wagner trees, random seed = 0, tree bisection reconnection (TBR) for branch-swapping, hold = 20 and collapse of zero-length branches according to rule ‘1' of TNT. The most parsimonious trees (MPTs) found in this first round of the analysis were the subject of a second round of TBR. A strict consensus tree, decay (Bremer support) and resampling (bootstrap and jackknife) values were obtained using implemented functions on TNT. The resampling values were calculated using 1000 replicates for absolute and difference of frequencies (group present/contradicted or GC in [45]). Consistency indexes (CI) and retention indexes (RI) were calculated using the script *statsall* (designed by Peterson L. Lopes, v. 1.3). The IterPCR script [46] was used to identify unstable taxa during preliminary analyses (i.e. taxa with multiple alternative positions) and to re-evaluate our scoring when the instability was caused by conflict of information rather than missing data. Additionally, considering that molecular-based phylogenetic analyses retrieve distinct arrangements for several extant taxa (e.g. [20,24,25,27]), we ran a second analysis (referred to hereafter as the ‘molecular constrained' analysis), following the same settings as the previous (referred to hereafter as the ‘original' analysis), except for setting constraints (see the electronic supplementary material) for the relations of the extant taxa based on the molecular phylogenetic hypothesis of Guillon *et al*. [25]. We also conducted three additional constrained analyses to evaluate how many steps were needed to achieve arrangements found in other alternative hypotheses.

In addition to the ‘original' and ‘molecular constrained' trees, we obtained two additional topologies to be employed in the subsequent analyses. The ‘non-marine taxa tree' was built for the biogeography analyses by pruning taxa previously considered marine or adapted to brackish water, i.e. Bothremydini & Stereogenyini [12,15,16,47] from the strict consensus tree of the ‘original' analysis. Further, an informal ‘supertree' was built for the diversity and diversification analyses by adding extinct and extant taxa not included in the ‘original' phylogenetic analysis to the strict consensus tree. The four topologies were time-scaled with the R [48] package *strap* [49], using information from the literature to define time ranges for each taxon (see the electronic supplementary material) and dividing branch lengths equally along the tree to avoid zero or close-to-zero values [50]. As the biogeographic analysis requires fully dichotomous topologies, the polytomies of the ‘original’ tree were manually resolved by deliberately choosing particular arrangements (see the electronic supplementary material for additional topologies).

### 2.2. Diversification analysis and diversity curves

The diversification analyses were conducted on the software SymmeTree [51] using time-sliced trees, according to the procedure presented by Tarver & Donoghue [52] (but see [53] for an alternative approach). Accordingly, each time-sliced tree is composed of a subset of the original tree containing taxa of the same age or older than the given period, in addition to the ghost lineages of younger taxa. Seven different intervals were created: Early Cretaceous, Late Cretaceous, Paleocene, Eocene, Oligocene, Miocene and Pliocene-Recent. Still, we followed the procedure outlined in Bronzati *et al*. [54] when a diversification shift was detected in a time interval younger than the clade it is referred to, in order to differentiate real shifts (i.e. detected shifts caused by diversification) from artefacts (i.e. detected shifts resulting from speciation and extinction). We conducted two analyses, one with the ‘original' tree and a second with the ‘supertree', in order to evaluate the effect of the sampling of our matrix in the resulting shifts.

We also built two types of diversity curves across geological time for natural (i.e. clades) and artificial (i.e. ecological guilds) groups of Pleurodira, to compare the diversity variation with biogeographic and climatic events. In this procedure, we used the ‘supertree' to increase our sample. ‘Taxic diversity curves' were obtained using the total number of taxa per time bin. Considering that this kind of data is much affected by the incompleteness of the fossil record and the number of fossiliferous deposits on a given period, a second type of curve was created for comparison, called ‘phyletic diversity curve', that also takes into account the number of ghost lineages in each of the intervals.

### 2.3. Estimation of ancestral ranges and number and types of biogeographical events

We conducted a series of ancestral area reconstructions on the R package BioGeoBears, which implements three of the most used models in historical biogeography analyses in a common likelihood framework [55], namely the LAGRANGE dispersal–extinction–cladogenesis (DEC) model [34,56], a likelihood version of DIVA (DIVALIKE) [57] and a likelihood version of the range evolution model (BAYAREA) implicit to the methods BayArea [58] and Bayesian binary model (BBM) of RASP [59]. Each of those methods makes different assumptions about anagenetic and cladogenetic range change processes [35], and those assumptions usually have a large impact on the results [60]. The multi-model approach implemented in BioGeoBears allows the evaluation of competing hypotheses potentially generated by those models by comparing the fit of their assumptions to the observed data [55,60]. To compare and choose between the different models, we conducted a likelihood ratio test (LRT) for the nested models and the Akaike information criterion corrected for sample size (AICc) for the non-nested models.

We ran a first set of time-stratified analyses using a time-scaled version of the ‘original' tree, accounting for nine models: the standard models (herein named M_0_) DEC, DIVALIKE and BAYAREA [55], and two additional versions to each of those including *x* [61] and *j* [60] as free parameters (herein named M_1_ and M_2_, respectively). The *x* parameter is used to estimate the relative probability of dispersal as a function of distance, modifying the dispersal rates from area *A* to area *B* by (distance [*A* to *B*])*^x^* [61]. The distances between the areas are established by user-defined matrices (see the electronic supplementary material). When *x* is set as a free parameter, the distance between the areas may influence each branch on the tree differently. With *j* as a free parameter, founder-event speciation is added to the model, allowing that—during cladogenesis—one of the descendants jumps to a new range outside that of the ancestor without prior range expansion [60].

Considering that marine taxa are not affected by the oceanic barriers that separate continental areas as terrestrial or freshwater turtles are, distances may influence differently their dispersal capabilities, and jumps to new areas without prior range expansion may not be uncommon. This justifies testing the influence of *x* and *j* in additional models [55,62]. As marine taxa are usually excluded from biogeographic inferences (e.g. [63]), we ran a second set of stratified analyses using the ‘non-marine taxa' tree for comparison. Moreover, phylogenetic hypotheses for pleurodires using molecular data usually result in different arrangements for Australian/South American chelids and South American/Malagasy podocnemidids (e.g. [5,20,25,27]). To test if those different topologies imply distinct ancestral area reconstructions, we ran a third set of stratified analyses using the ‘molecular constrained' tree. The two latter sets of analyses were conducted using only the nested models (M_0_, M_1_ and M_2_) of DEC and DIVALIKE, because BAYAREA performed worse than those for the first set (see Results).

Three time ranges with their respective distance matrices were considered as they are thought to represent intervals with a similar configuration of landmasses: 170–91.1, 91.1–55.5 and 55.5–0 Ma. We considered 10 possible areas: South America, Africa, North America, Madagascar, Australia, Europe, India, Arabia/Middle East, East Asia and Antarctica. Even though there is no record of pleurodires in Antarctica, it could act as a land bridge between closely related areas in some time bins and, as such, could appear as a reconstructed ancestral area. Three distance matrices between the areas were defined, one for each time slice.

We also conducted biogeographical stochastic mapping (BSM) implemented in BioGeoBears [64] on the ‘original' tree dataset, using M_1_ and M_2_ models of DIVALIKE and DEC, to evaluate the impact of a given model on the estimated frequencies of each type of range change event. BSM simulates possible biogeographic histories constrained to produce the observed data of a given dataset, estimating the times and positions of all events on the tree [65]. Means and standard deviations of event counts from 50 BSM were used to estimate event frequencies.

## 3. Results

### 3.1. Phylogenetic analyses

**Figure 1.**
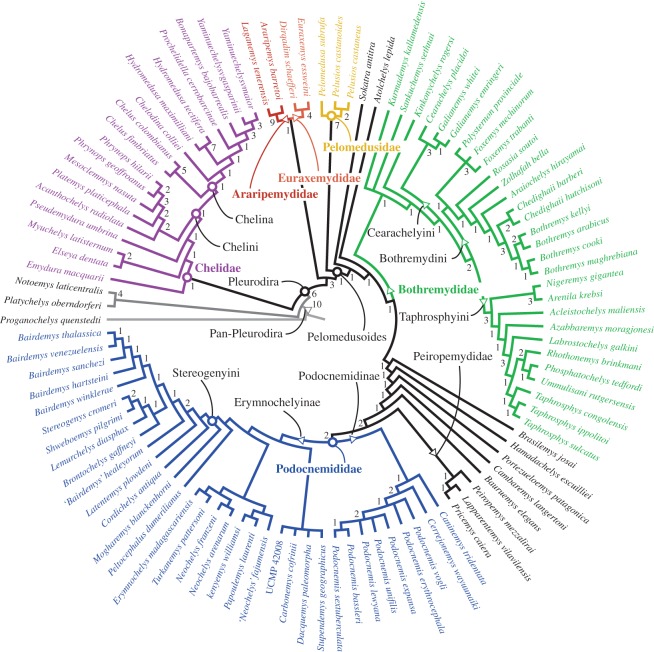
Strict consensus of 36 MPTs of 1128 steps each (CI = 0.290, RI = 0.750). Numbers below nodes represent Bremer support values higher than 0. Names refer to node- (circles) and branch-based (triangles) clades.

Thirty-six MPTs of 1128 steps (CI = 0.290, RI = 0.750) were found by the unconstrained analysis of the ‘original' matrix (best score hit 388 times). Bremer support (BS), bootstrap and jackknife values, lists of characters and common synapomorphies, as well as phylogenetic definitions for the clades and a more detailed description of the results are provided in the electronic supplementary material. The strict consensus tree (figure 1) is well resolved (93% of the possible nodes) and mostly agrees with previous morphology-based analyses, recovering the monophyly of the main pleurodiran lineages with high support. The ‘constrained' analysis found 756 MPTs of 1175 steps. Although much longer, the resulting strict consensus tree is very similar to the ‘original' (see the electronic supplementary material), with the exception of the forced relations between extant taxa.

### 3.2. Diversification shifts and diversity levels

**Figure 2.**
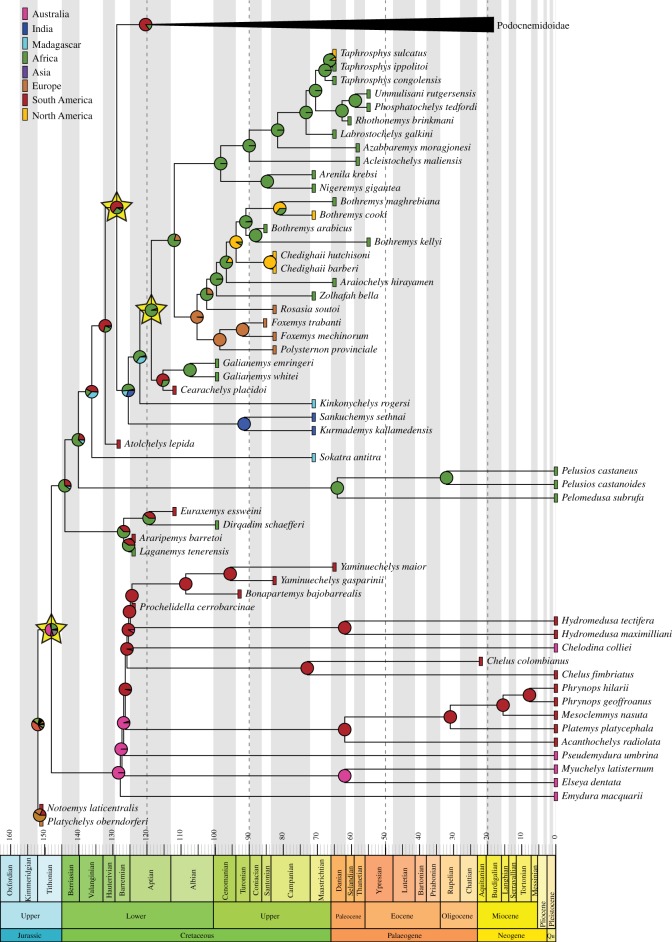
Ancestral area reconstructions and diversification shifts for the time-calibrated ‘original' tree excluding Podocnemidoidae (figure 3). Rectangles next to each terminal taxon represent its area distribution, pie charts represent the probabilities for ancestral area of nodes and yellow stars point to a node in which a diversification shift was found.

**Figure 3.**
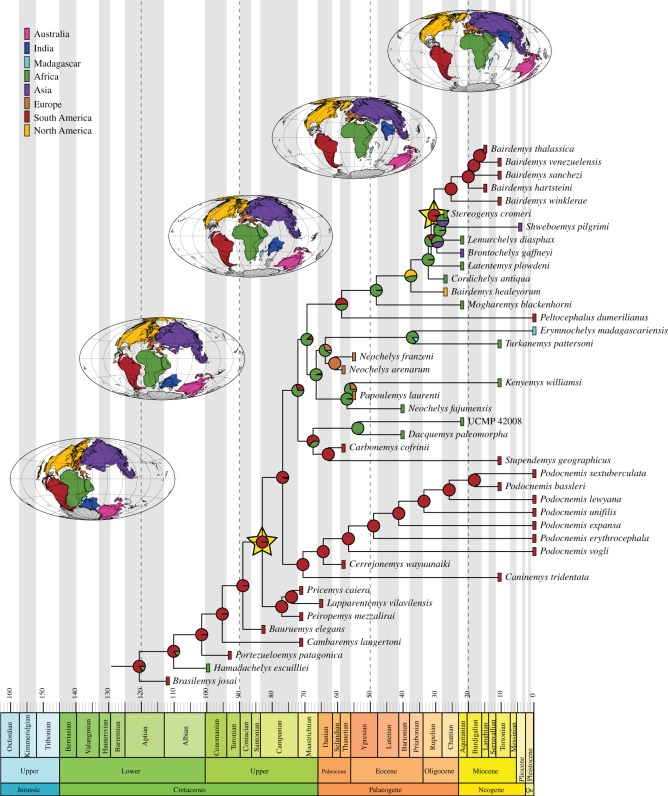
Ancestral area reconstructions and diversification shifts for the time-calibrated ‘original' tree including only Podocnemidoidae. Rectangles next to each terminal taxon represent its area distribution, pie charts represent the probabilities for ancestral area of nodes and yellow stars point to a node in which a diversification shift was found.

Shifts in three clades were recovered in all periods subsequent to time bin 1, Early Cretaceous (figure 2): Pleurodira (also in time bin 1), Podocnemidoidea and the clade including Cearachelyini + Bothremydini + Taphrosphyini. On time bin 4, Eocene (figure 3), two additional shifts were recovered: one for the clade including *Erymnochelys madagascariensis* and *Neochelys arenarum*, but not *Peltocephalus dumerilianus*, and another for the clade Peiropemydidae + Podocnemididae. The latter is also seen in all subsequent time bins, but the former is restricted to time bins 3 and 4. Two new shifts appear in time bins 6 and 7, one for the Stereogenyini clade including *Bairdemys venezuelensis* and *Stereogenys cromeri*, but not ‘*Bairdemys' healeyorum*, and one for Chelini. On time bin 7, a shift for the clade including all chelids but *Emydura macquarii* was recovered.

**Figure 4.**
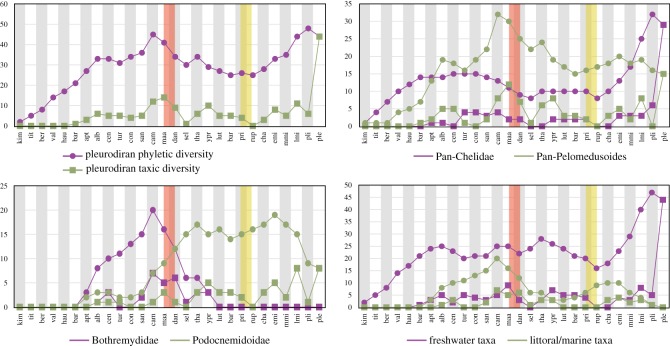
Diversity curves comparing distinct subsets of pleurodiran taxa. Circles and squares identify phyletic or taxic diversity curves, respectively. The orange and yellow bands highlight the Cretaceous–Palaeogene and Eocene–Oligocene mass extinctions, respectively.

The taxon diversity curves for Pleurodira show little perturbation, with an increasing diversity during the Early Cretaceous, a peak during the Campanian and Maastrichtian, a slight drop during the Early Paleocene and a fast recovery by the end of this period (figure 4). This pattern is replicated in the curve for Pelomedusoides, whereas Chelidae shows an increase by the Early Cretaceous followed by a relative stasis (figure 4), which is surely a consequence of its poor fossil record. The contrast between Bothremydidae and Podocnemidoidae (figure 4) reveals a higher and increasing diversity of the former from the Aptian to the Campanian, decreasing afterwards until its extinction during the Early Eocene. Although the first records of Podocnemidoidae also extends back to the Aptian, its diversity remains low during most of the Cretaceous, to rise more quickly after the Santonian, with a peak from the Late Paleocene to the Miocene, when it also starts to decrease (figure 4). Lastly, the contrast between the freshwater and littoral/marine taxa (figure 4) shows a predominance of freshwater forms, with a curve almost identical to that of Pleurodira, except for the Campanian peak that results from accounting the marine taxa. The diversity curve of littoral/marine pleurodires reflects the Bothremydini and Taphrosphyini diversification, beginning in the Late Cretaceous, and declining after the Campanian, but rising again from the Late Eocene to the Miocene (figure 4) with the diversification of Stereogenyini.

### 3.3. Biogeographical analysis

The LRT showed that M_2_ models were the best fitted across all three alternative topologies (table 1; see the electronic supplementary material). AICc model selection supports DIVALIKE-M_2_ in all cases (although DEC-M_2_ also performs well), stressing the power of cladogenetic range changes, i.e. vicariance and jump-dispersal (table 2; [55]), in explaining our data (see the electronic supplementary material). It is noteworthy that the inclusion of *x* as a free parameter improved model fit (table 1), supporting that the distance between the considered areas distinctly affects the pleurodiran lineages. Also, the inclusion of *j* as a free parameter consistently improved the fit in all models and topologies, suggesting that founder-event speciation is important to account for range changes in side-necked turtles.

**Table 1.** Pairwise comparison of the results of nested models using the ‘original' tree.

alternative model	LnL	d.f.	null model	LnL	d.f.	likelihood-ratio test *p*
DEC-M_1_	−191.7	3	DEC-M_0_	−209.3	2	3.0 × 10^−9^
DEC-M_2_	−135.8	4	DEC-M_1_	−191.7	3	4.0 × 10^−26^
DIVALIKE-M_1_	−203.5	3	DIVALIKE-M_0_	−231.3	2	8.6 × 10^−14^
DIVALIKE-M_2_	−135.6	4	DIVALIKE-M_1_	−203.5	3	2.4 × 10^−31^
BAYAREA-M_1_	−245.9	3	BAYAREA-M_0_	−253.5	2	9.8 × 10^−5^
BAYAREA-M_2_	−138.9	4	BAYAREA-M_1_	−245.9	3	1.7 × 10^−48^

**Table 2.** Summary of biogeographical stochastic mapping counts for Pleurodira using DIVALIKE-M_1_ and -M_2_ models showing the mean and standard deviations (s.d.) of different types of events estimated by those models.

		M_1_		M_2_	
mode	type	mean (s.d.)	%	mean (s.d.)	%
dispersal	range expansions	28.1(1.0)	22.0	0 (0)	0
	range contractions	0 (0)	0	0 (0)	0
	founder events	0 (0)	0	28.9 (0.9)	29.2
within-area speciation	sympatry	71.9 (1.2)	56.5	68.3 (0.9)	69.0
	subset speciation	0 (0)	0	0 (0)	0
vicariance	vicariance	27.1 (1.2)	21.3	1.8 (0.5)	1.8

Except for three nodes, i.e. Pan-Pleurodira, Pleurodira and Platychelyidae, widespread ancestors (i.e. ancestor occupying more than one defined area) are never favoured in our M_2_ models (see the electronic supplementary material). The African/Australian ancestral range for the Pleurodira node is best supported (although with low probability; figure 2), whereas for Pan-Chelidae an Australian range is favoured. The most probable ancestral area for Pan-Pelomedusoides and Pan-Pelomedusidae is Africa, whereas the ancestors of Pan-Podocnemididae probably dispersed to South America during the Early Cretaceous (figure 2). Other South America/Africa pairs are found during this period, for example in Araripemydidae, Euraxemydidae, Podocnemidoidea, Cearachelyini and *Hamadachelys *+ other Podocnemidoidae (figures 2 and 3), and two also during the Late Cretaceous, i.e. Podocnemididnae + Erymnochelyinae and the clade including *Dacquemys* and *Stupendemys* (figure 3). However, in the best fitted model, none of these joint distributions is favoured, making it unlikely that the descendant ranges are a result of vicariance [35,55]. The most probable ancestral area of bothremydids is Africa, and several dispersal events occurred from there, e.g. to India (*Sankuchemys *+ *Kurmademys*), Madagascar (*Kinkonychelys rogersi*), South America (Cearachelyini) and Europe (Bothremydini).

The summary of the BSM counts (table 2) shows that founder-event and vicariance represent, respectively, 29.2% and 1.8% of all events under the DIVALIKE-M_2_. On the other hand, under DIVALIKE-M_1_, vicariance accounts for 21.3%. All kinds of dispersal events (range expansion and founder events) represent 22.1% and 29.2% under DIVALIKE-M_1_ and -M_2_ models, respectively. The summary of BSM counts for DEC-M_1_ and -M_2_ models provides similar results (see the electronic supplementary material).

## 4. Discussion

### 4.1. Pleurodiran relationships

As previously mentioned, the analysis conducted here represents the largest (101 taxa/245 characters) and most inclusive phylogenetic study so far, to our knowledge, conducted for side-necked turtles. The resulting arrangement of the main lineages generally agrees with previous analyses (e.g. [12–14,21,23]), with some exceptions that reveal taxa with unstable relationships. The major extant (Chelidae, Pelomedusidae, Podocnemididae) and extinct (Araripemydidae, Euraxemyididae, Bothremydidae) lineages were mainly retrieved with relations similar to those previously proposed, although some discrepancies are seen.

The long-necked chelids are united into the clade Chelina (for phylogenetic definitions; see the electronic supplementary material), as in previous morphology-based studies [17,29,30], whereas molecular data studies suggest monophyletic Australasian and South American chelids instead [5,24–28]. Although seemingly more intuitive (but see below the discussion about ancestral area reconstructions), this result could not be replicated in any morphological study, even with the expansion of character and taxon samples seen here as well as in other studies [29,30,66]. Indeed, based on our data matrix, constraining South American and Australasian chelid clades resulted in 72 trees of 1162 steps, much longer than the original MPTs. Nine synapomorphies support the long-necked Chelina clade and only half of those are related to the cervical vertebrae. Thus, the morphological support for this clade is not exclusively related to their long necks. On the other hand, more recent molecular analyses have increased the sample of chelid taxa and gene sequences with unchanging results [5,25]. Indeed, there seems to be currently no objective reason to choose between those alternatives.

The position of Araripemydidae and Euraxemydidae (figure 1), forming the sister clade to Pelomedusoides (*sensu* [1]), agrees with a proposal by Meylan [21] that has never been replicated since then. Euraxemydids were retrieved inside Pelomedusoides in all other analyses, and Araripemydidae has been alternatively placed outside that group [12,23], inside it and closer to Podocnemididae [8,67], closer to Pelomedusidae [13], or even on the stem lineage of Pleurodira [14]. Forcing Euraxemydidae closer to Podocnemididae than to Araripemydidae or Pelomedusidae resulted in a tree only two steps longer (1131 steps). Similarly, forcing Araripemydidae inside Pelomedusoides, alternatively closer to Pelomedusidae or Podocnemididae, resulted in trees with 1133 and 1131 steps, respectively. Thus, although the position of Araripemydidae and Euraxemydidae in our strict consensus tree (figure 1) is well supported, forcing previous hypotheses does not result in much longer trees.

The position of *Atolchelys lepida*, the oldest known crown Pleurodira, is also controversial. It was retrieved inside Bothremydidae [8], or inside a clade with euraxemydids and *Sokatra antitra* [14]. Conversely, our results support both *S. antitra* and *A. lepida* as Pan-Podocnemididae (figure 1), but forcing *A. lepida* inside Bothremydidae requires only one additional step. Indeed, new data are needed to better evaluate the relations of this taxon.

Among Podocnemididae alternative arrangements have distinct biogeographical implications. First, *Caninemys tridentata* was retrieved as the sister taxon to the South American *Cerrejonemys + Podocnemis* clade [68], in contrast to previous analyses that supported an Erymnochelyinae affinity [13,14,41]. Another point of divergence between molecular and morphological datasets is seen for the extant *Podocnemis*, *Erymnochelys* and *Peltocephalus*. Molecular analyses [5,20,25,28,69] support the arrangement *Peltocephalus *+* *(*Erymnochelys *+ *Podocnemis*), whereas morphological data (including that presented here) suggest that *Erymnochelys* and *Peltocephalus* are closer to one another than to *Podocnemis* [13–15,21,37,38,41,67,68]. In almost all morphological analyses, *Erymnochelys* and *Peltocephalus* are sister taxa, whereas here *Peltocephalus* is sister to Stereogenyini and *Erymnochelys* is nested in a clade including other African and Europeans taxa (see also [15,68]). A close relationship between *Erymnochelys*, *Turkanemys* and *Kenyemys* was already suggested before (united in the so-called *Erymnochelys* group [70–74]), but has never been recovered in a phylogenetic analyses. In addition, *Neochelys* and *Papoulemys* were for the first time also included into that clade (figure 1). This arrangement has important implications for biogeographic analyses as *Erymnochelys* is positioned closer to an African–European rather than to a South American group.

### 4.2. Diversification of side-necked turtles

Molecular divergence time estimates suggest that the evolutionary history of Pleurodira began in the Late Jurassic, between 165 and 150 Ma [4,5,75], but the oldest unequivocal records of the group come from the Early Cretaceous (Barremian, *ca* 125 Ma) of Brazil [7,8]. The phyletic diversity in the last stages of the Early Cretaceous is low in comparison to that of the Late Cretaceous and Cenozoic (figure 4). As suggested for tetrapods in general [76], poor sampling might explain the low Early Cretaceous phyletic diversity of Pleurodira, especially taking into account the much older molecular clock estimates for the origin of the group. Nevertheless, the results of our analysis indicate that the two main pleurodiran lineages, Pan-Chelidae and Pan-Pelomedusoides, did not experience diversification rates that significantly differ from one another. However, the ghost lineages associated to Early Cretaceous taxa indicate that they were already established during this period (figures 2 and 3).

Two diversification shifts have been recognized in the Late Cretaceous (time bin 2), one related to the Podocnemidoidea clade and another for a clade within Bothremydidae. Because of the poorly sampled Early Cretaceous fossil record, the shifts detected for time bin 2 could be artefacts. Indeed, the Late Cretaceous fossil record of Podocnemidoidea is richer than that of other pleurodiran lineages, including Chelidae. However, the phylogenetic position of Early Cretaceous taxa such as *Prochelidella cerrobarcinae* and *Araripemys barretoi* indicates that a large number of non-podocnemidoidean Pelomedusoides and Chelidae lineages were already present in the Early Cretaceous (figures 2 and 3). Accordingly, as shifts are related to the balance of the tree, the appearance of further Podocnemidoidea lineages during the Late Cretaceous is interpreted as indicating a higher rate of diversification of this group related to other pleurodiran lineages, rather than as an artefact caused by the poor Early Cretaceous fossil record. In any case, this diversification shift can also be associated with the real diversification of Cearachelyini + Bothremydini + Taphrosphyini (figure 2).

The Cretaceous/Palaeogene boundary does not seem to correspond to a critical period of Pleurodira extinction or diversification (figure 4). With a few exceptions (e.g. Araripemyidae and *Galianemys*), most lineages crossed the Cretaceous/Palaeogene boundary (figures 2 and 3), albeit with a phyletic diversity reduction in some groups (e.g. Bothremydini). Furthermore, the lack of additional shifts in the Paleocene shows that the extinction of some lineages in the Late Cretaceous was not a trigger for diversifications in the first stage of the Cenozoic. Finally, the Cretaceous–Palaeogene mass extinction does not seem to affect the diversity of Pleurodira. Bothremydids experienced a decrease in diversity in the Mesozoic to Cenozoic passage, but this was already dropping after the Campanian and the trend continued until the Selandian (figure 4), suggesting that other factors were related to this decrease. Our results add to those on the North American turtle fauna (e.g. [77–79]) supporting the hypothesis that turtles were not much affected by the end-Cretaceous mass extinction.

The shift recovered for the clade Peiropemydidae + Podocnemididae in the Eocene is here interpreted as a real pattern, rather than an artefact caused by the extinction of lineages in previous intervals, especially given the relatively rich fossil record of bothremydids in the early Palaeogene. The Podocnemidoidea fossil record (approx. 20–30 known taxa) during the Paleocene and Eocene is also much richer than that of Chelidae (less than 5 taxa). However, only few lineages reach the end of the Eocene, and there is so far no record of pleurodires in the first stage of the Oligocene (Rupelian—*ca* 34 Ma; figure 4). Yet, it is not possible to assert if this is related to sampling biases, or to the Eocene–Oligocene extinction event [80] and the reduction of phyletic diversity in the Eocene associated to the temperature decrease during the Eocene (e.g. [81]). The phyletic diversity curve, however, suggests that pleurodires were not much affected by this event either (figure 4).

Two diversification shifts were detected in the Miocene time bin, one related to Chelini and another one associated with a clade within Stereogenyini. The fossil record of Pleurodira in the second stage of the Oligocene (Chattian, *ca* 25 Ma) is solely composed of stereogenyins. These Oligocene taxa and their respective ghost lineages have an influence in the tree balance of Stereogenyini in the Oligocene time bin, but no shift is detected in this interval. On the other hand, the subgroup of Stereogenyini for which the shift in the Miocene was detected (the least inclusive clade containing *Brontochelys gaffneyi* and *Bairdemys thalassica*) is majorly composed of Miocene representatives and ghost lineages from the Pliocene + Recent interval. Thus, even with the appearance of lineages of Stereogenyini in the Oligocene, the further diversification (i.e. changing in tree balance) of this subclade was still detected as a shift in our analysis, and is thus here interpreted as a real shift. Regarding the shift in the Miocene associated with Chelini, there are no Chelini taxa known from the Oligocene, in a way that the tree balance of Chelini shows no alteration between time bins 4 and 5, Eocene and Oligocene. Thus, the Miocene shift should have been seen with caution because of the low phyletic diversity of the group in previous stages, which might be related to sampling biases. However, there is a second aspect which indicates that the shift associated to this group is an artefact. The clade for which the shift was recognized also encompasses a part of the tree containing a great number of older representatives of Chelini that were already extinct by the Miocene (e.g. *Bonapartemys, Prochelidella, Chelodina alanrixi*). Thus, this shift is here understood as an artefact. Regarding the shift associated to Chelidae in time bin 7, there is also the presence of Chelidae taxa that were already extinct by the Pliocene + Recent interval. However, most of these also compose the Chelidae clade in the preceding time bin, Miocene, for which no shift was detected. Thus, we here interpret this as a real shift, associated to the radiation of chelid modern lineages (i.e. *Myuchelys, Elseya, Acanthochelys*).

### 4.3. Dispersal, not vicariance nor large-scale extinctions, drove the distribution of pleurodires

Previous studies dealing with the biogeographic history of pleurodires (e.g. [5,19,20,32,63,70]) suffered from two main drawbacks. First, except for that of Joyce *et al*. [63], they were conducted when fewer fossil taxa were known, or did not include fossil taxa at all [5]. For example, the studies of Noonan [20] and Romano & Azevedo [22] included only eight and six fossil taxa, respectively. Now, many more fossil pleurodires are known (e.g. [8,12–15,82]), providing a more detailed account of past distribution patterns (e.g. [16,83]) and more accurate age for the cladogenetic events, which largely influence the results of biogeographical reconstructions [33,84].

The second drawback is related to the theoretical and methodological turnover in historical biogeographic analyses from a search for common patterns and causes to the reconstruction of ancestral ranges with a broader consideration of several evolutionary processes, the event-based approaches [34,35,55,57]. The latter approach became dominant in the last two decades [55], assigning costs to evolutionary processes—e.g. vicariance, dispersal and extinction—and considering biogeographic patterns that minimize such total cost as optimal solutions [34]. Also, the advent of parametric approaches allowed the incorporation of information other than only distribution and topology, such as branch lengths and distance between areas, into complex models [5,34,55,61]. Finally, implementation of those models in a common likelihood framework enabled the choice of best fitted models, and the test of competing biogeographic scenarios [55,60].

Our analyses employed those recent methodological advancements on an up-to-date inventory of fossil taxa, and the results do not support either of the previously proposed explanatory hypotheses for the geographical distribution of pleurodires. By contrast, dispersal events are the dominant process underlying range changes under the best model (tables 1 and 2). This result is clearly dependent on the model: under M_1_ models (that exclude founder events) vicariance accounts for a similar proportion of range changes (table 2; see the electronic supplementary material for additional results). This is expected, because every model makes explicit assumptions about biogeographic processes and it has been shown that they largely affect ancestral area reconstructions [55]. Fortunately, the implementation under a common framework on BioGeoBears allows the comparison of competing models based on LRT to objectively choose the best fitted for each dataset [60]. DIVALIKE-M_2_ is clearly the best model for our data (table 1), so that we consider its reconstructions (figures 2 and 3) as the best representation of pleurodiran biogeographic history. Also, the BSM shows that dispersal events (including range expansion and founder events) occurred more commonly from South America to Africa and from Africa to South America, Europe and North America. This holds true even for M_1_ models (see the electronic supplementary material), reinforcing the high frequency of movements between those areas for pleurodires.

**Figure 5.**
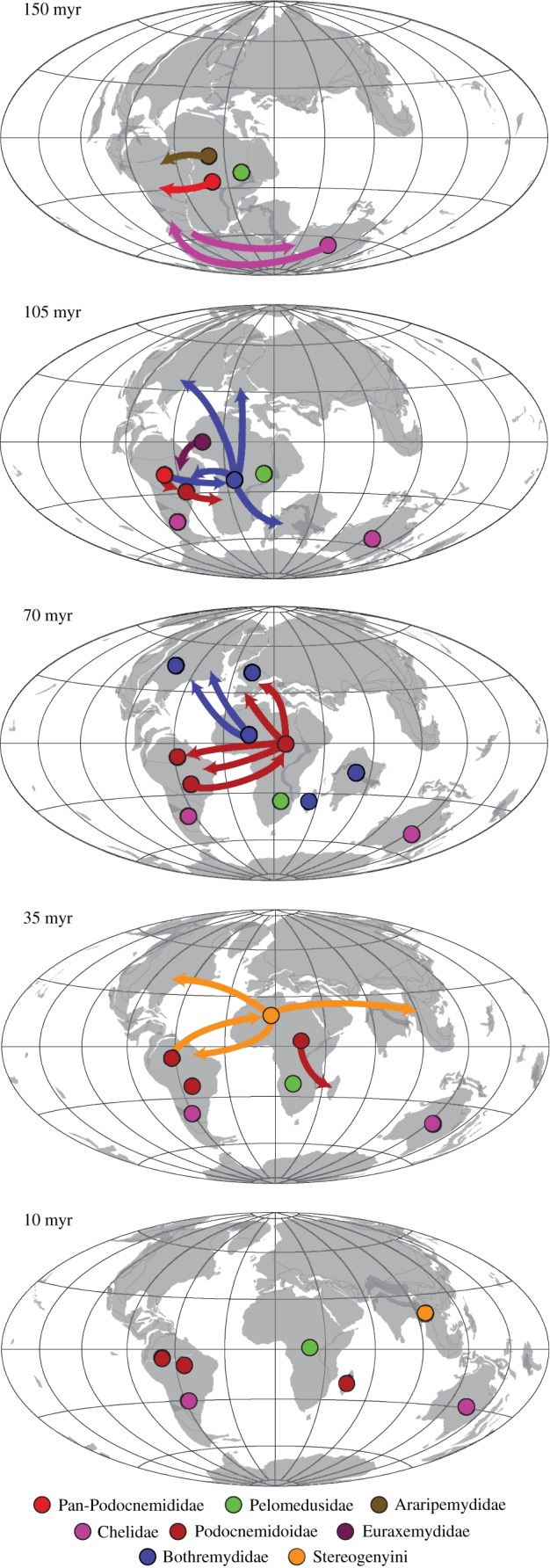
Palaeomaps summarizing the main dispersal events (arrows) of different pleurodiran groups (circles).

It is evident from the fossil record that, at least during the Cretaceous, pan-chelids and pan-pelomedusoids were restricted to southern and northern Gondwana, respectively [12,31,32,82], even though we did not define those as distinct areas for the analyses. We agree with Joyce *et al*. [63] that a desert zone, the Botucatu Desert [85], is the more likely barrier preventing the expansion of the southern Pan-Chelidae and the northern Pan-Pelomedusoides to one another's areas, because our hypothesis suggests that those lineages were already separated prior to the formation of the Paraná-Etendeka Volcanic Province (*ca* 137–127 Ma; [86]), considered a possible barrier by Romano & Azevedo [22], remaining separated longer than the duration of that event. It is well recorded that, in the northern part of the Paraná Basin, desert conditions continued to prevail after the volcanic event, as indicated by the aeolian sandstones of the Caiuá Group [87]. The ancestral area reconstructions using the ‘original' tree support an Australian origin for pan-chelids, which dispersed to South America still during the Early Cretaceous. *Chelodina* ancestors diverged from this South American lineage, dispersing back to Australia still during the Early Cretaceous (figure 5). The ‘molecular constrained' analyses also support a South American origin for Pan-Chelidae (see the electronic supplementary material) and all dispersal events between this area and Australia are also reconstructed to the Early Cretaceous. Although the distribution patterns of several other groups, including meiolaniid turtles [63,88,89], suggest that southern South America and Australia remained connected via Antarctica through the entire Cretaceous [90], our data suggest that by the end of the Early Cretaceous some kind of barrier prevented the dispersal of chelid turtles between those areas. The southward movement of the Antarctic continent, reaching latitudes higher than 70°S during the Aptian [91] and decreasing temperatures after the Cenomanian [92] may have been some factors limiting the presence of chelids in that continent, because turtles are rarer at such high latitudes and lower temperatures [63,92].

The analyses of the ‘original' or ‘molecular constrained' trees do not result in drastic changes in chelid ancestral area reconstructions (see the electronic supplementary material). It was previously supposed that chelids had diversified prior to the break-up of southern Gondwana, if results delivered by molecular analyses were favoured, or that ‘extensive dispersal' occurred more recently, when favouring the morphology-based topologies [82]. However, as fossil chelids are found deeply nested inside Chelina (figure 1), the divergence times between Australian and South American clades have been pushed back to the Early Cretaceous and only two dispersal events are needed to explain their current geographical distribution, both occurring prior to the separation of those continents (figure 2).

The biogeographic history of Pan-Pelomedusoides, by contrast, was dominated by the occurrence of areas of endemism for each clade, with several dispersal events to other areas (figure 5). The exception is Pelomedusidae, which was always endemic to continental Africa. Currently, some pelomedusids are found in Madagascar, the Arabic peninsula, the Seychelles archipelago and other small islands [9,93], but the absence of fossil records other than very scarce and fragmentary remains in continental Africa [72] preclude a more detailed account of the Pelomedusidae biogeographic history. Given the current data, we hypothesize that Pan-Pelomedusidae were always restricted to the African continent, and only recently dispersed transoceanically to those other areas.

Our results also show that the most recent ancestors of Araripemydidae, Euraxemydidae and Pan-Podocnemididae originally inhabited Africa, dispersing to South America during the Early Cretaceous (figure 5). The ancestors of Podocnemidoidae remained in the latter region, whereas those of Bothremydidae returned to the African continent. Bothremydids greatly diversified in this region (figure 2), but several taxa dispersed independently to other areas: at least once to Europe, India, Madagascar and back to South America, and at least three times to North America (figure 5). Our results highlight the great dispersion capability of bothremydids in accordance to the inferred marine or littoral habits of these pleurodires [16,47,63]. Bothremydidae was the most widespread group of side-necked turtles during the Cretaceous and Paleocene when they started to decline in diversity until their complete extinction by the Ypresian (figure 4).

The Podocnemidoidae have also been more widespread during some periods (e.g. Paleocene to Miocene), but our results show that, as bothremydids, they were mainly restricted to a few areas from which they dispersed to others (figure 5). The group was initially endemic to South America, but the ancestors of *Hamadachelys escuilliei* and Erymnochelyinae dispersed to Africa, respectively, during the Early and Late Cretaceous. The latter group greatly diversified in this continent, dispersing twice to Europe and once back to South America during the Paleocene (figure 5). The latter event originated *Peltocephalus dumerilianus* and Stereogenyini, which returned to Africa later. There are, however, almost equal probabilities for a dispersal event including only the ancestor of *P. dumerilianus* (figure 3). In this context, as with bothremydids and also in accordance to their inferred marine lifestyles [15,94,95], stereogenyins would have dispersed several times out of Africa, to North America, South America and East Asia (figure 5).

Even excluding the marine/littoral adapted bothremydids and stereogenyins, our results suggest several events of transoceanic dispersal during pleurodiran biogeographic history. Except for those lineages, which undertook long distance oceanic dispersals (e.g. from Africa to North America or East Asia), all the other events occurred across relatively short distances. Africa and South American exchanges do not occur after the Paleocene (except for Stereonyini) and other events are short dispersals from Africa to Europe or Madagascar. This hints at the possibility that, even not usually occupying brackish waters, pleurodires could tolerate transoceanic crossings, maybe carried out or rafted by ocean currents. Oceanic dispersals may have been more common causes of biogeographic range changes than previously thought, as already suggested for other groups such as tortoises [5,32], lizards [96], amphibians [62] and even invertebrates [97]. For pleurodires this should not come as a surprise, given the island distribution of some pelomedusids and chelids [9,93] and the results of a recent study [11], in which *Chelodina expansa* and *Emydura macquarii* individuals were exposed to saline conditions for long periods (50 days) without showing physiological problems. Finally, considering that stem-pleurodires could also have a certain tolerance to salty waters [6], this could be a more widespread feature in the group that could have facilitated the origin of groups more adapted to marine environments.

## 5. Conclusion

Our phylogenetic hypothesis is, to our knowledge, the most inclusive and well-sampled ever proposed for extinct pleurodiran turtles. Although pleurodiran relationships can be said to be stable owing to the general agreement between different hypotheses, some points of conflict still exist, especially between morphology and molecular data. Our diversification analysis suggests that pleurodires were not much affected by the Cretaceous–Palaeogene and Eocene–Oligocene mass extinction events. This result agrees with patterns observed for other testudine lineages and may represent a general trend for turtles.

Pelomedusoid extinct subclades show a greater number of diversification shifts in relation to Chelidae lineages, including the diversification shift related to bothremydids in the Early Cretaceous, the shift associated to the clade composed by Peiropemydidae + Podocnemididae in the Late Cretaceous and the one related to the clade within Stereogenyini in the Oligocene. Freshwater pleurodires apparently experienced steady diversification rates, whereas marine taxa peaked during the Late Cretaceous and Oligocene–Miocene periods (figure 4). In this sense, most pleurodire diversification shifts can be associated with the invasion of different niches, e.g. bothremydids and stereogenyins invading littoral or marine environments.

The current distribution of pleurodires cannot be fully understood using vicariance or large-scale extinctions as sole explanations. Although those may have affected the observed patterns, dispersal events seem to be the most important factor shaping the biogeography of side-necked turtles (table 2). Even though most dispersals occurred across relatively short distances, long distance dispersals were also common, especially among the littoral/marine-adapted bothremydids and stereogenyins (figure 5). As such, our hypothesis adds to recent results (e.g. [62,96,97]) indicating that transoceanic dispersals may be a much more common biogeographic event than previously thought.

## Supplementary Material

Phylogeny & biogeography of Pleurodira

## Data accessibility

The input files for the biogeographic analyses as well as the scripts used are available through GitHub Repository: https://github.com/gsferreirabio/pleurodira-biogeo.git.
