# Supplementary material for: Phylogeny, biogeography and diversification patterns of side-necked turtles (Testudines: Pleurodira)
Source: R Soc Open Sci. 2018 Mar 28;5(3):171773. doi: 10.1098/rsos.171773 (PMC5882704; doi:10.1098/rsos.171773)
Supplement: Phylogeny & biogeography of Pleurodira [file rsos171773supp1.pdf]

electronic supplementary material for

Phylogeny, biogeography and diversification patterns of side-necked turtles (Testudines: Pleurodira)

Gabriel S. Ferreira, Mario Bronzati, Max C. Langer, Juliana Sterli

correspondence to: gsferreirabio@gmail.com

This PDF file includes:

Section 1: Phylogenetic definitions of pleurodiran clades

Section 2: Unconstrained phylogenetic analysis

2.1. Detailed description of the procedures and results

2.2. Strict consensus tree with node numbers

2.3. Bootstrap, Jacknife & Bremer support information

2.4. List of characters

2.5. List of common synapomorphies

Section 3: Constrained phylogenetic analysis results

3.1. Detailed description of the results

3.2. Constrained clades

3.3. Strict consensus tree with node numbers

Section 4: Additional trees

4.1. Procedure to create fully dichotomic trees

4.2. Supertree

4.3. 'Non-marine taxa' tree

4.4. Time bin subtrees for diversification shifts analyses

Section 5: Biogeographic analyses

5.1. Ancestral area reconstruction results and original outputs

5.2. Biogeographic stochastic mapping of DIVALIKE M_1 and M_2 models outputs

Section 6: Supplementary references

Section 7: Illustration plates for new characters

Section 1: Phylogenetic definitions of pleurodiran clades

Pleurodira Cope, 1865

Definition (based on Joyce *et al*., 2004): Pleurodira refers to the crown-clade containing the last common ancestor of *Chelus fimbriatus* (Schneider, 1783), *Pelomedusa subrufa* (Bonnaterre, 1789) and *Podocnemis expansa* (Schweigger, 1812) and all its descendants.

Pan-Pleurodira Joyce, Parham & Gauthier, 2004

Definition (based on Joyce *et al*., 2004): Pan-Pleurodira refers to the most inclusive clade containing *Chelus fimbriatus*, *Pelomedusa subrufa* and *Podocnemis expansa*, but not *Testudo graeca* Linnaeus, 1758, *Chelonia mydas* (Linnaeus, 1758) or *Trionyx triunguis* (Forskål, 1775).

Chelidae Lindhom, 1929

Definition (amended from Joyce *et al*., 2004): Chelidae refers to the crown-clade containing the last common ancestor of *Chelus fimbriatus*, *Chelodina longicollis* (Shaw, 1794), *Emydura macquarii* (Gray, 1830) and *Pseudemydura umbrina* Siebenrock, 1901 and all its descendants.

Pan-Chelidae Joyce, Parham & Gauthier, 2004

Definition (amended from Joyce *et al*., 2004): Pan-Chelidae refers to the most inclusive clade containing *Chelus fimbriatus*, *Chelodina longicollis*, *Emydura macquarii* and *Pseudemydura umbrina*, but not *Pelomedusa subrufa* or *Podocnemis expansa*.

Chelini Gaffney, 1977, converted clade name

Definition: Chelini refers to the least inclusive clade containing the last common ancestor of *Chelus fimbriatus*, *Hydromedusa maximiliani* (Mikan, 1825), *Phrynops geoffroanus* (Schweigger, 1812) and *Platemys platycephala* (Schneider, 1792) and all its descendants.

Chelina Gaffney, 1977, converted clade name

Definition: Chelina refers to the most inclusive clade containing *Chelus fimbriatus*, *Chelodina longicollis* and *Hydromedusa maximiliani*, but not *Phrynops geoffroanus* or *Emydura macquarii*.

Araripemydidae Price, 1973, converted clade name

Definition: Araripemydidae refers to the most inclusive clade containing *Araripemys barretoi* Price, 1973, but not *Euraxemys essweini* Gaffney, Tong & Meylan, 2006, *Pelomedusa subrufa* or *Podocnemis expansa*.

Euraxemydidae Gaffney, Tong & Meylan, 2006, converted clade name

Definition: Euraxemydidae refers to the most inclusive clade containing *Euraxemys essweini*, but not *Araripemys barretoi*, *Pelomedusa subrufa* or *Podocnemis expansa*.

Pelomedusoides Broin, 1988

Definition (based on Joyce *et al.*, 2004): Pelomedusoides refers to the crown-clade containing *Pelomedusa subrufa* and *Podocnemis expansa* and all its descendants.

Pan-Pelomedusoides Joyce, Parham & Gauthier, 2004

Definition (amended on Joyce *et al.*, 2004): Pan-Pelomedusoides refers to the least inclusive clade containing *Pelomedusa subrufa* and *Podocnemis expansa*, but not *Chelus fimbriatus* or *Emydura macquarii*.

Pelomedusidae Cope, 1868

Definition (based on Joyce *et al.*, 2004): Pelomedusidae refers to the crown-clade containing the last common ancestor of *Pelomedusa subrufa* and *Pelusios subniger* (Bonnaterre, 1789), and all its descendants.

Pan-Pelomedusidae Joyce, Parham & Gauthier, 2004

Definition (amended on Joyce *et al.*, 2004): Pan-Pelomedusidae refers to the least inclusive clade containing *Pelomedusa subrufa* and *Pelusios subniger*, but not *Podocnemis expansa* or *Chelus fimbriatus*.

Podocnemidoidea Broin, 1988

Definition (same as in França & Langer, 2006): Podocnemidoidea refers to the least inclusive clade containing the last common ancestor of *Podocnemis expansa* and *Bothremys cooki* Leidy, 1865, and all its descendants.

Bothremydidae Baur, 1891

Definition (same as in Joyce *et al.*, 2016a): Bothremydidae refers to the most inclusive clade containing *Bothremys cooki*, but not *Pelomedusa subrufa* or *Podocnemis expansa*.

Bothremydini Gaffney, Tong & Meylan, 2006

Definition (same as in Joyce *et al.*, 2016a): Bothremydini refers to the most inclusive clade containing *Bothremys cooki*, but not *Cearachelys placidoi* Gaffney, Almeida Campos & Hirayama, 2001, *Kurmademys kallamedensis* Gaffney, Chatterjee & Rudra, 2001, or *Taphrosphys sulcatus* (Leidy, 1856).

Cearachelyini Gaffney, Tong & Meylan, 2006

Definition (same as in Joyce *et al.*, 2016a): Cearachelyini refers to the most inclusive clade containing *Cearachelys placidoi*, but not *Bothremys cooki*, *Kurmademys kallamedensis*, or *Taphrosphys sulcatus*.

Taphrosphyini Gaffney, Tong & Meylan, 2006

Definition (same as in Joyce *et al.*, 2016a): Taphrosphyni refers to the most inclusive clade containing *Taphrosphys sulcatus*, but not *Bothremys cooki*, *Cearachelys placidoi*, or *Kurmademys kallamedensis*.

Podocnemidoidae Broin, 1988

Definition (same as in França & Langer, 2006): Podocnemidoidea refers to the most inclusive clade containing *Podocnemis expansa*, *Peltocephalus dumerilianus* (Schweigger, 1812) and *Erymnochelys madagascariensis* (Grandidier, 1867), but not *Cearachelys placidoi*, *Bothremys cooki* or *Taphrosphys sulcatus*.

Peiropemydidae Gaffney, Meylan, Wood, Simons and Campos, 2011

Definition (same as in Ferreira *et al.*, *in press*): Peiropemydidae refers to the most inclusive clade containing *Peiropemys mezzalirai* Gaffney, Meylan, Wood, Simons and Campos 2011 and *Lapparentemys vilavilensis* (Broin, 1971), but not *Podocnemis expansa.*

Podocnemididae Baur, 1893

Definition (based on Joyce *et al.*, 2004): Podocnemididae refers to the crown-clade containing the last common ancestor of *Podocnemis expansa*, *Peltocephalus dumerilianus* and *Erymnochelys madagascariensis*, and all its descendants.

Pan-Podocnemididae Joyce, Parham & Gauthier, 2004

Definition (amended from Joyce *et al.*, 2004): Pan-Podocnemididae refers to the most inclusive clade containing *Podocnemis expansa*, *Peltocephalus dumerilianus* and *Erymnochelys madagascariensis*, but not *Pelomedusa subrufa* and *Chelus fimbriatus*.

Podocnemidinae Cope, 1868, converted clade name

Definition: Podocnemidinae refers to the most inclusive clade containing *Podocnemis expansa*, but not *Erymnochelys madagascariensis*.

Erymnochelyinae Broin, 1988, converted clade name

Definition: Erymnochelyinae refers to the most inclusive clade containing *Erymnochelys madagascariensis*, but not *Podocnemis expansa*.

Stereogenyini Gaffney, Meylan, Wood, Simons and Campos, 2011, converted clade name

Definition: Stereogenyini refers to the least inclusive clade containing the last common ancestor of *Stereogenys cromeri* (Andrews, 1901), *Bairdemys hartsteini* Gaffney & Wood, 2002, and *Mogharemys blackenhorni* (Dacqué, 1912) and all its descendants.

Section 2: Unconstrained phylogenetic analysis

2.1. Detailed description of the procedures and results

The two stem-pleurodires included in our matrix, *Platychelys oberndorferi* and *Notoemys laticentralis*, are grouped in the Platychelyidae clade (Fig. 1, S1), supported (BS = 4) by five synapomorphies, including an articulation facet on the anterior margin of first thoracic rib (ch. 188) and a fontanella between the hyo- and hypoplastra (ch. 226). The crown-group Pleurodira (BS = 6) is diagnosed by eight synapomorphies, such as a closed foramen jugulare posterius (ch. 112) and postzygapophyses of the cervical vertebrae elevated on the neural spine (ch. 164). Although Chelidae has a low Bremer support (BS = 1), it is supported by twelve synapomorphies, including the absence of the quadratojugal (ch. 15) and a concave lateral surface of the squamosal (ch. 38). The South American chelids (Chelini; Fig. 1, S1) were grouped in a short- and a long-necked clades, the latter including also the Australasian *Chelodina colliei*. The other Australasian chelids are retrieved as successive sister-taxa to the South American clade (Fig. 1, S1). All extinct chelids included here, except *Chelus colombianus*, were grouped in the same clade (BS = 1), sister to *Hydromedusa* spp. (Fig. 1, S1), supported by a wider than long mesoplastra (ch. 225), pill-shaped pubic scars (ch. 239), and two other synapomorphies.

The Australasian chelids, as in previous phylogenetic analyses based on morphological data (e.g. Gaffney, 1977; Bona & de la Fuente, 2005; de la Fuente *et al.*, 2017a), appear distributed as successive sister taxa to the South American chelid clade (Chelini). The grouping of *Hydromedusa* + *Yaminuechelys* was supported by three synapomorphies in Bona & de la Fuente's (2005) analysis and here those same characters (ch. 73, 108, 157, and 174) plus the neural series extending to costal 8 (ch. 175) support the phylogenetic position of the extinct chelids herein. Previous studies considered *Bonapartemys bajobarrealis* and *Prochelidella cerrobarcinae* more closely related to *Phrynops* spp. and *Acanthochelys* spp., respectively (Lapparent de Broin & de la Fuente, 2001; de la Fuente *et al.*, 2011). A recent phylogenetic analysis (de la Fuente *et al.*, 2017a) included *Bonapartemys bajobarrealis* for the first time in a phylogenetic context, retrieved it nested within a clade sister to that containing all extant chelids, contrasting to our results.

Pan-Pelomedusoides (BS = 3) is diagnosed by the absence of nasal (ch. 3) and splenial (ch. 133) bones, and eleven other synapomorphies. Araripemydidae (BS = 9) and Euraxemydidae (BS = 4) are grouped together as part of the stem lineage to crown Pelomedusoides. Both Pelomedusoides (BS = 1) and Araripemydidae + Euraxemyidade (BS = 1) are supported by three synapomorphies. Araripemydidae was previously recovered variably inside (Gaffney *et al.*, 2011; Romano *et al.*, 2014; Menegazzo *et al.*, 2015) or outside (Meylan, 1996; França & Langer, 2006; Gaffney *et al.*,

2006; Cadena, 2015) Pelomedusoides, but Euraxemydidae is almost always retrieved inside this clade (except for Meylan, 1996).

As in all previous analyses (e.g. Gaffney *et al.*, 2006, 2011; Cadena, 2015), no taxon is recovered in the stem-lineage of Pelomedusidae, resulting in a large gap from the Barremian to the first records of the extant taxa (Fig. 2). *Sokatra antitra* and *Atolchelys lepida* were recovered as successive sister-taxa to Podocnemidoidea (Bothremydidae + Podocnemidoidae). The position of *Atolchelys lepida* is still controversial: in the original description (Romano *et al.*, 2014) this taxon is retrieved as sister to all bothremydids and in a later analysis (Cadena, 2015) it is inside a clade also including *So. antitra* and euraxemyidids.

The interrelations of Bothremydidae are problematic in two points. The first one is related to the Kurmademydini clade (Gaffney *et al.*, 2006, 2009; Rabi *et al.*, 2012; Joyce *et al.*, 2016), which is not supported by our analyses (Fig. 1, S1). A paraphyletic "Kurmademydini" was already retrieved by previous analyses (Romano *et al.*, 2014; Cadena, 2015), but in these *Kinkonychelys rogersi* and *Kurmademys kallamedensis* are grouped in a clade, whereas our result support the former as closer to the other bothremydids. The second point of disagreement is related to the position of Foxemydina which is alternatively closer to Bothremydini (Gaffney *et al.*, 2006; Cadena *et al.*, 2012a; Rabi *et al.*, 2012) or sister to the clade including Taphrosphyni + Bothremydini (Cadena, 2015; Joyce *et al.*, 2016). Our results agree with the former hypothesis with a good support from morphological characters (Fig. S1-S6).

The crown-clade Podocnemididae (BS = 2) splits in two lineages, Podocnemidinae including *Podocnemis spp.*, *Cerrejonemys wayuunaki* and *Caninemys tridentata*, and Erymnochelyinae, including all other podocnemidids (Fig. 1, S1). The position of *Ca. tridentata*, from the Miocene of South America, is unusual, being previously recovered among Erymnochelyinae instead (Meylan *et al.*, 2009; Gaffney *et al.*, 2011; Cadena, 2015). Several taxa occur between the extant *Erymnochelys madagascariensis* and *Peltocephalus dumerilianus*. Even though a *Erymnochelys*-group was proposed elsewhere (Broin, 1988; Lapparent de Broin, 2000a, 2000b. Pérez-García & Lapparent de Broin, 2015; Pérez-García *et al.*, 2017) which would include fossil taxa closer to *Erymnochelys* than to other extant podocnemidids, it was based solely on a brief anatomical diagnose ("gulars are in contact posteromedially, and located posteriorly to a reduced intergular that they frame"; Pérez-García *et al.*, 2017), this was never supported by phylogenetic analyses, which commonly group *Erymnochelys* and *Peltocephalus* as sister-taxa (Gaffney *et al.*, 2011; Cadena, 2015). The *Erymnochelys*-group, however, has a different composition than the group retrieved here. Aside from *Kenyemys williamsi* and *Turkanemys pattersoni* (the latter retrieved here as sister-taxa to *Erymnochelys madagascariensis*, Fig. 1, S1) it also included

'Neochelys' fajumensis, *'Podocnemis' aegyptiaca*, and three *Eocenochelus* taxa, but excluded *Papoulemys laurenti* (see below discussion about this taxon) and *Neochelys* (Pérez-García *et al.*, 2017). Here, both the later are also closer to *Erymnochelys* than to other extant podocnemidids. This clade is supported by gular scutes with similar sizes to intergular (ch. 231) that reach the entoplastron bone (ch. 234).

The European erymnochelyin *Papoulemys laurenti* was recently reallocated inside *Neochelys* based on a redescription that identified common features between those taxa (Pérez-García & Lapparent de Broin 2015). Unlike the *Erymnochelys*-group, this hypothesis was supported by one phylogenetic analysis (Cadena, 2015). Here, however, *Papoulemys laurenti* is retrieved closer to *Kenyemys williamsi* and *'Neochelys' fajumensis* than to the included *Neochelys* taxa, namely *N. arenarum* and *N. franzeni*. This is supported only by wider than long cranial margin of intergular (ch. 242), while *Neochelys* is diagnosed by slightly elongated heart-like shaped interparietal scale (ch. 27) and their position closer to *Erymnochelys* and *Turkanemys* than to *Papoulemys laurenti* is supported by a foramen palatinum posterius restricted to the palatine (ch. 71) and neural series reaching costal plates 6 (ch. 175). Based on our analyses, to reallocate *Papoulemys laurenti* into the same genus as *Neochelys arenarum* and *N. franzeni* would require to do the same with *Turkanemys pattersoni* and *Erymnochelys madagascariensis*. Hence, we choose to maintain the status of *Papoulemys* as a valid genus, with *Papoulemys laurenti* as its type and only included species.

Finally, *Peltocephalus dumerilianus* is recovered as the sister-taxon to Stereogenyini (Gaffney *et al.*, 2011), supported by five synapomorphies, including an anterior pit on the ventral surface of the premaxillae (ch. 46). This affinity was already hinted by a previous analysis (Ferreira *et al.*, 2015), that focused on Stereogenyini. The latter group includes Stereogenyita and Bairdemydita clades (Fig. 1, S1), with *Mogharemys blackenhorni*, *Cordichelys antiqua*, *Latentemys plowdeni* and *"Bairdemys" healeyorum* as successive sister-taxa to them.

2.2. Strict consensus tree with node numbers:

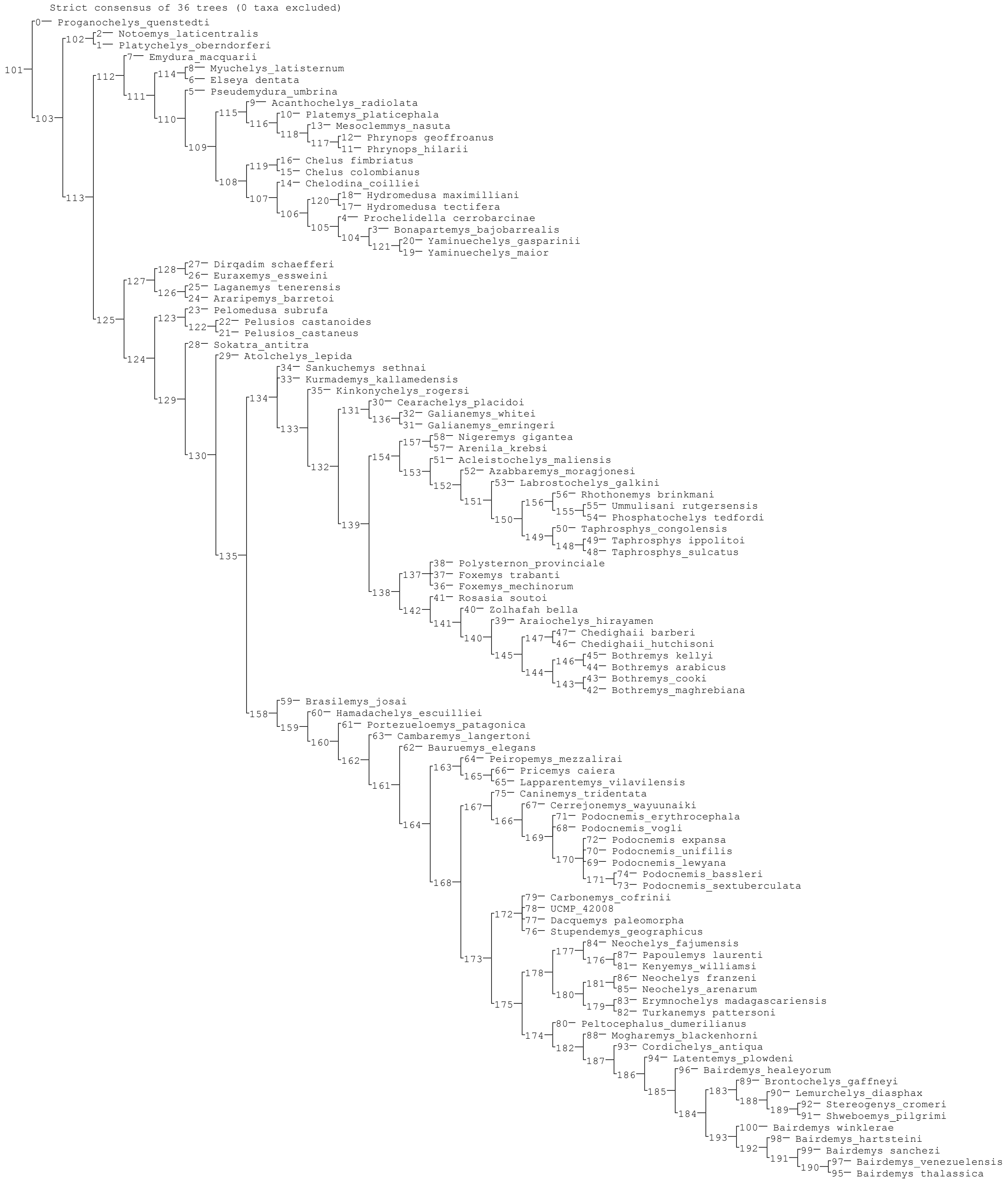

Supplementary Figure 1. Strict consensus tree of 36 most parsimonious trees of 1128 steps.

2.3. Bootstrap, Jacknife & Bremer support information

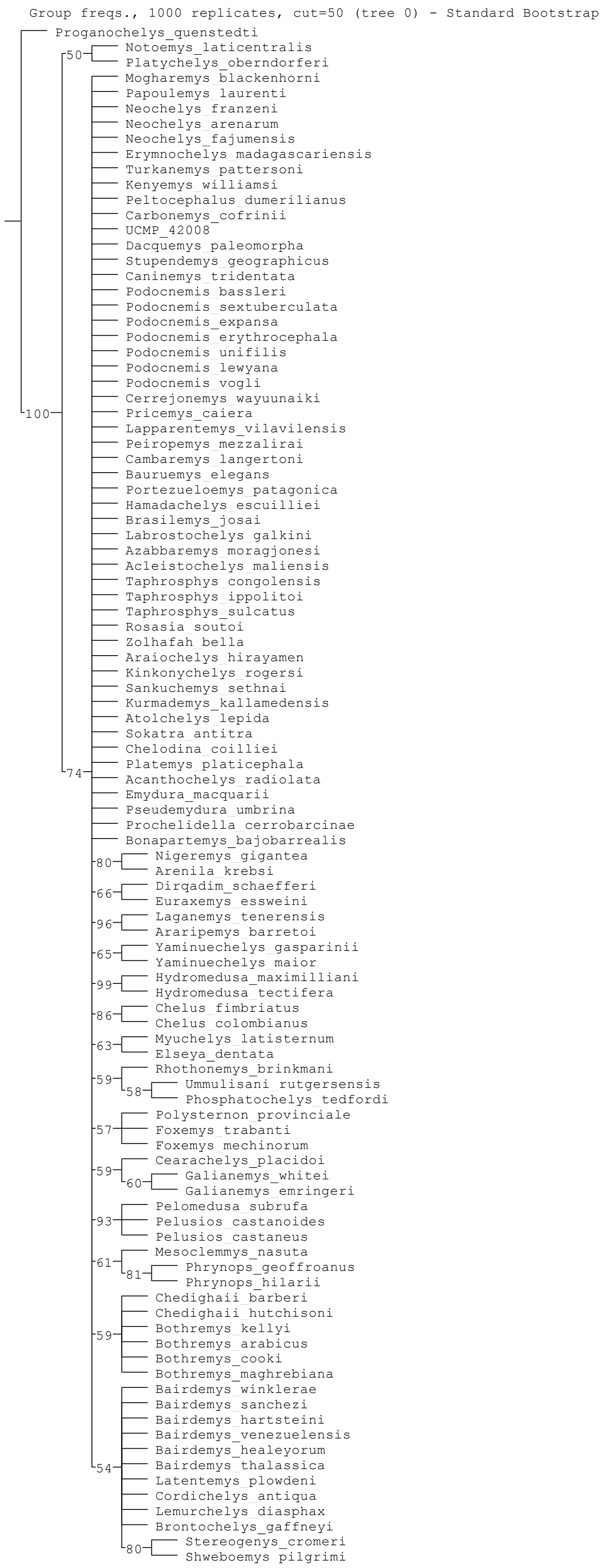

Supplementary Figure 2. Group frequencies Bootstrap values of 1000 replicates for the unconstrained analysis.

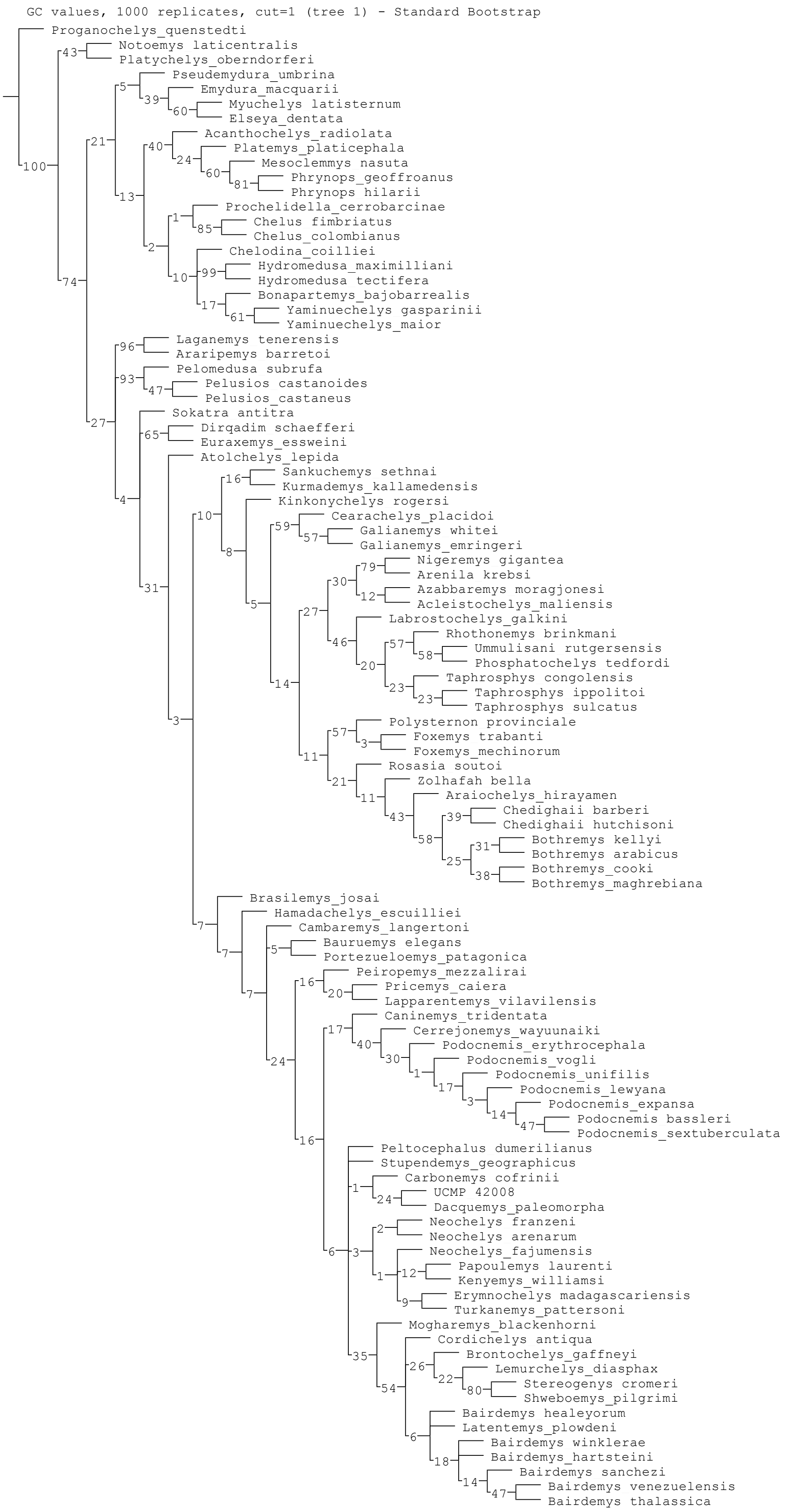

Supplementary Figure 3. GC Bootstrap values of 1000 replicates for the unconstrained analysis.

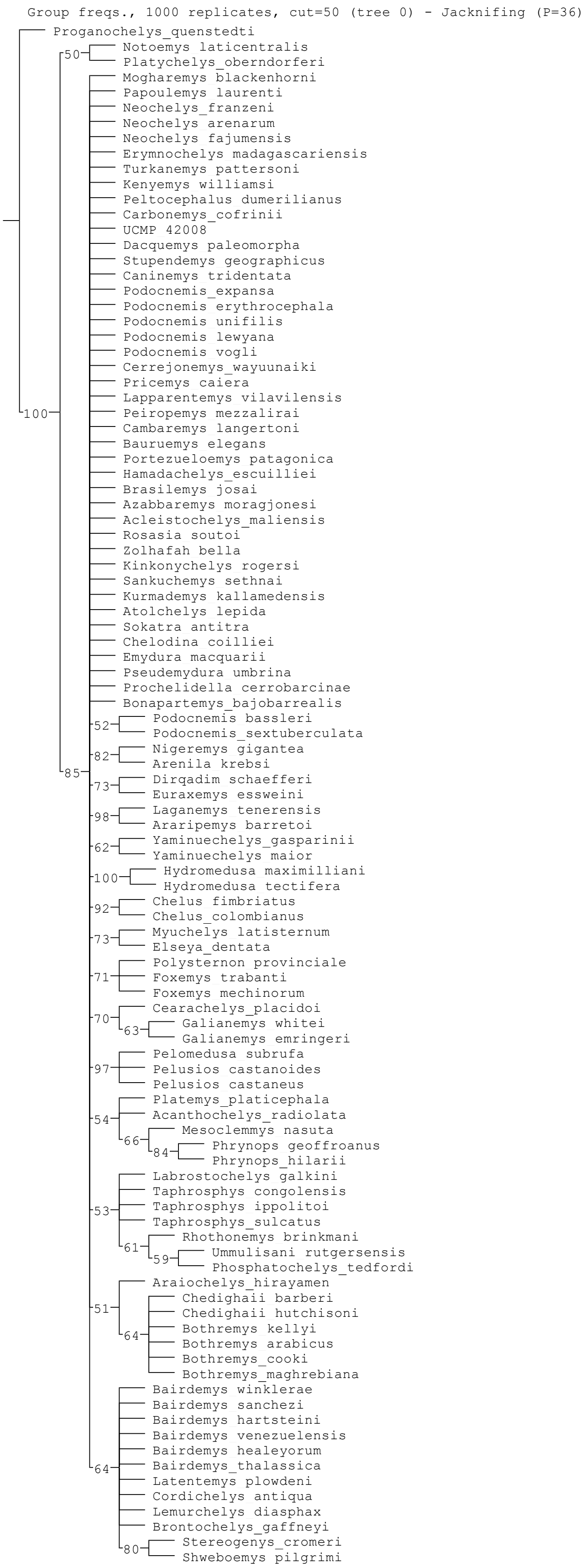

Supplementary Figure 4. Group frequencies Jacknife values of 1000 replicates for the unconstrained analysis.

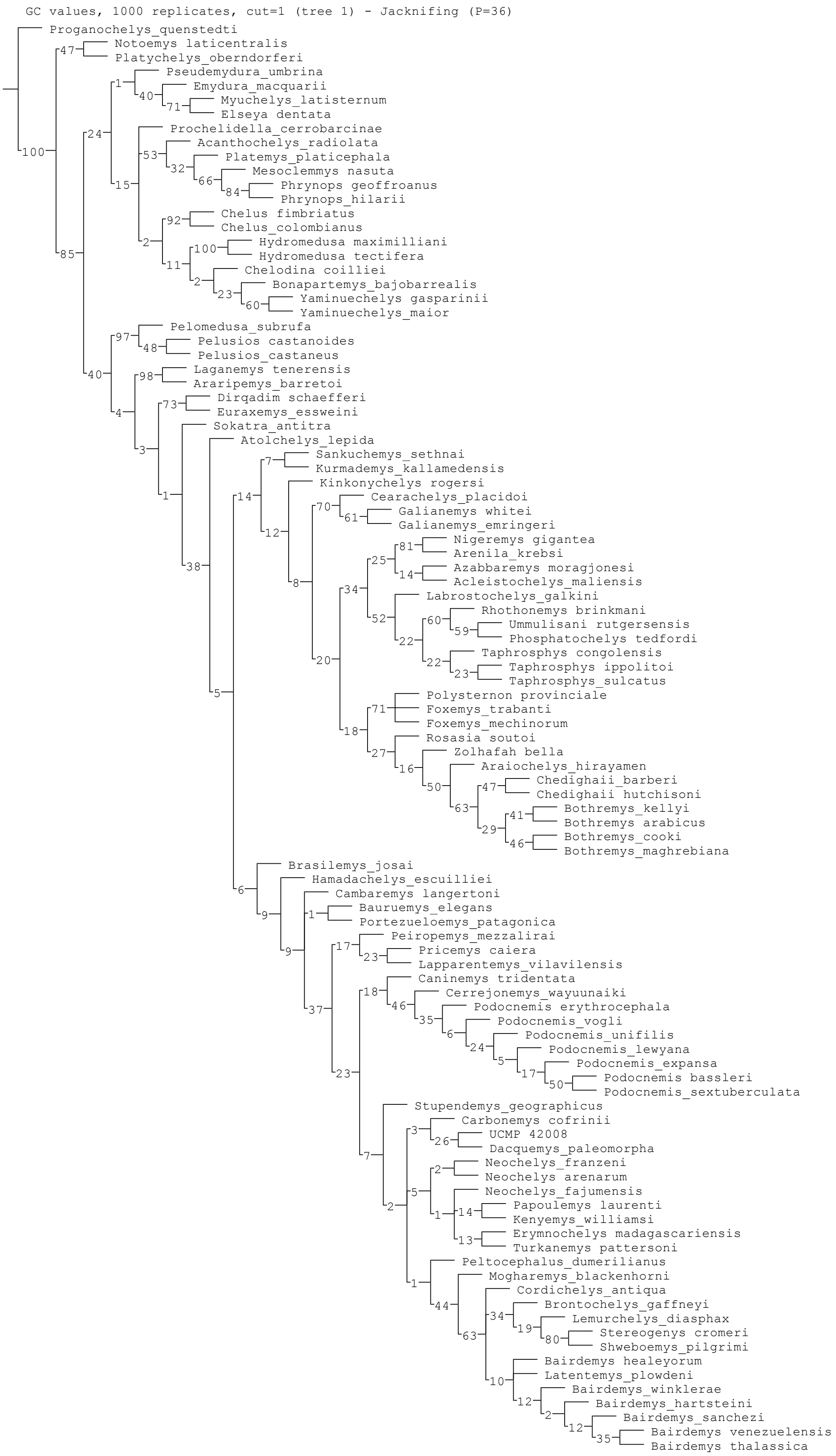

Supplementary Figure 5. GC Jacknife values of 1000 replicates for the unconstrained analysis.

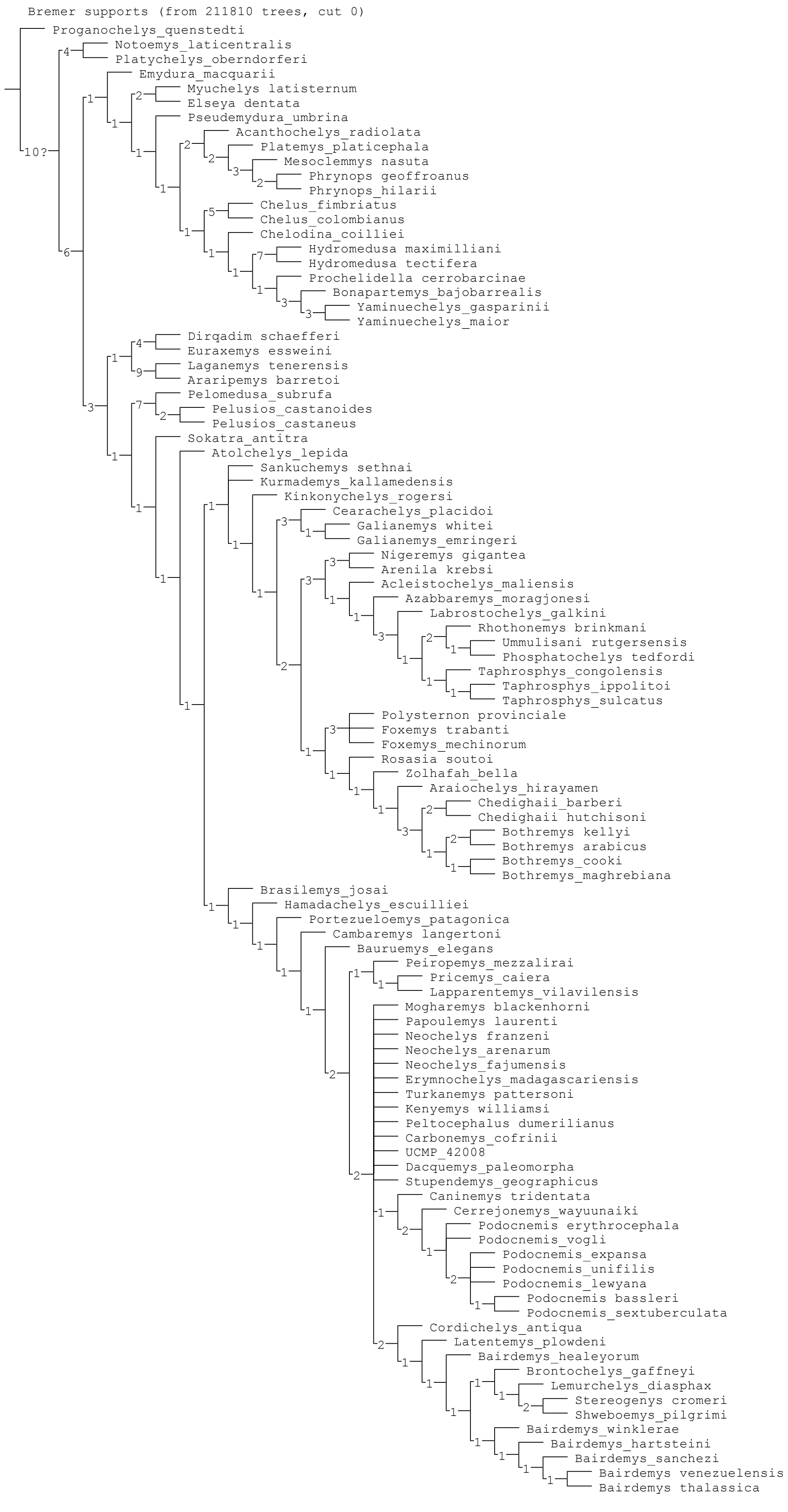

Supplementary Figure 6. Bremer support values for the unconstrained analysis obtained using TBR from existing trees retaining suboptimal trees by 10 steps.

2.4. List of characters

Reference abbreviations:

BF05: Bona & de la Fuente (2005)

C08: Cadena et al. (2008)

C10: Cadena et al. (2010)

C12: Cadena et al. (2012)

C13: Cadena et al. (2013a)

C13*: Cadena et al. (2013b)

C15: Cadena (2015)

CP15: Cadena & Parham (2015)

D13: Dummont Junior (2013)

F03: de la Fuente (2003)

F17: de la Fuente et al. (2017)

G77: Gaffney (1977)

G06: Gaffney et al. (2006)

G11: Gaffney et al. (2011)

J07: Joyce (2007)

JB04: Joyce & Bell (2004)

M09: Meylan et al. (2009)

P13: Pérez-García & Lapparent de Broin (2013)

T09: Thomson & Georges (2009)

Characters:

1. Skull flattened (BF05Ch03, G77Ch09)
    0 high skull (as in *Peltocephalus dumerilianus*)
    1 flat skull (as in *Pelusios castanoides*)
    2 extremely flattened skull (as in *Hydromedusa tectifera*)

2. Dorsal margin of apertura narium externa (G06Ch06, C12Ch05)
    0 straight, broadly convex margin (*Pelomedusa subrufa*)
    1 narrow midline process, at least partially dividing external nares (*Bothremys cooki*)

3. NA, nasals (G06Ch01, G11Ch01, F03Ch02, G77Ch01)
    0 present (*Emydura macquarii*)
    1 absent (*Pelusios castanoides*)

4. NA, meet on midline in dorsal view (BF05Ch04, G77Ch02)
    0 nasals meet (*Hydromedusa tectifera*)

1 nasals do not meet, completely separated by frontal process (*Chelodina oblonga*)

5. PF, preorbital skull broad (G06Ch05, C12Ch08, C15Ch04)
0 narrow, equal or less than 90º, as in *Podocnemis expansa*
1 very broad, more than 90º, as in *Bothremys cooki*

6. PF, meet on midline in dorsal view (G06Ch04)
0 no (*Proganochelys quenstendti*)
1 yes (*Pelusios castanoides*)

7. PF, pf-pal contact (G06Ch07, C12Ch08)
0 no contact (*Galianemys emringeri*)
1 contact present in anterior wall of fossa orbitalis (*Bothremys maghrebiana*)

8. PF, pf-pa contact (G06Ch10)
0 no contact (*Galianemys emringeri*)
1 present (*Phosphatochelys tedfordi*)

9. FR, interorbital groove (G11Ch03, C12Ch07)
0 no groove, smooth surface as in *Peltocephalus dumerilianus*
1 surface with a groove as in *Podocnemis expansa*

10. FR, orbital position (G06Ch11, G11Ch02)
0 facing laterally, anterolaterally (*Bairdemys venezuelensis*)
1 facing dorsolaterally (*Elseya dentata*)
2 facing dorsally (*Chelodina coillei*)

11. FR, cranial process of frontal (TG09Ch10)
0 no process, smooth cranial edge (*Podocnemis expansa*)
1 projecting cranial process (*Elseya dentata*)

12. FR, prefrontal/frontal (G11Ch04)
0 flat or slight convex (*Podocnemis expansa*)
1 strongly convex dorsally (*Bairdemys venezuelensis*)

13. FR, foramen interorbitale (G06Ch12)
0 high (*Galianemys emringeri*)
1 low (*Bothremys maghrebiana*)

14. Anteroventral emargination, extension in lateral view (G11Ch11, BF05Ch01, BF05Ch08, G77Ch10) (ordered)
0 absent or slight (*Peltocephalus dumerilianus*)
1 reaches ventral margin level of orbit (*Hamadachelys escuilliei*)
2 reaches dorsal margin level of cavum tympani (*Lapparentemys villavilensis*)
3 reaches above level of orbit or parietal (*Elseya dentata*)

15. QJ, quadratojugal (C12Ch02, F03Ch04, BF05Ch02)
0 absent (*Elseya dentata*)
1 present (*Podocnemis expansa*)

16. QJ-PA contact (G06Ch13, G11Ch05)
0 contact present (*Podocnemis expansa*)

1 no contact (*Pelomedusa subrufa*)

17. QJ-PA contact extension (G06Ch13, G11Ch05, C12Ch04, F03Ch05)
0 short contact (*Bauruemys elegans*)
1 long contact (*Podocnemis expansa*)

18. Posterodorsal emargination, extension in dorsal view (G11Ch04, BF05Ch06, BF05Ch07, G77Ch08) (ordered)
0 no emargination, edge of the emargination caudal or on the same plane of condylus occipitalis (*Dacquemys palaeomorpha*)
1 edge of the emargination rostral to the condylus occipitalis plane (*Podocnemis expansa*)
2 edge rostral to condylus occipitalis plane and rostral wall of the otic chamber visible in dorsal view (*Pelomedusa subrufa*)

19. PA, dorsal portion in relation to adductor fossa (TG09Ch15, G77Ch10) (ordered)
0 broadly covers adductor fossa (*Podocnemis expansa*)
1 covers central area of adductor fossa (*Elseya dentata*)
2 covers little of adductor fossa (*Phrynops geoffroanus*)
3 parietal does not cover adductor fossa (*Hydromedusa tectifera*)

20. PA, Lateral edges of parietals (BF05Ch09, G77Ch11)
0 parallel or subparallel (*Podocnemis expansa*)
1 pentagon-shaped parietal, lateral edges diverging (*Bothremys magrebiana*)
2 tapering posteriorly (*Hydromedusa tectifera*)
3 reduced in their medial portion ('wasp-waisted') (*Acantochelys radiolata*)

21. PA, sulcus palatinopterygoideus (G06C17)
0 high (*Galianemys emringeri*)
1 low due to thicker pa, po (*Bothremys maghrebiana*)

22. PA, enters orbital margin (G06Ch18)
0 no (*Galianemys emringeri*)
1 yes (*Phosphatochelys tedfordi*)

23. PA, PA-JU contact (G11Ch10, C12Ch10, F03Ch03)
0 no contact (*Elseya dentata*)
1 contact present (*Podocnemis expansa*)

24. PA, PA-SQ contact (G06Ch15, C12Ch03, G77Ch12)
0 present (*Proganochelys quenstendti*)
1 absent (*Pelusios castanoides*)

25. PA, parietal contacts pterygoid at base of processus trochlearis pterygoidei (G06Ch16)
0 no contact (*Pelusios castanoides*)
1 present, ventral parietal process on lateral side of sulcus palatinopterygoideus (*Bothremys maghrebiana*)

26. PA, PA-PT contact in septum orbitotemporale (G11Ch06)
0 absent (*Peltocephalus dumerilianus*)
1 present and wider (*Bairdemys venezuelensis*)
2 present and narrower (*Podocnemis expansa*)

27. PA, interparietal scale (G11Ch08)
    0 equilateral triangle (*Bauruemys elegans*)
    1 elongate triangle (*Erymnochelys madagascariensis*)
    2 parallel sided (*Bairdemys thalassica*)
    3 slightly elongated heart-like shaped (*Neochelys franzeni*)

28. PA, Interparietal scale, anterior margin (C12Ch12)
    0 anterior to the frontal parietal suture (*Podocnemis expansa*)
    1 posterior to the frontal parietal suture (*Dacquemys palaeomorpha*)

29. PA, parietal scales meeting medially (C15Ch20)
    0 yes (*Podocnemis expansa*)
    1 no (*Peltocephalus dumerilianus*)

30. JU, narrow dorsoventrally (G06C21)
    0 broader (*Bothremys cooki*)
    1 narrower (*Taphrosphys ippolitoi*)

31. JU, jugal retracted from orbital margin (G06Ch20)
    0 enters orbit (*Pelusios castanoides*)
    1 retracted from orbit (*Cearachelys placidoi*)

32. JU-QU contact (G06Ch22, G11Ch09)
    0 no contact (*Bothremys maghrebiana*)
    1 contact present (*Azzabbaremys moragjonesi*)

33. JU-PT contact (F15Ch07)
    0 no contact (*Cordichelys antiqua*)
    1 contact present (*Podocnemis expansa*)

34. JU, exposure in triturating surface (G06Ch23)
    0 no exposure (*Pelusios castanoides*)
    1 exposed on triturating surface (*Bothremys cooki*)

35. SQ, caudal projection (G06Ch24)
    0 lacks projection (*Pelusios castanoides*)
    1 forms distinct process, projecting posteriorly (*Bothremys maghrebiana*)
    2 forms distinct process, projecting posterodorsally (*Hydromedusa tectifera*)

36. SQ, caudoventral vertical flange (G06Ch25, G11Ch12)
    0 absent (*Galianemys emringeri*)
    1 present (*Labrostochelys galkini*)

37. SQ, lateral tubercle (G06Ch26)
    0 absent (*Galianemys emringeri*)
    1 present (*Labrostochelys galkini*)

38. SQ, lateral surface, origin site of the depressor mandibulae muscles (new character; Plate 1)
    0 convex to slightly flat as in *Podocnemis expansa*
    1 strongly concave as in *Elseya dentata*

39. PO, septum orbitotemporale (G06Ch28)

0 postorbital wall closed (*Galianemys emringeri*)
1 postorbital wall at least partially open (*Phosphatochelys tedfordi*)

40. PO, size (G06Ch29, G11Ch13, F03Ch07, G77Ch07)
0 larger or equal to orbit (*Peltocephalus dumerilianus*)
1 smaller than orbit (*Podocnemis expansa*)

41. PM, pinched snout (G11Ch15)
0 no pinched snout, convex or straight rostral outline (*Podocnemis expansa*)
1 pinched snout, concave outline near premaxilla-maxilla contact (*Peltocephalus dumerilianus*)

42. PM, dorsal sulcus (G06Ch33)
0 smooth surface (*Bothremys maghrebiana*)
1 sulcus on dorsal surface, parallel to anterior margin (*Phosphatochelys tedfordi*)

43. PM, protrudes cranially in lateral view beyond apertura narium interna dorsal ridge (G06Ch30)
0 does not protrude or protrudes slightly (*Galianemys emringeri*)
1 protrudes in lateral view (*Bothremys maghrebiana*)

44. PM, midline depression in ventral view (G06Ch31)
0 no depression, shallow or indistinct (*Euraxemys essweini*)
1 depression present (*Bothremys cooki*)

45. PM, shape of the midline depression in ventral view (C15Ch34)
0 narrow depression (*Rosasia soutoi*)
1 wide depression (*Bothremys cooki*)

46. PM, cranial pit on ventral surface (new character; Plate 1)
0 no (*Podocnemis expansa*)
1 yes (*Peltocephalus dumerilianus*)

47. PM, premaxillae reach apertura narium interna (G11Ch14)
0 no (*Podocnemis expansa*)
1 yes (*Pelomedusa subrufa*)

48. PM, One or two accessory ridges on the ventral surface of the premaxilla (C12Ch19)
0 no accessory ridges (*Peltocephalus dumerilianus*)
1 accessory ridges present (*Podocnemis expansa*)

49. PM, foramen prepalatinum (M09Ch14)
0 absent (*Pseudemydura umbrina*)
1 present in premaxilla-maxilar suture (*Elseya dentata*)
2 present in premaxilla only, or in premaxilla-vomer contact (*Araripemys barretoi*)

50. PM, foramen prepalatinum relative to triturating ridge (M09Ch15)
0 on flat surface (*Emydura macquarii*)
1 under triturating surface (*Elseya dentata*)

51. MX, dorsal process onto skull roof (G06Ch41)
0 not developed, mx not constricting PF nor forming part of the rostrodorsal margin of orbit (*Galianemys emringeri*)

1 developed rostrodorsally constricting PF (*Bothremys maghrebiana*)
2 developed rostrodorsally, forming part of the dorsal margin of orbit (*Hydromedusa tectifera*)

52. MX, orbital-narial bar width (G06Ch40)
0 very narrow, less than half the diameter of orbit (*Hydromedusa tectifera*)
1 roughly equal or slightly less than diameter of orbit (*Cearachelys placidoi*)
2 larger than diameter of orbit (*Bothremys maghrebiana*)

53. MX, below orbit (G06Ch37)
0 small, equal or less than orbital diameter (*Galianemys emringeri*)
1 well developed, greater than orbital diameter (*Bothremys maghrebiana*)

54. MX, labial ridge in ventral view (G11Ch19)
0 high (*Elseya dentata*)
1 low (*Phrynops geoffroanus*)

55. MX, posterior lingual ridge near the maxilla-palatine contact (new character; Plate 1)
0 undistinguishable or very shallow ridge (*Euraxemys essweini*)
1 well defined ridge (*Peltocephalus dumerilianus*)

56. MX, triturating surfaces (G06Ch34)
0 narrow as in *Euraxemys essweini*
1 wide as in *Cearachelys placidoi*
2 very wide as in *Bothremys cooki*

57. MX, triturating surfaces shape (G06Ch34, C15Ch37)
0 lingual and labial ridges parallel sided (*Podocnemis expansa*)
1 triangular, wide caudally (*Lapparentemys vilavilensis*)
2 triangular, very wide caudally (*Bothremys maghrebiana*)

58. MX, medial expansion of triturating surface (G11Ch16, C12Ch19)
0 absent (*Bauruemys elegans*)
1 present, forming median maxillary ridge (*Podocnemis expansa*)

59. MX, triturating surface convexity (G11Ch18)
0 absent or shallow (*Podocnemis expansa*)
1 deep (*Bairdemys hartsteini*)

60. MX, accessory ridge (G06Ch36, G11Ch20)
0 absent (*Pelomedusa subrufa*)
1 present (*Elseya dentata*)

61. MX, pits (G06Ch35)
0 absent (*Kurmademys kallamedensis*)
1 present (*Bothremys cooki*)

62. MX, exposure in orbital floor (G06Ch43)
0 broadly exposed (*Galianemys emringeri*)
1 narrowly or not exposed (*Azzabbaremys moragjonesi*)

63. MX, MX-QJ contact (G06Ch38)

0 no contact (*Euraxemys essweini*)
1 contact present (*Galianemys emringeri*)

64. MX, MX-QU contact (G06Ch39)
0 no contact (*Podocnemis expansa*)
1 contact present (*Taphrosphys sulcatus*)

65. VO, vomer (G06Ch45, G11Ch22, C12Ch20, F03Ch08)
0 present (*Phrynops geoffroanus*)
1 absent (*Pelomedusa subrufa*)

66. VO, VO-PM contact (new character; Plate 1)
0 absent (*Phrynops geoffroanus*)
1 present (*Bauruemys elegans*)

67. VO, VO-MX contact (G06Ch44)
0 present (*Proganochelys quenstendti*)
1 absent (*Azabbaremys moragjonesi*)

68. VO, VO-PT contact (J07Ch28)
0 vomer contacts pterygoid (*Phrynops geoffroanus*)
1 vomer does not contact pterygoid (*Elseya dentata*)

69. VO, central bar (G06Ch47)
0 sutured at both ends (*Podocnemis expansa*)
1 sutured only rostrally (*Azzabaremys moragjonesi*)

70. PAL, fossa orbitalis posterior pocket (G06Ch27, G11Ch27)
0 absent (*Podocnemis expansa*)
1 present in septum orbitotemporale (*Bairdemys venezuelensis*)

71. PAL, foramen palatinum posterius (C12Ch27) (ordered)
0 present, only in palatine (*Podocnemis expansa*)
1 present, both in palatine and pterygoid (*Galianemys emringeri*)
2 extremely reduced to absent (*Dacquemys palaeomorpha*)

72. PAL, foramen palatinum posterius (G06Ch48)
0 in floor of orbit (*Proganochelys quenstedti*)
1 behind orbit, in floor of sulcus palatinopterygoideus (*Pelusios castanoides*)

73. PAL, apertura narium interna (BF05Ch11)
0 small to moderate, caudal edge formed only by palatines (*Podocnemis expansa*)
1 large due to reduction of palatines, caudal edge formed by palatines and pterygoids (*Hydromedusa tectifera*)

74. PAL, dorsally arched palate (G06Ch49)
0 absent (*Galianemys emringeri*)
1 present (*Peltocephalus dumerilianus*)

75. PAL, palatine extent in triturating surface (G06Ch50, G11Ch24)
0 narrow or absent (*Elseya dentata*)
1 moderate, but much less than extent of maxilla (*Podocnemis expansa*)

2 large, equal to or slightly less than extent of maxilla (*Bothremys cooki*)

76. PAL, secondary palate (C12Ch28, G11Ch23)
0 absent (*Podocnemis expansa*)
1 present (*Bairdemys venezuelensis*)

77. PAL, secondary palate, medial edges of palatal cleft (G11Ch17)
0 curved (*Bairdemys venezuelensis*)
1 parallel (*Stereogenys cromeri*)

78. PAL, caudal development (G11Ch28)
0 poorly developed as in *Podocnemis expansa*
1 developed, reducing the contact between pterygoids as in *Baidemys venezuelensis*
2 well-developed, reaching the basisphenoid as in *Stereogenys cromeri*

79. PAL, dorsal process reaches frontal (G11Ch26)
0 does not reach frontal (*Podocnemis expansa*)
1 reaches frontal (*Stereogenys cromeri*)

80. PAL, dorsal process contacts parietal in septum orbitotemporale (G11Ch25)
0 does not contact (*Podocnemis expansa*)
1 contacts parietal in septum orbitotemporale (*Stereogenys cromeri*)

81. QU, fossa precolumellaris (G06Ch56, G11Ch30, C12Ch16)
0 very small to absent (*Galianemys emringeri*)
1 present but shallow (*Euraxemys essweini*)
2 deep and well defined (*Pelusios castanoides*)

82. QU, incisura columellae auris (G06Ch52, G11Ch32, C12Ch15, F03Ch24)
0 no posterior bony restriction (*Euraxemys essweini*)
1 eustachian tube separated from stapes by bone or narrow fissure (*Foxemys mechinorum*)
2 eustachian tube and stapes enclosed by bone in the same opening (*Podocnemis expansa*)

83. QU, stapes contained in bony canal (G06Ch53)
0 no (*Euraxemys essweini*)
1 yes (*Bothremys cooki*)

84. QU, ventral process of the sulcus eustachii (G06C54, C15Ch57)
0 no ventral process (*Bothremys cooki*)
1 ventral process present (*Labrostochelys galkini*)

85. QU, trough on closed incisura columellae auris ridge (G06Ch55)
0 absent (*Bothremys maghrebiana*)
1 present (*Galianemys emringeri*)

86. QU, entrance of the antrum postoticum (G06Ch51, G11Ch29, F03Ch22)
0 large to moderate (*Pelusios castanoides*)
1 small (*Podocnemis expansa*)
2 very small and slitlike (*Bairdemys thalassica*)
3 antrum postoticum absent (*Nigeremys gigantea*)

87. QU, shelf below cavum tympani (G06Ch57)

0 absent (*Galianemys emringeri*)
1 lower portion of cavum tympani unusually deep (*Bothremys maghrebiana*)

88. QU, condylus mandibularis position (G06Ch60)
0 caudal to or on BO-BS suture (*Galianemys emringeri*)
1 rostral to BO-BS suture (*Pelusios castanoides*)
2 caudal to condylus occipitalis (*Nigeremys gigantea*)

89. QU, condylus mandibularis shape (C12Ch29)
0 much wider than long, with anterior and posterior edges straight to concave making it shorter at midline (*Bothremys cooki*)
1 slightly wider than long in a 'kidney bean' shape, with anterior edge straight to concave and posterior edge convex (*Podocnemis unifilis*)

90. QU, ventral outline in lateral view (C12Ch17, F03Ch18)
0 smooth, condylus mandibularis very close to the cavum tympani region (*Araripemys barretoi*)
1 ventral projection, condylus mandibularis separated from the cavum tympani region (*Bothremys cooki*)

91. QU, eustachian tube separated from fenestra postotica (G11Ch31)
0 no (*Podocnemis expansa*)
1 yes (*Bairdemys hartsteini*)

92. QU, QU-BO contact (G06Ch59, G11Ch33, C12Ch14, F03Ch13)
0 no contact (*Elseya dentata*)
1 contact present (*Podocnemis expansa*)

93. PT, processus trochlearis pterygoidei (G06Ch70, G11Ch38, F03Ch20)
0 oblique (*Phrynops geoffroanus*)
1 right angle (*Podocnemis expansa*)

94. PT, fossa pterygoidea/cavum pterygoideum (G06Ch68, G06Ch69, G11Ch35, C12Ch26, F03Ch10)
0 absent as in *Euraxemys essweini*
1 well defined as in *Cearachelys placidoi*

95. PT, pterygoid flange (FL06Ch17, G06Ch71, G11Ch37, C12Ch25, F03Ch25, F03Ch21) (ordered)
0 absent (*Proganochelys quenstedti*)
1 very short (*Phrynops geoffroanus*)
2 moderate, does not reach the quadrate ramus of pterygoid (*Brasilemys josai*)
3 well developed, passing the quadrate ramus of the pterygoid and covering the fossa pterygoidea, forming the cavum pterygoideum (*Podocnemis expansa*)

96. PT, anterior opening of cavum pterygoidei (G11Ch36) (ordered)
0 small opening (*Bauruemys elegans*)
1 moderate opening (*Podocnemis expansa*)
2 large opening with foramen cavernosum in roof (*Bairdemys venezuelensis*)

97. PT, foramen caroticum palatinum (G06Ch76)
0 present (*Emydura macquarii*)

1 absent (*Pelusios castanoides*)

98. PT, trigeminal ridge (PT + QU) (G06Ch73)
0 absent (*Galianemys emringeri*)
1 ridge extending posteroventrally from foramen nervi trigemini to condylus mandibularis (*Phosphatochelys tedfordi*)

99. Foramen posterius canalis carotici interni position in relation to BS (G06Ch74, G06Ch75, G11Ch49, C15Ch72, C15Ch73) (ordered)
0 restricted to BS (*Kurmademys kallamedensis*)
1 on BS edge with another bone (*Cearachelys placidoi*)
2 outside BS (*Pelusios castanoides*)
Comment: Characters related to the foramen posterius canalis carotici interni appeared in previous analyses, but they usually unite several features into a single character or leave some variation out of the definition (e.g., characters 74 of Gaffney et al., 2006 and 72 of Cadena, 2015). We decided to distribute this variation (i.e., the position of the foramen posterius canalis carotici interni) into four characters (99 to 102), to better represent all the observed variation, but other versions of these characters can be found elsewhere (G06Ch74; G11Ch49; C15Ch72; J07Ch56).

100. Foramen posterius canalis carotici interni position in relation to fenestra postotica cranial edge (G06Ch74, G06Ch75, G11Ch49, C15Ch72, C15Ch73)
0 rostral to fenestra postotica rostral edge (*Pelomedusa subrufa*)
1 on fenestra postotica rostral edge (*Phrynops geoffroanus*)

101. Foramen posterius canalis carotici interni position in relation to PT ignoring the BS relation (G06Ch74, G06Ch75, G11Ch49, C15Ch72, C15Ch73) (ordered)
0 restricted to PT (*Cearachelys placidoi*)
1 on PT edge with another bone (QU or PR) (*Bothremys cooki*)
2 outside PT (*Hydromedusa tectifera*)

102. Foramen posterius canalis carotici interni on quadrate (G06Ch74, G06Ch75, G11Ch49, C15Ch72, C15Ch73)
0 outside quadrate (*Podocnemis unifilis*)
1 on quadrate (*Bothremys maghrebiana*)

103. SO, roof exposure (G11Ch39)
0 SO not exposed on roof (*Pelusios castanoides*)
1 little exposure of SO on roof (*Elseya dentata*)
2 large exposure of SO on roof (*Pseudemydura umbrina*)

104. SO, crista supraoccipitalis (G06Ch80, C12Ch11, TG09Ch03)
0 not elongated caudal to the foramen magnum (*Pelusios castanoides*)
1 elongated caudal to the foramen magnum (*Podocnemis expansa*)

105. SO, bulbous ending of crista occipitalis (C12Ch11)
0 flat ending (*Podocnemis expansa*)
1 bulbous ending (*Bairdemys thalassica*)

106. SO, horizontal plate along ventral edge of crista supraoccipitalis (G11Ch40)
0 flat or very slim horizontal plate on ventral edge (*Phrynops geoffroanus*)
1 horizontal plate along ventral edge (*Podocnemis unifilis*)

107. SO, supraoccipital lateral margin on otic chamber (G06Ch79)
0 contacts PR and OP but not QU (*Phrynops geoffroanus*)
1 contacts QU (*Bothremys cooki*)

108. SO, supraoccipital lateral margin on roof (J07Ch19)
0 contacts SQ (*Hydromedusa tectifera*)
1 does not contact SQ (*Pelomedusa subrufa*)

109. EX, ventral process (G06Ch86, C15Ch86)
0 absent to very short (*Pelusios castanoides*)
1 present, EX-BS contact avoiding BS-QU contact in ventral view of the skull (*Euraxemys essweini*)

110. EX, condylus occipitalis (G06Ch84, G11Ch41, C12Ch13)
0 basioccipital plus both exoccipitals (*Euraxemys essweini*)
1 exoccipitals only (*Pelusios castanoides*)

111. EX, foramina nervi hypoglossi (G11Ch43)
0 separated (*Podocnemis expansa*)
1 combined and recessed (*Bairdemys thalassica*)

112. EX, foramen jugulare posterius (G06Ch82, G11Ch42)
0 not formed in bone (*Notoemys laticentralis*)
1 closed completely (*Phrynops geoffroanus*)
2 partially openned (*Galianemys emringeri*)

113. EX, dorsal processes of exoccipitals (BF05Ch10, G77Ch17)
0 do not meet medially (*Elseya dentata*)
1 meeting dorsal to the foramen magnum (*Acantochelys radiolata*)

114. EX-QU contact (G06Ch85, C12Ch30)
0 no contact (*Elseya dentata*)
1 narrow contact (*Euraxemys essweini*)
2 extensive contact (*Podocnemis expansa*)

115. BO, length (G06Ch87, G11Ch44, C12Ch21)
0 long, length/width => 0.60 (*Pelusios castanoides*)
1 short, 0.60 > length/width =< 0.25 (*Bothremys cooki*)
2 very short, length/width < 0.25 (*Shweboemys pilgrimi*)

116. BO, basioccipital tubera width in occipital view (G11Ch45)
0 closer to median (*Bauruemys elegans*)
1 farther from median (*Podocnemis expansa*)

117. BO, horizontal occipital shelf (G11Ch46)
0 absent (*Elseya dentata*)
1 present (*Podocnemis expansa*)

118. BO, basioccipital lateral edges (G06Ch89, C12Ch24)
0 does not contact opisthotic (*Galianemys emringeri*)
1 contacts opisthotic (*Pelomedusa subrufa*)

119. PR, ventral exposure, out of cavum pterygoideum (G06Ch94, G11Ch47, C12Ch32, F03Ch11) (ordered)
0 most of PR exposed ventrally (*Phrynops geoffroanus*)
1 about half covered by QU + BS (*Euraxemys essweini*)
2 nearly all covered by QU, BS, PT (*Podocnemis expansa*)

120. PR, foramen stapediotemporale (G06Ch92, F03Ch16)
0 opens dorsally (*Pelusios castanoides*)
1 opens cranially (*Galianemys emringeri*)

121. PR, foramen stapediotemporale/foramen nervi trigemini (G06Ch93)
0 separated by most of prootic (*Pelusios castanoides*)
1 separated by narrow bar of prootic (*Bothremys cooki*)

122. OP, processus interfenestralis covered ventrally (G06Ch99, G11Ch50)
0 visible ventrally (*Emydura macquarii*)
1 coverd by bone (*Podocnemis expansa*)

123. OP, fenestra postotica closed medially (G06Ch100, G11Ch51)
0 open (*Euraxemys essweini*)
1 closed by OP-QU contact (*Podocnemis expansa*)

124. OP, fenestra postotica shape (G06Ch101)
0 more open (*Euraxemys essweini*)
1 small, horizontal slit (*Galianemys emringeri*)

125. OP, opisthotic and exoccipital project ventrally forming a flange over the foramen jugulare posterius (new character; Plate 1)
0 no (*Podocnemis expansa*)
1 yes (*Bairdemys sanchezi*)

126. OP, processus paraoccipitalis in ventral view (G06Ch102, C12Ch22)
0 projects caudally beyond SQ (*Euraxemys essweini*)
1 smaller, rostral to SQ (*Galianemys emringeri*)

127. OP, caudal surface (G06Ch103)
0 smooth edge (*Galianemys emringeri*)
1 thin lateral and horizontal flange present on posterior edge (*Chedighaii hutchisoni*)
2 thin horizontal flange extending medially from the fenestra to the lateral contact with SQ (*Chelodina coillei*)

128. BS, ventral outline (G06Ch106)
0 pentagonal (*Cearachelys placidoi*)
1 triangular (*Euraxemys essweini*)
2 v-shaped (*Nigeremys gigantea*)

129. BS, rostral margin of basisphenoid (J07Ch44) (ordered)
0 very long rostral process in ventral view at least partially dividing the pterygoids (*Chelodina coillei*)
1 short rostral process in ventral view (*Sokatra antitra*)
2 no rostral process in ventral view (*Peltocephalus dumerilianus*)

130. BS, BS-QU contact (G06Ch104, C12Ch23, F03Ch12, G11Ch53, BF05Ch13, G77Ch23)
0 absent (*Euraxemys essweini*)
1 present, wider (*Galianemys emringeri*)
2 present, very narrow (*Bothremys cooki*)

131. BS, processus clinoideus (G06Ch107, C15Ch104)
0 present, with abducens canal (*Pelusios castanoides*)
1 absent, nervi abducens canal is a groove (*Bothremys cooki*)

132. BS, sella turcica/dorsum sella (G06Ch110)
0 deep, well-defined margins (*Taphrosphys sulcatus*)
1 very shallow, low margins (*Bothremys cooki*)

133. SP, splenial (G06Ch113, G11Ch57, F03Ch28)
0 present (*Proganochelys quenstedti*)
1 absent (*Pelusios castanoides*)

134. DEN, widely exposed on lateral surface (G06Ch119)
0 yes, in posterior part of jaw (*Euraxemys essweini*)
1 no, covered by surangular (*Bothremys cooki*)

135. DEN, internal angle between rami (C12Ch34)
0 acute, between 40° and 90° (*Podocnemis unifilis*)
1 obtuse, over 90° (*Phrynops geoffroanus*)

136. DEN, high lingual ridge (G06Ch114)
0 no (*Pelusios castanoides*)
1 yes (*Bothremys cooki*)

137. DEN, U-shaped lingual ridges (G06Ch116)
0 form U-shape, also wedge (*Bothremys cooki*)
1 form V-shape (*Podocnemis unifilis*)

138. DEN, accessory ridges (C12Ch35)
0 absent (*Peltocephalus dumerilianus*)
1 present (*Podocnemis unifilis*)

139. DEN, pits (G06Ch116)
0 no (*Euraxemys essweini*)
1 yes (*Bothremys cooki*)

140. DEN, sutured symphysis (G06C117, G11Ch54, C12Ch33, BF05Ch14, G77Ch24)
0 symphysis fused (*Bothremys cooki*)
1 symphysis sutured (*Euraxemys essweini*)

141. DEN, symphyseal hook (G77, TG09Ch22)
0 no hook on rostral edge (*Proganochelys quenstedti*)
1 hooked rostral edge (*Peltocephalus dumerilianus*)

142. DEN, triturating surfaces (G06Ch118, C15Ch111)
0 narrow as in *Euraxemys essweini*
1 wider as in *Podocnemis unifilis*

2 wide posteriorly as in *Cearachelys placidoi*
3 wide anteriorly as in *Bairdemys venezuelensis*

143. SUR, foramen nervi auriculotemporalis (G06Ch120)
0 absent (*Proganochelys quenstedti*)
1 present (*Podocnemis unifilis*)

144. COR, wide lateral exposure (G06Ch121)
0 no (*Euraxemys essweini*)
1 yes (*Bothremys cooki*)

145. COR, participates on triturating surface (C15Ch114)
0 no or slightly (*Bothremys maghrebiana*)
1 yes, lingual-caudal portion formed by coronoid (*Podocnemis expansa*)
2 yes, caudal portion formed by coronoid (*Stereogenys cromeri*)

146. PRA, fossa meckelii open cranially (G06Ch122)
0 closed by long ang-pra contact and/or splenial (*Euraxemys essweini*)
1 more open, short pra-ang contact (*Bothremys cooki*)

147. ART, processus retroarticularis (G06Ch123, G11Ch55)
0 short, absent (*Pelusios castanoides*)
1 long, posterior (*Bothremys cooki*)
2 long, posteroventral (*Podocnemis unifilis*)

148. ART, foramen chorda tympani enclosed in processus retroarticularis (G11Ch56)
0 no (*Podocnemis expansa*)
1 yes (*Erymnochelys madagascariensis*)

149. VT, cervical articulations (G06C128, G11Ch59, F03Ch46, BF05Ch16)
0 amphicoelous, platycoelous (*Proganochelys quenstedti*)
1 (2((3((4((5))6))7((8) (*Phrynops geoffroanus*)
2 (2))3))4))5))6))7))8) (*Podocnemis expansa*)

150. VT, Cervical vertebrae length (BF05Ch15, G77)
0 smaller than trunk vertebrae (*Podocnemis expansa*)
1 longer than trunk vertebrae (*Chelodina coillei*)

151. VT, Elements of atlantal neural arch (BF05Ch26)
0 dorsally suturally attached (*Emydura macquarii*)
1 dorsally fused (*Hydromedusa tectifera*)

152. VT, Atlantal intercentrum (BF05Ch28)
0 suturally attached to atlantal centrum (*Phrynops geoffroanus*)
1 fused to atlantal centrum (*Hydromedusa tectifera*)

153. VT, Outline of atlantal neural arch in dorsal view (BF05Ch27)
0 convex or subparallel lateral edges (*Pelomedusa subrufa*)
1 lateral edges tapering anteriorly (*Phrynops hilarii*)
2 lateral edges 'wasp-waisted' (*Chelus fimbriatus*)

154. VT, Orientation of atlantal postzygapophyses (BF05Ch25)

0 ventromedial (*Pelusios castanoides*)
1 dorsolateral (*Laganemys tenerensis*)
2 ventral (*Hydromedusa tectifera*)

155. VT, cranial process of neural arch in axis (BF05Ch24)
0 present (*Phyrnops geoffroanus*)
1 absent (*Hydromedusa tectifera*)

156. VT, cervical centrum (G06Ch127)
0 wider than high (*Platychelys oberndorferi*)
1 usually higher than wide or rounder (*Podocnemis expansa*)

157. VT, cervical centra saddle shaped (G11Ch58, C12Ch38, F03Ch47)
0 no heterocoelic vertebrae (*Phrynops geoffroanus*)
1 completely heterocoelic (*Podocnemis unifilis*)
2 wide (*Erymnochelys madagascariensis*)

158. VT, Proportion of condyle of cervical vertebra 8 (BF05Ch22)
0 higher than wide (*Chelodina coillei*)
1 wider than high (*Pelomedusa subrufa*)

159. VT, Outline of condyle of cervical vertebra 8 (BF05Ch23)
0 trapezoidal or sub-oval with shorter ventral side (*Chelus fimbriatus*)
1 subrectangular or suboval (*Chelodina coillei*)
2 trapezoidal or sub-oval with shorter dorsal side (*Hydromedusa tectifera*)
3 kidney-like (*Pelomedusa subrufa*)

160. VT, Ventral keel in cervical vertebrae 8 (BF05Ch19)
0 ventrally developed (*Elseya dentata*)
1 slightly or not developed (*Pelomedusa subrufa*)

161. VT, Shape of the ventral keel on cervical 8 (new character; Plate 1)
0 smooth straight or convex ventral edge (*Elseya dentata*)
1 concave ventral edge (*Podocnemis sextuberculata*)

162. VT, Orientation of dorsal process of neural arch in cervical vertebrae (BF05Ch20)
0 more than 30 degrees with respect to vertebral body (*Pelusios castanoides*)
1 less than 30 degrees with respect to vertebral body (*Araripemys barretoi*)

163. VT, cervical postzygapophyses (G06Ch125, G11Ch60, BF05Ch018)
0 separate (*Proganochelys quenstedti*)
1 some fused (*Podocnemis unifilis*)

164. VT, cervical postzygapophyses (G06Ch126)
0 separated from each other (*Proganochelys quenstedti*)
1 postzygapophyses elevated on neural spine (*Podocnemis unifilis*)

165. VT, Orientation of postzygapophyses of cervical vertebra 8 (BF05Ch21)
0 ventrolateral (*Pelomedusa subrufa*)
1 ventral, horizontal (*Hydromedusa tectifera*)
2 lateral, vertical (*Chelus fimbriatus*)

166. Coracoid shape (C12Ch39)
    0 entirely wide and plate-like (*Proganochelys quenstedti*)
    1 narrow, almost straight longitudinally and slightly wider distally (*Podocnemis unifilis*)
    2 slightly curved longitudinally and much wider distally (*Pelusios castanoides*)

167. Coracoid dorsolongitudinal ridge (C10Ch44, C15Ch132)
    0 absent (*Peltocephalus dumerilianus*)
    1 present (*Podocnemis expansa*)

168. Scapula, angle between acromion and scapular processess (CP15Ch219)
    0 > 70° (*Podocnemis expansa*)
    1 =< 70° (*Bairdemys venezuelensis*)

169. Claws in forefoot (BF05Ch37, G77)
    0 five (*Pelusios castanoides*)
    1 four (*Hydromedusa tectifera*)

170. CAR, nuchal embayment (G06Ch154)
    0 no (*Emydura macquarii*)
    1 yes (*Chedighaii barberi*)

171. CAR, nuchal bone width (G06Ch139, G11Ch62, F03Ch33, BF05Ch30)
    0 greater than length, 2 times or more (*Notoemys laticentralis*)
    1 greater, but less than 2 times, or equal to length (*Phrynops geoffroanus*)
    2 lesser than length (*Araripemys barretoi*)

172. CAR, nuchal proportions (C15Ch135)
    0 cranial width/greatest width < 0.40 (*Turkanemys pattersoni*)
    1 0.70 < cranial width/greatest width >= 0.40 (*Phrynops geoffroanus*)
    2 cranial width/greatest width > 0.70 (*Cordichelys antiqua*)

173. CAR, first pleural scute reaches nuchal (new character; Plate 1)
    0 no (*Cearachelys placidoi*)
    1 yes (*Chedighaii barberii*)

174. CAR, neural number (G06Ch146, G11Ch64, C12Ch42, G77) (ordered)
    0 8 or more (*Araripemys barretoi*)
    1 7 neurals (*Foxemys mechinorum*)
    2 6 (*Kurmademys kallamedensis*)
    3 5 or less (*Phrynops geoffroanus*)
    4 no neurals (*Bairdemys venezuelensis*)

175. CAR, neural series completeness (G06Ch141, G11Ch63, F03Ch38) (ordered)
    0 to suprapygal (*Euraxemys essweini*)
    1 to costals 8 (*Podocnemis unifilis*)
    2 to costals 7 (*Foxemys mechinorum*)
    3 to costals 6 (*Chedighaii barberi*)
    4 to costal 5 & 4 (*Phyrnops geoffroanus*)
    5 neurals discontinuous (*Platemys platicephala*)

176. CAR, neural series pattern (G06Ch145)
    0 irregular (*Platychelys oberndorferi*)

1 regular, most hexagonal, coffin-shaped (*Podocnemis unifilis*)
2 neurals discontinuous (*Araiochelys hirayamen*)

177. CAR, First neural sutured with nuchal (new character; Plate 1)
0 yes (*Phrynops geoffroanus*)
1 no (*Chelodina coillei*)

Comment: this variation is included as state 2 of a character (character 36 of de la Fuente et al., 2017) related to the neural series as a whole. We decided to split this from character 175 because the suture of the first neural to the nuchal is unrelated to the completeness of the whole series.

178. CAR, position of four sided neural (G06Ch144, G11Ch66, C12Ch43)
0 neural one (*Euraxemys essweini*)
1 neural two (*Cearachelys placidoi*)
2 neural three (*Araripemys barretoi*)
3 four-sided neural absent (*Platemys platicephala*)

179. CAR, keeled neurals (G11Ch65)
0 none (*Kenyemys williamsi*)
1 at least some (*Erymnochelys madagascariensis*)

180. CAR, keels or knobs on costals (C15Ch141)
0 no keel or knob on costals (*Podocnemis expansa*)
1 costals with keels or knobs (*Chelus fimbriatus*)

181. CAR, costal one length (G06Ch143)
0 costal 1 shorter or equal to 2 times length of costal 2 (*Euraxemys essweini*)
1 costal 1 two times longer than 2nd costal (*Foxemys mechinorum*)

182. CAR, peripheral 1/costal 1 contact length (G06Ch147)
0 no contact (*Hydromedusa maximiliani*)
1 narrow contact, cranial margin 2x contact (*Foxemys mechinorum*)
2 wide contact, anterior margin less than 2x contact (*Rosasia soutoi*)

183. CAR, peripheral 6 lateral edge (D13Ch100)
0 medially inclined towards cranial edge (*Araripemys barretoi*)
1 almost parallel to cranio-caudal axis (*Phrynops geoffroanus*)
2 medially inclined towards caudal edge (*Cordichelys antiqua*)

184. CAR, Suprapygal sutured with peripheral 10 (F17Ch39)
0 yes (*Chelodina coillei*)
1 no (*Pelusios castanoides*)

185. CAR, suprapygal-peripheral 11 contact (D13Ch105)
0 large (*Phrynops geoffroanus*)
1 small (*Pelomedusa subrufa*)

186. CAR, Proportions of peripheral bones (BF05Ch31)
0 peripheral bones cranial to bridge shorter or equal size than caudal ones (*Emydura macquarii*)
1 peripheral bones cranial to bridge longer than caudal ones (*Phrynops geoffroanus*)

187. CAR, thoracic rib 1 versus 2 (G06C155, C15Ch160)

0 thoracic rib 1 large and separate from thoracic rib 2 (*Notoemys laticentralis*)

1 thoracic rib 1 reduced, almost same size of the medial portion of thoracic rib 2, both separated by an oval elongated space (*Podocnemis expansa*)

2 thoracic rib 1 shorter than the medial exposed portion of thoracic rib 2, very small oval space between them (*Chelus fimbriatus*)

188. CAR, articulation facet on thoracic rib 1 (G06Ch157)
0 absent (*Podocnemis expansa*)
1 present, facet or tubercle on cranial margin of thoracic rib 1 (*Notoemys laticentralis*)

189. CAR, costovertebral tunnel (G06Ch156)
0 large anteriorly and posteriorly only (*Proganochelys quenstedti*)
1 large entire length (*Platychelys oberndorferi*)
2 small (*Chedighaii barberi*)

190. CAR, intumescence from axillary buttress to the rib area on costal 1 (new character; Plate 2)
0 no (*Podocnemis unifilis*)
1 yes (*Bairdemys venezuelensis*)

191. CAR, axillary buttress scar on costals (G06Ch148)
0 contact only peripherals, no contact with costal 1 (*Hydromedusa maximiliani*)
1 contact present but separated from costal 2 suture (*Chedighaii barberi*)
2 contact present and close to costal 2 (*Chelus colombianus*)

192. CAR, axillary buttres contact with costal 1 (TG09Ch25)
0 runs parallel to rib-gomphosis of costal 1 (*Phrynops geoffroanus*)
1 runs in a divergent angle between 15 and 50 degrees to rib-gomphosis (*Elseya dentata*)

193. CAR, axillary buttress extent on ventral surface of peripherals (G06Ch149, G11Ch68, BF05Ch32, C15Ch146)
0 reaches peripheral 2 (*Emydura macquarii*)
1 reaches peripheral 3 (*Pelusios castanoides*)
2 reaches peripheral 4 (*Phrynops hilarii*)
3 reaches peripheral 5 (*Hydromedusa tectifera*)

194. CAR, inguinal buttress on costals (G06Ch150, C12Ch44)
0 short or absent (*Proganochelys quenstedti*)
1 contacts costal 5, extending medially onto it (*Chedighaii barberi*)
2 restricted to costal 4 (*Chelus fimbriatus*)

195. CAR, Inguinal buttress on peripheral (BF05Ch33)
0 6 (*Chelodina coillei*)
1 7 (*Hydromedusa tectifera*)
2 8 (*Phrynops geoffroanus*)

196. CAR, iliac scar reaches costal 7 (G06Ch142, C15Ch142)
0 yes (*Pelusios castanoides*)
1 no (*Elseya dentata*)

197. CAR, iliac scar reaches suprapygal (G06Ch142, C15Ch142)
0 no (*Pelusios castanoides*)
1 yes (*Elseya dentata*)

198. Cervical scute (G06Ch138, G11Ch61, C12Ch41, F03Ch39, BF05Ch36, G77)
0 present (*Emydura macquarii*)
1 absent (*Podocnemis unifilis*)

199. Cervical scute position (BF05Ch36)
0 between marginal scutes (*Phrynops geoffroanus*)
1 behind marginal scutes (*Hydromedusa tectifera*)

200. Cervical Scute Shape (C13*Ch61)
0 width greater than or equal to length (*Hydromedusa tectifera*)
1 width smaller than length (*Phrynops geoffroanus*)

201. CAR, first marginal scute shape (D13Ch104)
0 trapezoidal (*Podocnemis unifilis*)
1 rectangular (*Erymnochelys madagascariensis*)
2 triangular (*Araripemys barretoi*)

202. CAR, marginal scale 1 proportion overlapping the cranial margin/portion of peripheral bone (P13Ch08, C15Ch152)
0 between 30 to 60% (*Phrynops geoffroanus*)
1 less than 30% (*Erymnochelys madagascariensis*)
2 more than 60% (*Cordichelys antiqua*)

203. CAR, vertebral scale width (G06Ch152)
0 equal to or wider than pleural scales (*Pseudemydura umbrina*)
1 narrower than pleural scales (*Foxemys mechinorum*)

204. CAR, vertebral scale 1 reaches cranial margin of carapace (G06Ch153)
0 no, first marginals and or cervical scales (*Podocnemis unifilis*)
1 yes (*Araripemys barretoi*)

205. CAR, relative width of vertebral scute 1 (G77, TG09Ch28)
0 first three vertebral scute equal or sub-equal in width (*Pelusios castanoides*)
1 first vertebral scute wider than second and third (*Phrynops geoffroanus*)

206. Generalised shell shape (TG09Ch36)
0 high domed (*Elseya dentata*)
1 dorso-ventrally flattened (*Phrynops geoffroanus*)

207. CAR, vertebral scale 1 shape of the craniolateral margins (C15Ch156)
0 lateral margins parallel or subparallel to midline axis of carapace (*Elseya dentata*)
1 lateral margins cranially divergent or 'houglass" shaped (*Phrynops geoffroanus*)

208. CAR, second vertebral scute shape (D13Ch103)
0 quadrangular (*Chelodina coillei*)
1 hexagonal (*Hydromedusa tectifera*)
2 trapezoidal (*Araripemys barretoi*)

209. CAR, vertebral scute 2 to 4 longer than wide (D13Ch107)
0 no (*Chelus fimbriatus*)
1 yes (*Chelodina coillei*)

210. CAR, Vertebral scute 5 reaches last neural (new character; Plate 2)
0 no (*Pelusios castanoides*)
1 yes (*Araripemys barretoi*)

211. CAR, Vertebral scute 5 reaches peripheral 10 (C15Ch159)
0 no (*Phrynops geoffroanus*)
1 yes (*Elseya dentata*)

212. CAR, Vertebral scute 5 reaches peripheral 11 (C15ch159)
0 no (*Hydromedusa tectifera*)
1 yes (*Phrynops geoffroanus*)

213. CAR, Vertebral scute 5 reaches Pygal (C15Ch159)
0 no (*Hydromedusa tectifera*)
1 yes (*Elseya dentata*)

214. CAR, pleural scute 4 touches suprapygal (new character; Plate 2)
0 no (*Phrynops geoffroanus*)
1 yes (*Platemys platicephala*)

215. Position of the posterior edge of the plastron related to carapace in ventral view (new character; Plate 2)
0 above C8 or more cranial, peripherals entirely visible (*Araripemys barretoi*)
1 above peripherals 11 (*Podocnemis unifilis*)

216. PLA, cranial lobe reaches carapace edge (G06Ch173)
0 yes (*Cearachelys placidoi*)
1 no (*Foxemys mechinorum*)

217. PLA, cranial lobe length (G06Ch164)
0 long to medium, width over length < = 2.0 (*Podocnemis unifilis*)
1 short, width over length > 2.1 (*Chedighaii barberi*)

218. PLA, cranial lobe (TG09Ch35)
0 wide, squared cranially, lateral margins parallel or sub-parallel (*Platemys platicephala*)
1 narrow, tapered cranially, lateral margins converging cranially (*Emydura macquarii*)

219. PLA, Plastral lobes (BF05Ch34)
0 cranial lobe as long as or longer than caudal one (*Hydromedusa tectifera*)
1 cranial lobe shorter than caudal one (*Pelomedusa subrufa*)

220. PLA, posterior lobe lateral outline (D13Ch118)
0 almost straight lined (*Euraxemys essweini*)
1 concave (*Phrynops geoffroanus*)
2 convex (*Hydromedusa tectifera*)

221. Foramina on suture of hipoplastron and (JB04Ch58)
0 peripheral 7 (*Chelus fimbriatus*)
1 peripheral 8 (*Podocnemis unifilis*)

222. Foramina on suture of hioplastron and (JB04Ch57)

0 peripheral 3 (*Podocnemis unifilis*)
1 peripheral 4 (*Chelus fimbriatus*)

223. PLA, entoplastron shape (G06Ch159)
0 arrow-shaped with caudolateral processes (*Araripemys barretoi*)
1 more trapezoidal (*Podocnemis unifilis*)
2 longer than wider (*Platemys platicephala*)

224. CAR, vertebral scale 1 overlap onto cranial peripherals and nuchal (C15Ch157, C12Ch177)
0 overlapping nuchal and peripherals 1 (*Erymnochelys madagascariensis*)
1 overlapping nuchal, peripherals 1 and 2 (*Acantochelys radiolata*)
2 overlaping only nuchals (*Bairdemys healeyorum*)

225. PLA, mesoplastra (G06Ch158, G11Ch71, C12Ch45, F03Ch40, BF05Ch35)
0 present, wider than long (*Notoemys laticentralis*)
1 present, rounder and lateral (*Podocnemis expansa*)
2 absent (*Chelus fimbriatus*)

226. PLA, fontanella between hyo and hypoplastra (C15Ch171)
0 yes (*Notoemys laticentralis*)
1 no (*Podocnemis expansa*)

227. PLA, pectorals on entoplastron (G11Ch74, F03Ch36, F03Ch41)
0 no (*Euraxemys essweini*)
1 yes (*Foxemys mechinorum*)

228. PLA, pectoral on epiplastron (G06Ch166, G11Ch74, F03Ch36, F03Ch41)
0 no, far behind epiplastron (*Cearachelys placidoi*)
1 on epiplastron, on epi-hyoplastron suture (*Foxemys mechinorum*)

229. PLA, pectoral on mesoplastron (G06Ch167, G11Ch72, F03Ch34)
0 yes (*Cearachelys placidoi*)
1 anterior to mesoplastron (*Kurmademys kallamedensis*)

230. PLA, gular scute (G06Ch170, C12Ch46, G77, C15Ch180)
0 small, humerals contact in midline (*Cearachelys placidoi*)
1 large, separating extragulars and humerals (*Taphrosphrys sulcatus*)

231. PLA, extragulars size (new character; Plate 2)
0 large, almost the same size as gular (*Chelus fimbriatus*)
1 smaller than gular (*Pelomedusa subrufa*)
2 extragulars absent (*Araripemys barretoi*)

232. PLA, entoplastron caudal end reaching the axillary notch level of plastron (C13Ch179, C15Ch166)
0 not reaching (*Phrynops geoffroanus*)
1 caudal end reaches axillary notch (*Foxemys mechinorum*)

233. PLA, extragulars in comparison to gular (C15Ch180)
0 separated by intergular (*Peltocephalus dumerilianus*)
1 larger than intergular, in contact in midline caudally (*Erymnochelys madagascariensis*)

234. PLA, gular scales reach entoplastron (D13Ch120)
 0 yes (*Erymnochelys madagascariensis*)
 1 no (*Podocnemis unifilis*)

235. PLA, humeral scale (new character; Plate 2)
 0 longest length humeral < longest length pectoral (*Elseya dentata*)
 1 longest length humeral = > longest length pectoral (*Pelusios castanoides*)

236. PLA, abdominal scale length (G06Ch172)
 0 midline length abdominal = > midline length anal (*Podocnemis unifilis*)
 1 midline length abdominal < midline length anal (*Taphrosphys sulcatus*)

237. PLA, strong constriction on femural-anal scute on xiphiplastra (new character; Plate 3)
 0 no, smooth edge (*Elseya dentata*)
 1 yes (*Phrynops hilarii*)

238. PEL, pelvis sutured to shell (G06Ch133)
 0 no (*Proganochelys quenstedti*)
 1 yes (*Pelusios castanoides*)

239. PLA, pubic scar shape (new character; Plate 2)
 0 wider as in *Phrynops hilarii* (largest width/length >0.40)
 1 narrower as in *Podocnemis unifilis* (largest width/length <0.40)

240. PLA, ischial scar shape (G06Ch168)
 0 large and linear or V-shaped (*Podocnemis unifilis*)
 1 small and equidimensional (*Taphrosphys sulcatus*)

241. PLA, ischial scar position related to the lateral edges of xiphiplastron (new character; Plate 3)
 0 far from the caudal and lateral edges of xiphiplastron (*Peltocephalus dumerilianus*)
 1 closer to the caudal and lateral edges of xiphiplastron (*Chelus fimbriatus*)

242. PLA, cranial margin width of gulars (C15Ch182, C12Ch46, P13Ch14)
 0 much narrower than the cranial margin of intergular (*Taphrosphys sulcatus*)
 1 almost the same width as the cranial margin of intergular (*Podocnemis expansa*)
 2 wider than the cranial margin of intergular (*Papoulemys laurenti*)
 3 much wider than the cranial margin of intergular (*Erymnochelys madagascariensis*)

243. PLA, ischial scar position related to the anal notch of xiphiplastron (C15Ch177)
 0 the most caudal tip of the scar reaches the level of the anal notch (*Podocnemis expansa*)
 1 the most caudal tip is located cranially to the anal notch level (*Foxemys mechinorum*)

244. PLA, gular position (new character; Plate 3)
 0 gular reaches cranial margin of plastron (*Podocnemis expansa*)
 1 gular retracted from cranial margin of plastron (*Chelodina colliei*)

245. PLA, shape of caudal process of xiphiplastron (C08Ch04)
 0 short and wide (*Podocnemis expansa*)
 1 long and narrow (*Chelus fimbriatus*)

2.5. List of common synapomorphies

Synapomorphies common to 36 trees.

Node numbers refer to nodes in consensus [Fig. S1]; character numbers in TNT output system, i.e. starting from 0 instead of 1.

Proganochelys_quenstedti :
All trees:
No autapomorphies:

Platychelys_oberndorferi :
All trees:
Char. 179: 0 --> 1
Char. 193: 0 --> 1
Char. 209: 0 --> 1

Notoemys_laticentralis :
All trees:
Char. 192: 0 --> 1
Char. 194: 1 --> 2
Char. 200: 1 --> 0
Char. 206: 0 --> 1

Bonapartemys_bajobarrealis :
All trees:
Char. 191: 0 --> 1
Char. 192: 2 --> 1
Char. 194: 1 --> 2
Char. 208: 0 --> 1
Char. 212: 1 --> 0
Char. 224: 0 --> 1

Prochelidella_cerrobarcinae :
All trees:
Char. 182: 1 --> 2
Char. 199: 0 --> 1
Char. 209: 0 --> 1
Char. 218: 0 --> 1

Pseudemydura_umbrina :
All trees:
Char. 9: 1 --> 0
Char. 17: 1 --> 0
Char. 81: 2 --> 0
Char. 102: 1 --> 2
Char. 107: 1 --> 0
Char. 111: 1 --> 2
Char. 169: 0 --> 1
Char. 172: 0 --> 1
Char. 183: 1 --> 0
Char. 202: 1 --> 0
Char. 207: 0 --> 1
Char. 216: 0 --> 1
Char. 226: 0 --> 1
Char. 227: 0 --> 1
Char. 229: 0 --> 1
Char. 235: 0 --> 1
Char. 236: 0 --> 1

Elseya_dentata :
All trees:
Char. 47: 0 --> 1
Char. 59: 0 --> 1
Char. 80: 0 --> 1
Char. 103: 0 --> 1
Char. 128: 1 --> 2
Char. 191: 0 --> 1
Char. 192: 0 --> 1
Char. 208: 0 --> 1

Emydura_macquarii :
All trees:
Char. 70: 1 --> 0
Char. 80: 0 --> 1
Char. 189: 0 --> 1
Char. 191: 0 --> 1
Some trees:
Char. 221: 1 --> 0

Myuchelys_latisternum :
All trees:
Char. 3: 0 --> 1
Char. 8: 0 --> 1
Char. 70: 1 --> 0
Char. 129: 0 --> 2
Char. 207: 0 --> 1
Char. 218: 1 --> 0

Acanthochelys_radiolata :
All trees:
Char. 5: 0 --> 1
Char. 98: 2 --> 1
Char. 164: 0 --> 2
Char. 223: 0 --> 1

Platemys_platicephala :
All trees:
Char. 9: 1 --> 0
Char. 18: 2 --> 1
Char. 19: 3 --> 0
Char. 34: 2 --> 0
Char. 46: 0 --> 1
Char. 50: 2 --> 0
Char. 65: 0 --> 1
Char. 66: 0 --> 1
Char. 153: 0 --> 3
Char. 157: 0 --> 1
Char. 158: 1 --> 2
Char. 170: 1 --> 2
Char. 183: 1 --> 0
Char. 204: 1 --> 0
Char. 213: 0 --> 1

Phrynops_hilarii :
All trees:
Char. 230: 1 --> 0

Phrynops_geoffroanus :
All trees:
Char. 192: 2 --> 1

Mesoclemmys_nasuta :
All trees:
Char. 87: 1 --> 0
Char. 128: 1 --> 2
Char. 174: 3 --> 4
Char. 176: 0 --> 1
Char. 177: 0 --> 3
Char. 181: 1 --> 2

Chelodina_colliei :
All trees:
Char. 38: 0 --> 1
Char. 61: 0 --> 1
Char. 67: 0 --> 1
Char. 98: 2 --> 1
Char. 117: 1 --> 0
Char. 172: 0 --> 1
Char. 174: 2 --> 5
Char. 176: 0 --> 1
Char. 180: 1 --> 0
Char. 183: 1 --> 0

Char. 194: 1 --> 0
Char. 200: 1 --> 0
Char. 208: 0 --> 1
Char. 211: 1 --> 0
Char. 212: 1 --> 0
Char. 213: 0 --> 1
Char. 214: 1 --> 0
Char. 223: 0 --> 2
Char. 226: 0 --> 1
Char. 227: 0 --> 1
Char. 229: 0 --> 1
Char. 234: 1 --> 0
Char. 243: 0 --> 1

Chelus_colombianus :
All trees:
Char. 190: 1 --> 2
Char. 196: 1 --> 0
Char. 243: 0 --> 1

Chelus_fimbriatus :
All trees:
Char. 226: 0 --> 1
Char. 233: 1 --> 0

Hydromedusa_tectifera :
All trees:
Char. 172: 0 --> 1
Char. 204: 1 --> 0
Char. 211: 1 --> 0
Char. 212: 1 --> 0

Hydromedusa_maximilliani
:
All trees:
Char. 206: 1 --> 0
Char. 236: 0 --> 1

Yaminuechelys_maior :
All trees:
Char. 158: 2 --> 3
Char. 236: 0 --> 1

Yaminuechelys_gasparinii
:
All trees:
Char. 219: 1 --> 0
Char. 244: 1 --> 0

Pelusios_castaneus :
All trees:
Char. 43: 0 --> 1
Char. 103: 0 --> 1

Pelusios_castanoides :
All trees:
Char. 46: 1 --> 0
Char. 173: 1 --> 2

Pelomedusa_subrufa :
All trees:
Char. 139: 0 --> 1
Char. 170: 1 --> 2
Char. 180: 0 --> 1
Char. 206: 0 --> 1
Char. 207: 0 --> 1

Araripemys_barretoi :
All trees:
Char. 18: 1 --> 3
Char. 64: 0 --> 1
Char. 140: 1 --> 0
Char. 143: 0 --> 1
Char. 190: 1 --> 0
Char. 206: 0 --> 1
Char. 207: 0 --> 2
Char. 209: 0 --> 1
Char. 224: 1 --> 2

Laganemys_tenerensis :
All trees:
Char. 109: 0 --> 1
Char. 142: 1 --> 0
Char. 175: 1 --> 0
Char. 183: 1 --> 0
Char. 193: 0 --> 1

Euraxemys_essweini :
All trees:
Char. 46: 1 --> 0
Char. 100: 1 --> 0

Dirqadim_schaefferi :
All trees:
Char. 17: 2 --> 1
Char. 48: 2 --> 0

Sokatra_antitra :
All trees:
Char. 13: 1 --> 0
Char. 39: 0 --> 1
Char. 59: 0 --> 1
Char. 88: 1 --> 0
Char. 111: 1 --> 2

Atolchelys_lepida :
All trees:
Char. 53: 0 --> 1
Char. 66: 0 --> 1
Char. 70: 1 --> 0
Char. 216: 0 --> 1
Some trees:
Char. 100: 1 --> 0

Cearachelys_placidoi :
All trees:
Char. 69: 1 --> 0
Char. 81: 1 --> 02
Char. 82: 1 --> 0

Galianemys_emringeri :
All trees:
No autapomorphies:

Galianemys_whitei :
All trees:
No autapomorphies:

Kurmademys_kallamedensi
s :
All trees:
Char. 98: 1 --> 0
Char. 100: 01 --> 2
Some trees:
Char. 24: 0 --> 1
Char. 48: 2 --> 0
Char. 84: 0 --> 1
Char. 173: 1 --> 2
Char. 174: 2 --> 3
Char. 181: 1 --> 2
Char. 219: 0 --> 2
Char. 227: 0 --> 1

Sankuchemys_sethnai :
All trees:
Char. 59: 0 --> 1
Some trees:
Char. 66: 0 --> 1

Kinkonychelys_rogersi :
All trees:
No autapomorphies:

Foxemys_mechinorum :

All trees:
Char. 48: 2 --> 0
Char. 223: 0 --> 1

Foxemys_trabanti :
All trees:
No autapomorphies:

Polysternon_provinciale :
All trees:
Char. 87: 0 --> 1
Char. 110: 0 --> 1
Char. 169: 0 --> 1
Char. 216: 1 --> 0
Char. 219: 0 --> 2
Char. 228: 0 --> 1

Araiochelys_hirayamen :
All trees:
Char. 4: 1 --> 0
Char. 9: 2 --> 1
Char. 102: 0 --> 1
Char. 141: 2 --> 0
Char. 173: 1 --> 2
Char. 175: 1 --> 2
Char. 228: 0 --> 1

Zolhafah_bella :
All trees:
Char. 32: 1 --> 0
Char. 44: 0 --> 1
Char. 57: 0 --> 1
Char. 106: 1 --> 0
Char. 114: 1 --> 2

Rosasia_soutoi :
All trees:
Char. 56: 1 --> 2
Char. 181: 1 --> 0
Char. 206: 1 --> 0
Char. 226: 1 --> 0

Bothremys_maghrebiana :
All trees:
Char. 32: 1 --> 0
Char. 110: 0 --> 1

Bothremys_cooki :
All trees:
No autapomorphies:

Bothremys_arabicus :
All trees:
No autapomorphies:

Bothremys_kellyi :
All trees:
Char. 32: 1 --> 0
Char. 98: 2 --> 1

Chedighaii_hutchisoni :
All trees:
No autapomorphies:

Chedighaii_barberi :
All trees:
Char. 55: 2 --> 1

Taphrosphys_sulcatus :
All trees:
Char. 102: 0 --> 1
Char. 114: 1 --> 0

Taphrosphys_ippolitoi :
All trees:
No autapomorphies:

Taphrosphys_congolensis
:
All trees:
No autapomorphies:

Acleistochelys_maliensis
:
All trees:
Char. 33: 0 --> 1
Char. 59: 0 --> 1
Char. 60: 0 --> 1
Char. 77: 01 --> 2
Char. 88: 0 --> 1
Char. 94: 2 --> 0
Char. 109: 1 --> 0

Azabbaremys_moragjonesi
:
All trees:
Char. 43: 1 --> 0
Char. 45: 0 --> 1
Char. 85: 0 --> 3

Labrostochelys_galkini :
All trees:
Char. 0: 0 --> 1
Char. 6: 0 --> 1
Char. 44: 0 --> 1
Char. 51: 1 --> 2
Char. 73: 1 --> 0
Char. 85: 0 --> 1
Char. 100: 1 --> 2

Phosphatochelys_tedfordi
:
All trees:
Char. 24: 0 --> 1

Ummulisani_rutgersensis
:
All trees:
Char. 85: 0 --> 3
Char. 100: 1 --> 2
Char. 102: 0 --> 1

Rhothonemys_brinkmani
:
All trees:
No autapomorphies:

Arenila_krebsi :
All trees:
No autapomorphies:

Nigeremys_gigantea :
All trees:
Char. 70: 1 --> 0

Brasilemys_josai :
All trees:
Char. 80: 2 --> 1
Char. 81: 2 --> 0
Char. 108: 0 --> 1
Char. 111: 1 --> 2
Char. 174: 2 --> 1

Hamadachelys_escuilliei :
All trees:
Char. 147: 0 --> 1

Portezueloemys_patagonica
:
All trees:
Char. 27: 0 --> 1
Char. 173: 1 --> 2
Char. 207: 0 --> 2

Bauruemys_elegans :
All trees:
Char. 157: 0 --> 1
Char. 173: 1 --> 2
Char. 177: 0 --> 1

Cambaremys_langertoni :
All trees:
Char. 170: 1 --> 2

Peiropemys_mezzalirai :
All trees:
Char. 54: 1 --> 0

Lapparentemys_vilavilensis :
All trees:
No autapomorphies:

Pricemys_caiera :
All trees:
Char. 26: 0 --> 2
Char. 28: 0 --> 1

Cerrejonemys_wayuunaiki :
All trees:
Char. 64: 0 --> 1
Char. 134: 0 --> 1

Podocnemis_vogli :
All trees:
Char. 174: 2 --> 1
Char. 208: 0 --> 1
Some trees:
Char. 95: 1 --> 0
Char. 236: 0 --> 1

Podocnemis_lewyana :
Some trees:
Char. 219: 2 --> 0

Podocnemis_unifilis :
Some trees:
Char. 25: 2 --> 0
Char. 171: 1 --> 0
Char. 178: 0 --> 1

Podocnemis_erythrocephala :
All trees:
Char. 170: 1 --> 2
Char. 181: 2 --> 1
Char. 207: 1 --> 2
Some trees:
Char. 95: 0 --> 1
Char. 160: 0 --> 1
Char. 171: 1 --> 0
Char. 219: 0 --> 2

Podocnemis_expansa :
All trees:
Char. 77: 0 --> 1
Char. 207: 1 --> 2
Some trees:
Char. 80: 2 --> 1
Char. 221: 0 --> 1

Podocnemis_sextuberculata :
All trees:
No autapomorphies:

Podocnemis_bassleri :
All trees:
Char. 51: 1 --> 0
Char. 52: 0 --> 1

Caninemys_tridentata :
All trees:
Char. 25: 0 --> 2
Char. 26: 0 --> 1
Char. 28: 0 --> 1
Char. 52: 0 --> 1
Char. 73: 0 --> 1
Char. 80: 2 --> 0
Char. 85: 1 --> 2
Char. 87: 1 --> 0

Stupendemys_geographicus :
All trees:
No autapomorphies:

Dacquemys_paleomorpha :
All trees:
Char. 137: 0 --> 1
Some trees:
Char. 45: 0 --> 1

UCMP_42008 :
All trees:
Char. 1: 0 --> 1
Char. 13: 1 --> 0
Char. 31: 0 --> 1
Char. 86: 0 --> 1
Char. 107: 1 --> 0

Carbonemys_cofrinii :
All trees:
Char. 87: 1 --> 0

Peltocephalus_dumerilianus :
All trees:
Char. 13: 1 --> 0
Char. 31: 0 --> 1
Char. 40: 0 --> 1
Char. 48: 2 --> 0
Char. 105: 1 --> 0
Char. 207: 0 --> 2
Char. 227: 1 --> 0
Some trees:
Char. 59: 0 --> 1

Kenyemys_williamsi :
All trees:
Char. 169: 0 --> 1
Char. 172: 0 --> 1
Char. 178: 0 --> 1
Char. 200: 1 --> 0
Char. 210: 0 --> 1

Turkanemys_pattersoni :
All trees:
Char. 40: 0 --> 1
Char. 137: 0 --> 1
Char. 171: 1 --> 0
Char. 181: 2 --> 1
Char. 189: 0 --> 1
Char. 201: 1 --> 0

Erymnochelys_madagascariensis :
All trees:
Char. 26: 0 --> 1
Char. 105: 1 --> 0
Char. 241: 1 --> 2

Neochelys_fajumensis :
All trees:
Char. 80: 2 --> 1
Char. 185: 0 --> 1
Char. 201: 1 --> 0

Neochelys_arenarum :
All trees:
Char. 40: 0 --> 1
Char. 230: 0 --> 1

Neochelys_franzeni :
All trees:
Char. 228: 1 --> 0

Papoulemys_laurenti :
All trees:
Char. 219: 2 --> 0

Mogharemys_blackenhorni
:
All trees:
Char. 53: 0 --> 1
Char. 117: 1 --> 0

Brontochelys_gaffneyi :
All trees:
No autapomorphies:

Lemurchelys_diasphax :
All trees:
Char. 42: 0 --> 1
Char. 56: 1 --> 2
Char. 110: 1 --> 0

Shweboemys_pilgrimi :
All trees:
No autapomorphies:

Stereogenys_cromeri :
All trees:
Char. 129: 1 --> 2

Cordichelys_antiqua :
All trees:
Char. 8: 0 --> 1
Char. 32: 1 --> 0
Char. 76: 0 --> 1
Char. 169: 0 --> 1
Char. 181: 2 --> 1
Char. 182: 1 --> 2
Char. 192: 1 --> 0
Char. 205: 0 --> 1
Char. 233: 1 --> 0

Latentemys_plowdeni :
All trees:
No autapomorphies:

Bairdemys_thalassica :
All trees:
Char. 102: 0 --> 1

Bairdemys_healeyorum :
All trees:
Char. 180: 0 --> 1
Char. 241: 1 --> 0

Bairdemys_venezuelensis
:
All trees:
No autapomorphies:

Bairdemys_hartsteini :
All trees:
No autapomorphies:

Bairdemys_sanchezi :
All trees:
Char. 17: 1 --> 2
Char. 18: 0 --> 1
Char. 26: 2 --> 1
Char. 58: 1 --> 0
Char. 110: 1 --> 0

Bairdemys_winklerae :
All trees:
Char. 18: 0 --> 1

Node 102 :
All trees:
Char. 187: 0 --> 1
Char. 205: 0 --> 1
Char. 217: 1 --> 0
Char. 219: 0 --> 1
Char. 225: 1 --> 0

Node 103 :
All trees:
No synapomorphies

Node 104 :
All trees:
Char. 169: 0 --> 1
Char. 180: 1 --> 0
Char. 185: 1 --> 0
Char. 219: 2 --> 1

Node 105 :
All trees:
Char. 173: 1 --> 0
Char. 224: 2 --> 0
Char. 238: 0 --> 1
Char. 244: 0 --> 1

Node 106 :
All trees:
Char. 72: 0 --> 1
Char. 107: 1 --> 0
Char. 157: 0 --> 1
Char. 174: 2 --> 1

Node 107 :
All trees:
Char. 9: 1 --> 2
Char. 19: 3 --> 2
Char. 129: 0 --> 2
Char. 150: 0 --> 1
Char. 168: 0 --> 1

Node 108 :
All trees:
Char. 0: 0 --> 2
Char. 42: 0 --> 1
Char. 128: 1 --> 0
Char. 149: 0 --> 1
Char. 152: 0 --> 2
Char. 161: 0 --> 1
Char. 162: 0 --> 1
Char. 173: 4 --> 12
Char. 199: 1 --> 0

Node 109 :
All trees:
Char. 18: 0 --> 2
Char. 19: 0 --> 3
Char. 53: 0 --> 1
Char. 112: 0 --> 1
Char. 126: 0 --> 2
Char. 139: 0 --> 1
Char. 180: 0 --> 1
Char. 185: 0 --> 1
Char. 205: 0 --> 1
Char. 206: 0 --> 1
Char. 218: 1 --> 0

Node 110 :
All trees:
Char. 88: 0 --> 1
Char. 98: 1 --> 2
Char. 140: 1 --> 0
Char. 217: 1 --> 0

Node 111 :
All trees:
Char. 67: 1 --> 0
Char. 125: 0 --> 1
Char. 164: 1 --> 0

Node 112 :
All trees:
Char. 10: 0 --> 1
Char. 13: 12 --> 3
Char. 14: 1 --> 0
Char. 34: 0 --> 2
Char. 37: 0 --> 1
Char. 39: 0 --> 1
Char. 48: 2 --> 1
Char. 50: 0 --> 2
Char. 65: 1 --> 0
Char. 159: 1 --> 0
Char. 173: 01 --> 4
Char. 219: 0 --> 2

Node 113 :
All trees:
Char. 111: 0 --> 1
Char. 163: 0 --> 1
Char. 170: 0 --> 1
Char. 186: 0 --> 1
Char. 202: 0 --> 1
Char. 214: 0 --> 1
Char. 218: 0 --> 1
Char. 233: 0 --> 1

Node 114 :
All trees:
Char. 43: 0 --> 1
Char. 121: 0 --> 1
Char. 197: 0 --> 1

Node 115 :
All trees:
Char. 101: 0 --> 1
Char. 194: 1 --> 2

Node 116 :
All trees:
Char. 99: 0 --> 1
Char. 196: 1 --> 0
Char. 210: 1 --> 0
Char. 240: 1 --> 0

Node 117 :
All trees:
Char. 219: 2 --> 1
Char. 236: 0 --> 1

Node 118 :
All trees:
Char. 4: 0 --> 1
Char. 152: 0 --> 1
Char. 173: 4 --> 3

Node 119 :
All trees:
Char. 175: 1 --> 0
Char. 178: 0 --> 1
Char. 179: 0 --> 1
Char. 191: 0 --> 1
Char. 193: 0 --> 2
Char. 219: 2 --> 1
Char. 244: 0 --> 1

Node 120 :
All trees:
Char. 151: 0 --> 1
Char. 154: 0 --> 1
Char. 164: 0 --> 1
Char. 175: 1 --> 0
Char. 177: 0 --> 1
Char. 190: 1 --> 0
Char. 192: 2 --> 3
Char. 198: 0 --> 1
Char. 205: 1 --> 0
Char. 207: 0 --> 1
Char. 242: 0 --> 1

Node 121 :
All trees:
Char. 170: 1 --> 0
Char. 207: 0 --> 1
Char. 225: 1 --> 0

Node 122 :
All trees:
Char. 59: 0 --> 1
Char. 224: 1 --> 0

Node 123 :
All trees:
Char. 9: 1 --> 0
Char. 18: 2 --> 3
Char. 19: 0 --> 2
Char. 48: 2 --> 0
Char. 64: 0 --> 1
Char. 109: 0 --> 1
Char. 134: 0 --> 1
Char. 167: 0 --> 1
Char. 190: 1 --> 0
Char. 219: 0 --> 2

Node 124 :
All trees:
Char. 18: 1 --> 2
Char. 96: 0 --> 1
Char. 164: 1 --> 0

Node 125 :
All trees:
Char. 0: 0 --> 1
Char. 2: 0 --> 1
Char. 5: 0 --> 1
Char. 17: 1 --> 2
Char. 18: 0 --> 1
Char. 46: 0 --> 1
Char. 80: 0 --> 2
Char. 88: 0 --> 1
Char. 132: 0 --> 1
Char. 148: 1 --> 2
Char. 192: 0 --> 1
Char. 197: 0 --> 1
Char. 210: 1 --> 0

Node 126 :
All trees:
Char. 0: 1 --> 2
Char. 9: 1 --> 2
Char. 54: 1 --> 0
Char. 128: 1 --> 0
Char. 149: 0 --> 1
Char. 169: 0 --> 1
Char. 171: 1 --> 2
Char. 177: 0 --> 2
Char. 182: 1 --> 0
Char. 200: 1 --> 2
Char. 203: 0 --> 1
Char. 214: 1 --> 0
Char. 222: 1 --> 0
Char. 225: 1 --> 0
Char. 230: 1 --> 2

Node 127 :
All trees:
Char. 111: 1 --> 2
Char. 139: 0 --> 1
Char. 235: 0 --> 1

Node 128 :
All trees:
Char. 59: 0 --> 1
Char. 80: 2 --> 1
Char. 100: 2 --> 1
Char. 108: 0 --> 1
Char. 113: 0 --> 1
Char. 118: 0 --> 1
Char. 121: 0 --> 1
Char. 128: 1 --> 2

Node 129 :
All trees:
Char. 113: 0 --> 1
Char. 118: 0 --> 1
Char. 121: 0 --> 1

Node 130 :
All trees:
Char. 92: 0 --> 1
Char. 94: 1 --> 2
Char. 100: 2 --> 01

Node 131 :
All trees:
Char. 30: 0 --> 1
Char. 111: 1 --> 2
Char. 122: 1 --> 0
Char. 123: 0 --> 1

Node 132 :
All trees:
Char. 80: 2 --> 0
Char. 119: 0 --> 1

Node 133 :
All trees:
Char. 87: 1 --> 0
Some trees:
Char. 109: 0 --> 1

Node 134 :
All trees:
Char. 19: 0 --> 1
Char. 113: 1 --> 2
Some trees:
Char. 69: 0 --> 1
Char. 81: 2 --> 1
Char. 82: 0 --> 1
Char. 88: 1 --> 0
Char. 89: 0 --> 1
Char. 135: 0 --> 1
Char. 141: 0 --> 2
Char. 206: 0 --> 1

Node 135 :
All trees:
Char. 56: 0 --> 1
Char. 74: 0 --> 1
Char. 91: 0 --> 1
Char. 118: 1 --> 2
Char. 128: 1 --> 2

Node 136 :
All trees:
Char. 84: 0 --> 1
Char. 109: 1 --> 0

Node 137 :
All trees:
Char. 44: 0 --> 1
Char. 82: 1 --> 0
Char. 111: 1 --> 2
Char. 227: 0 --> 1
Char. 231: 0 --> 1

Node 138 :
All trees:
Char. 4: 0 --> 1
Char. 12: 0 --> 1
Char. 143: 0 --> 1
Char. 215: 0 --> 1

Node 139 :
All trees:
Char. 86: 0 --> 1
Char. 115: 0 --> 1
Char. 120: 0 --> 1
Char. 133: 0 --> 1
Char. 145: 0 --> 1
Char. 216: 0 --> 1

Node 140 :
All trees:
Char. 1: 0 --> 1
Char. 6: 0 --> 1
Char. 42: 0 --> 1
Char. 51: 0 --> 1
Char. 98: 1 --> 2

Node 141 :
All trees:
Char. 93: 1 --> 0
Char. 100: 0 --> 1
Char. 101: 0 --> 1

Node 142 :
All trees:
Char. 9: 1 --> 2
Char. 33: 0 --> 1
Char. 60: 0 --> 1
Char. 74: 1 --> 2
Char. 169: 0 --> 1

Node 143 :
All trees:
Char. 50: 0 --> 1

Node 144 :
All trees:
Char. 51: 1 --> 2

Node 145 :
All trees:
Char. 52: 0 --> 1
Char. 56: 1 --> 2
Char. 92: 1 --> 0
Char. 126: 0 --> 1
Char. 129: 1 --> 2

Node 146 :
All trees:
Char. 44: 0 --> 1
Char. 114: 1 --> 2

Node 147 :
All trees:
Char. 33: 1 --> 0
Char. 60: 1 --> 0

Node 148 :
All trees:
Char. 131: 0 --> 1

Node 149 :
All trees:
Char. 98: 2 --> 1

Node 150 :

All trees:
Char. 41: 0 --> 1
Char. 97: 0 --> 1
Char. 127: 1 --> 0

Node 151 :
All trees:
Char. 15: 1 --> 0
Char. 35: 0 --> 1
Char. 36: 0 --> 1
Char. 83: 0 --> 1
Char. 93: 1 --> 0
Char. 129: 2 --> 1

Node 152 :
All trees:
Char. 98: 1 --> 2
Char. 100: 0 --> 1
Char. 101: 0 --> 1
Char. 106: 1 --> 0

Node 153 :
All trees:
Char. 38: 0 --> 1
Char. 42: 0 --> 1
Char. 46: 0 --> 1
Char. 66: 0 --> 1

Node 154 :
All trees:
Char. 51: 0 --> 1
Char. 55: 1 --> 0
Char. 56: 1 --> 0
Char. 73: 0 --> 1
Char. 74: 1 --> 0
Char. 92: 1 --> 0
Char. 129: 1 --> 2

Node 155 :
All trees:
Char. 7: 0 --> 1

Node 156 :
All trees:
Char. 21: 0 --> 1
Char. 51: 1 --> 0
Char. 52: 0 --> 1

Node 157 :
All trees:
Char. 54: 1 --> 0
Char. 85: 0 --> 3
Char. 87: 0 --> 2

Node 158 :
All trees:
Char. 85: 0 --> 1
Char. 155: 1 --> 0

Node 159 :
All trees:
Char. 116: 0 --> 1

Node 160 :
All trees:
Char. 17: 2 --> 1
Char. 94: 2 --> 3

Node 161 :
All trees:
Char. 238: 0 --> 1

Node 162 :
All trees:
Char. 227: 0 --> 1

Node 163 :
All trees:
Char. 13: 1 --> 2
Char. 44: 0 --> 1

Node 164 :
All trees:
Char. 16: 0 --> 1
Char. 18: 2 --> 1
Char. 105: 0 --> 1
Char. 115: 0 --> 1
Char. 156: 0 --> 1
Char. 165: 2 --> 1

Node 165 :
All trees:
Char. 80: 2 --> 1

Node 166 :
All trees:
Char. 22: 0 --> 1
Char. 39: 0 --> 1

Node 167 :
All trees:
Char. 88: 0 --> 1
Some trees:
Char. 59: 0 --> 1

Node 168 :
All trees:
Char. 55: 0 --> 1
Char. 56: 1 --> 0
Char. 57: 0 --> 1
Char. 113: 1 --> 0
Char. 135: 0 --> 1
Char. 141: 0 --> 1
Char. 144: 0 --> 1
Char. 191: 0 --> 1
Char. 206: 0 --> 1
Some trees:
Char. 95: 0 --> 1

Node 169 :
All trees:
Char. 8: 0 --> 1
Char. 137: 0 --> 1

Node 170 :
All trees:
Char. 54: 1 --> 0
Char. 64: 0 --> 1
Some trees:
Char. 25: 0 --> 2
Char. 26: 0 --> 1
Char. 157: 0 --> 1

Node 171 :
All trees:
Char. 46: 0 --> 1

Node 172 :
All trees:
Char. 43: 1 --> 0
Char. 48: 2 --> 0
Char. 70: 1 --> 2
Some trees:
Char. 27: 0 --> 1
Char. 140: 1 --> 0
Char. 169: 0 --> 1

Node 173 :
All trees:
Char. 9: 1 --> 0
Char. 18: 1 --> 0
Char. 64: 0 --> 1
Some trees:
Char. 102: 1 --> 0

Node 174 :

All trees:
Char. 26: 0 --> 2
Char. 28: 0 --> 1
Char. 45: 0 --> 1
Char. 200: 1 --> 0
Char. 216: 0 --> 1

Node 175 :
All trees:
Char. 116: 1 --> 0

Node 176 :
All trees:
Char. 184: 0 --> 1

Node 177 :
All trees:
Char. 241: 1 --> 2

Node 178 :
All trees:
Char. 207: 0 --> 1
Char. 230: 1 --> 0
Char. 233: 1 --> 0
Some trees:
Char. 219: 0 --> 2

Node 179 :
All trees:
Char. 192: 1 --> 0
Some trees:
Char. 59: 0 --> 1

Node 180 :
All trees:
Char. 70: 1 --> 0
Char. 174: 2 --> 3

Node 181 :
All trees:
Char. 26: 0 --> 3

Node 182 :
All trees:
Char. 25: 0 --> 1
Char. 73: 0 --> 1
Char. 78: 0 --> 1
Char. 110: 0 --> 1
Char. 113: 0 --> 1

Node 183 :
All trees:
Char. 4: 0 --> 1
Char. 53: 0 --> 1

Node 184 :
All trees:
Char. 140: 1 --> 0

Node 185 :
All trees:
Char. 88: 0 --> 1

Node 186 :
All trees:
Char. 90: 0 --> 1

Node 187 :
All trees:
Char. 54: 1 --> 0
Char. 55: 1 --> 2
Char. 69: 0 --> 1
Char. 74: 1 --> 2
Char. 75: 0 --> 1
Char. 89: 0 --> 1
Char. 127: 0 --> 1

Node 188 :
All trees:
Char. 76: 0 --> 1

Node 189 :
All trees:
Char. 32: 1 --> 0
Char. 77: 0 --> 2
Char. 79: 0 --> 1
Char. 114: 1 --> 2

Node 190 :
All trees:
Char. 77: 0 --> 1
Char. 87: 1 --> 0

Node 191 :
All trees:
Char. 124: 0 --> 1
Char. 129: 1 --> 2

Node 192 :
All trees:
Char. 45: 1 --> 0

Node 193 :
All trees:
Char. 34: 0 --> 1
Char. 35: 0 --> 1
Char. 58: 0 --> 1

Section 3: Constrained phylogenetic analysis results

3.1. Detailed description of the results

Although South American and Australasian chelids were forced to form clades (Fig. S7), the extinct chelids maintain the same position as in the original tree (Fig. S8), with *Chelus colombianus* as sister taxon to *C. fimbriatus*, and the other fossils grouped in a clade sister to *Hydromedusa* spp. Araripemydidae, Euraxemydidae, Bothremydidae and non-Podocnemididae Podocnemidoidae taxa maintain the same positions, but *Sokatra antitra* is in a politomy including Pan-Pelomedusidae and Pan-Podocnemididae (Fig. S8). *Erymnochelys madagascariensis* is forced closer to *Podocnemis* spp. than to *Peltocephalus dumerilianus* (Fig. S7), but the latter is still recovered as sister to Stereogenyini, and *Caninemys tridentata* and *Cerrejonemys wayuunaki* form successive sister taxa to *Podocnemis* spp as well (Fig. S8). Similarly, the taxa closer to *E. madagascariensis* (e.g. *Turkanemys pattersoni*, *Neochelys franzeni* and *Kenyemys williamsi*) were again recovered in the same clade (Fig. S8). On the other hand, *Carbonemys cofrinii*, *Dacquemys paleomorpha*, *Stupendemys geographicus*, and UCMP 42008, recovered in a clade inside Erymnochelyinae in the unconstrained analysis, were not grouped together and appear in a politomy with the other podocnemidid clades (Fig. S8).

3.2. Constrained clades

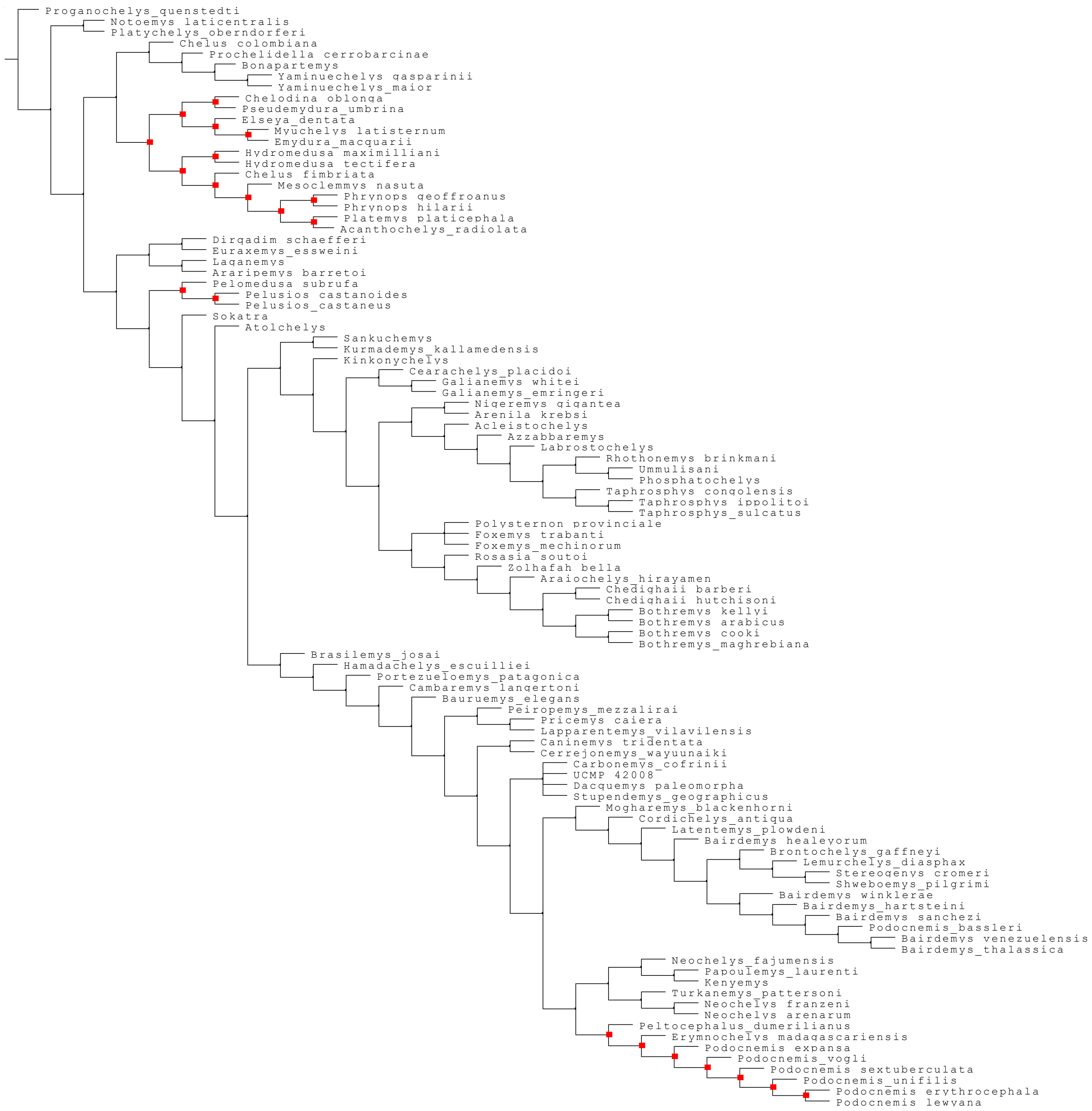

Supplementary Figure 7. Arbitrary tree highlighting constrained clades with red markers enforced in the constrained analysis.

3.3. Strict consensus tree with node numbers

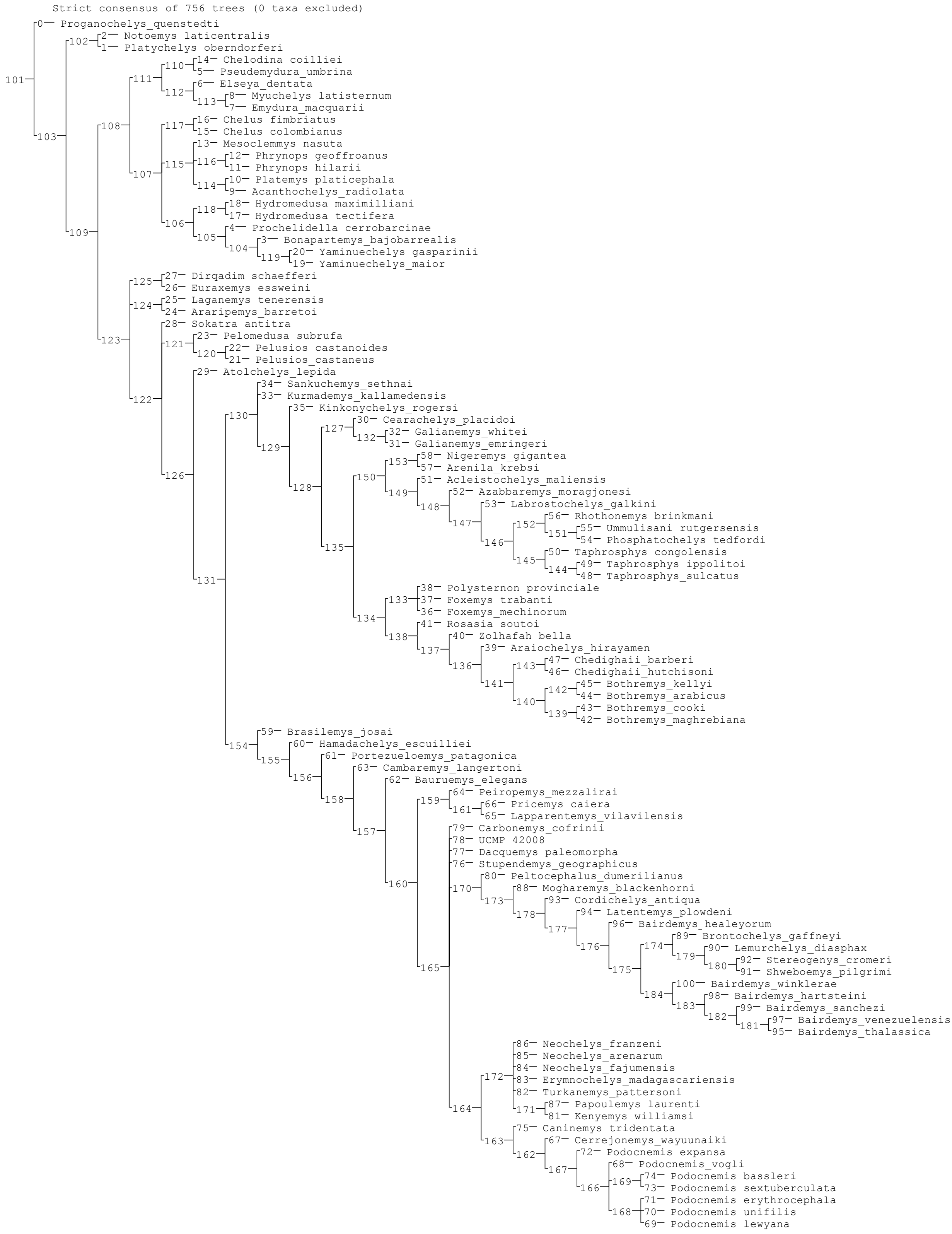

Supplementary Figure 8. Strict consensus tree of 738 MPTs of 1175 steps obtained by the constrained analysis.

Section 4: Additional trees

4.1. Procedure to create fully dichotomic trees

The biogeographic analysis requires fully dichotomic topologies, hence, the polytomies of the original tree had to be manually resolved by deliberately choosing an arrangement. However, some considerations were taken in order to minimize the effect of arbitrariness related to this procedure. As advocated by previous studies (e.g., Upchurch *et al.*, 2015), one alternative is to define clades in a way that the arrangement minimizes biogeographic changes (e.g. in a polytomy including two taxa in one area and a third in another, we define the first two as sister-taxa in exclusion of the latter). This was possible to be done for one case of the original tree ((*Carbonemys cofrinii* + *Stupendemys geographica*) + (*Dacquemys paleomorpha* + UCMP 42008)). In cases when this was not possible, two different procedures were conducted: (1) define the relationship based on previous studies and never contradicted posteriorly (e.g. *Foxemys mechinorum* + *Foxemys trabanti* in exclusion of *Polysternon provinciale*); or (2) using an arrangement found in one of the MPTs (e.g. *Sankuchemys sethnai* + *Kurmademys kallamedensis* in exclusion of *Kinkonychelys rogersi* and Bothremydini).

4.2. Supertree

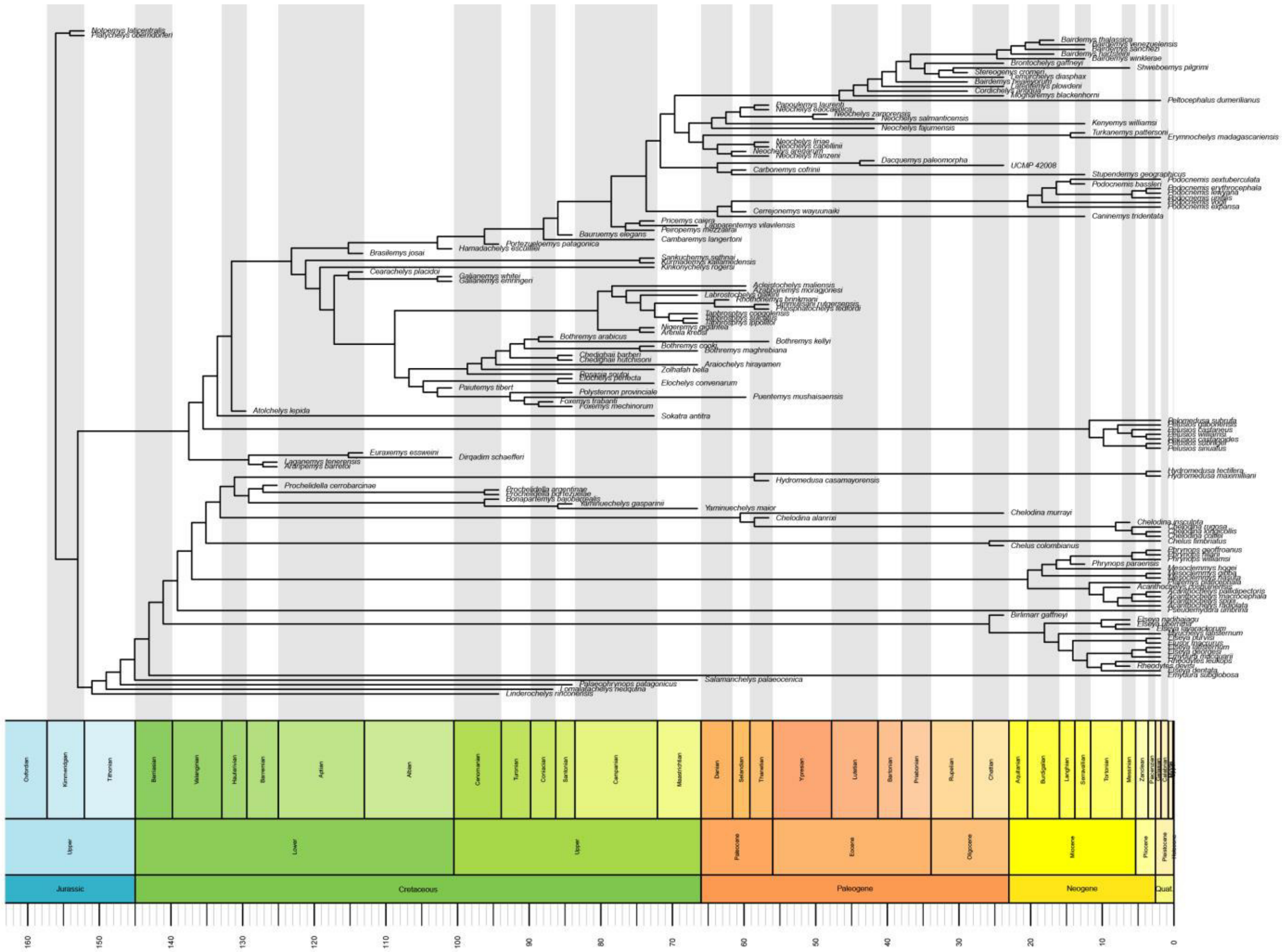

Supplementary Figure 9. Supertree used in the diversification and diversity analyses. Topology constructed on Mesquite v. 3.0 (Maddison & Maddison, 2009) by inserting taxa on Supplementary Table 1 and time-scaled using R package *strap* (Bell & Lloyd 2014) using Brusatte et al. (2008) method.

Supplementary Table 1. Taxa of the original topology and age data used for timetree estimates. For the sake of simplicity during biogeographic analyses terminal taxa were treated as having a temporal spam of 1 Myr. FAD, first appearance datum; LAD, last appearance datum. Age data extracted from the literature and supplemented by Paleobiology Database (https://paleobiodb.org/).

Taxon	FAD [Myr]	LAD [Myr]	Taxon	FAD [Myr]	LAD [Myr]
Platychelys oberndorferi	152.1	145.0	*Acleistochelys maliensis*	59.2	56.0
Notoemys laticentralis	152.1	145.0	*Azabbaremys moragjonesi*	59.2	56.0
Bonapartemys bajobarrealis	93.9	72.1	*Labrostochelys galkini*	66.0	61.6
Prochelidella cerrobarcinae	125.0	100.5	*Phosphatochelys tedfordi*	56.0	47.8
Pseudemydura umbrina	1.0	0.0	*Ummulisani rutgersensis*	56.0	47.8
Elseya dentata	1.0	0.0	*Rhothonemys brinkmani*	61.6	59.2
Emydura macquarii	1.0	0.0	*Arenila krebsi*	72.1	66.0
Myuchelys latisternum	1.0	0.0	*Nigeremys gigantea*	72.1	66.0
Acanthochelys radiolata	1.0	0.0	*Brasilemys josai*	113.0	100.5
Platemys platicephala	1.0	0.0	*Hamadachelys escuilliei*	100.5	93.9
Phrynops hilarii	1.0	0.0	*Portezueloemys patagonica*	93.9	89.8
Phrynops geoffroanus	1.0	0.0	*Bauruemys elegans*	83.6	72.1
Mesoclemmys nasuta	1.0	0.0	*Cambaremys langertoni*	72.1	66.0
Chelodina oblonga	1.0	0.0	*Peiropemys mezzalirai*	72.1	66.0
Chelus colombianus	23.03	5.33	*Lapparentemys vilavilensis*	66.0	61.6
Chelus fimbriatus	1.0	0.0	*Pricemys caiera*	72.1	66.0
Hydromedusa tectifera	1.0	0.0	*Cerrejonemys wayuunaiki*	59.2	56.0
Hydromedusa maximilliani	1.0	0.0	*Podocnemis vogli*	1.0	0.0
Yaminuechelys maior	66.0	61.6	*Podocnemis lewyana*	1.0	0.0
Yaminuechelys gasparinii	83.6	66.0	*Podocnemis unifilis*	1.0	0.0
Pelusios castaneus	1.0	0.0	*Podocnemis erythrocephala*	1.0	0.0
Pelusios castanoides	1.0	0.0	*Podocnemis expansa*	1.0	0.0
Pelomedusa subrufa	1.0	0.0	*Podocnemis sextuberculata*	1.0	0.0
Araripemys barretoi	125.0	100.5	*Podocnemis bassleri*	11.63	5.33
Laganemys tenerensis	125.0	100.5	*Caninemys tridentata*	11.63	5.33
Euraxemys essweini	113.0	100.5	*Stupendemys geographicus*	11.63	5.33
Dirqadim schaefferi	100.5	93.9	*Dacquemys paleomorpha*	41.2	33.9

Sokatra antitra	72.1	66.0	*UCMP 42008*	23.03	15.97
Atolchelys lepida	129.4	125.0	*Carbonemys cofrinii*	59.2	56.0
Cearachelys placidoi	113.0	100.5	*Peltocephalus dumerilianus*	1.0	0.0
Galianemys emringeri	100.5	93.9	*Kenyemys williamsi*	11.63	5.33
Galianemys whitei	100.5	93.9	*Turkanemys pattersoni*	11.63	5.33
Kurmademys kallamedensis	72.1	66.0	*Erymnochelys madagascariensis*	1.0	0.0
Sankuchemys sethnai	72.1	66.0	*Neochelys fajumensis*	41.2	33.9
Kinkonychelys rogersi	72.1	66.0	*Neochelys arenarum*	59.2	56.0
Foxemys mechinorum	83.6	72.1	*Neochelys franzeni*	56.0	47.8
Foxemys trabanti	86.3	72.1	*Papoulemys laurenti*	56.0	47.8
Polysternon provinciale	83.6	72.1	*Mogharemys blackenhorni*	23.03	15.97
Araiochelys hirayamen	66.0	61.6	*Brontochelys gaffneyi*	23.03	15.97
Zolhafah bella	72.1	66.0	*Lemurchelys diasphax*	23.03	15.97
Rosasia soutoi	83.6	72.1	*Shweboemys pilgrimi*	5.33	2.58
Bothremys maghrebiana	66.0	61.6	*Stereogenys cromeri*	28.1	23.03
Bothremys cooki	72.1	66.0	*Cordichelys antiqua*	28.1	23.03
Bothremys arabicus	86.3	83.6	*Latentemys plowdeni*	23.03	15.97
Bothremys kellyi	56.0	47.8	*Bairdemys thalassica*	15.97	11.63
Chedighaii hutchisoni	83.6	72.1	*Bairdemys healeyorum*	28.1	23.03
Chedighaii barberi	83.6	72.1	*Bairdemys venezuelensis*	11.63	5.33
Taphrosphys sulcatus	66.0	61.6	*Bairdemys hartsteini*	15.97	11.63
Taphrosphys ippolitoi	66.0	61.6	*Bairdemys sanchezi*	11.63	5.33
Taphrosphys congolensis	66.0	61.6	*Bairdemys winklerae*	11.63	5.33

Supplementary Table 2. Additional taxa inserted in the original topology to compose the Supertree (Supplementary Figure 9). For the sake of simplicity during biogeographic analyses terminal taxa were treated as having a temporal spam of 1 Myr. FAD, first appearance datum; LAD, last appearance datum. Age data extracted from the literature and supplemented by Paleobiology Database (https://paleobiodb.org/).

**Taxon**	**FAD [Myr]**	**LAD [Myr]**	**Taxon**	**FAD [Myr]**	**LAD [Myr]**
Acanthochelys cosquinensis	5.3	2.58	*Lomalatachelys neuquina*	86.3	83.6
Acanthochelys macrocephala	1.0	0.0	*Mesoclemmys gibba*	1.0	0.0
Acanthochelys pallidipectoris	1.0	0.0	*Mesoclemmys hogei*	1.0	0.0
Acanthochelys spixii	1.0	0.0	*Neochelys capellinii*	56.0	41.2
Birlimarr gaffneyi	23.03	5.33	*Neochelys eaocaenica*	56.0	41.2
Chelodina alanrixi	56.0	33.9	*Neochelys liriae*	56.0	47.8
Chelodina insculpta	5.3	1.0	*Neochelys salmanticensis*	41.2	37.8
Chelodina longicollis	1.0	0.0	*Neochelys zamorensis*	47.8	41.2
Chelodina murrayi	23.03	11.63	*Paiutemys tibert*	100.5	93.9
Chelodina rugosa	1.0	0.0	*Palaeophrynops patagonicus*	83.6	66.0
Elochelys convenarum	72.1	66.0	*Pelusios gabonensis*	1.0	0.0
Elochelys perfecta	83.6	72.1	*Pelusios sinuatus*	1.0	0.0
Elseya georgesi	1.0	0.0	*Pelusios subniger*	1.0	0.0
Elseya latisternum	1.0	0.0	*Pelusios williamsi*	1.0	0.0
Elseya lavarackorum	2.58	1.0	*Phrynops paraensis*	11.63	5.33
Elseya nadibajagu	5.3	2.58	*Phrynops williamsi*	1.0	0.0
Elseya purvisi	1.0	0.0	*Prochelidella argentinae*	93.9	72.1
Elseya uberrima	5.3	2.58	*Prochelidella portezuelae*	93.9	86.3
Elusor macrurus	1.0	0.0	*Puentemys mushaisaensis*	59.2	56.0
Emydura subglobosa	1.0	0.0	*Rheodytes devisi*	5.3	2.58
Hydromedusa casamayorensis	56.0	33.9	*Rheodytes leukops*	1.0	0.0
Linderochelys rinconensis	93.9	86.3	*Salamanchelys palaeocenica*	66.0	61.6

4.3. ‘Non-marine taxa’ tree

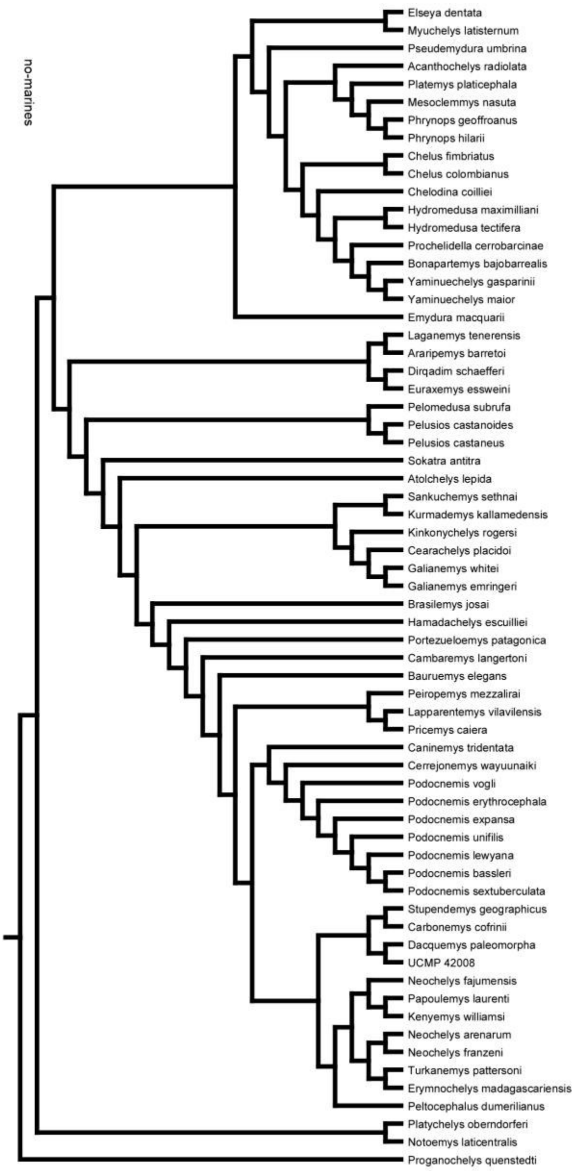

Supplementary Figure 10. Topology referred to as ‘non-marine taxa tree’ used in the biogeographical ancestral area reconstructions. Topology constructed on Mesquite v. 3.0 (Maddison & Maddison, 2009) by removing taxa on Supplementary Table 2.

Supplementary Table 3. Taxa pruned from the original taxon list for construction of the 'non-marine taxa tree'.

Taxon	Clade	Taxon	Clade
Acleistochelys maliensis	Bothremydidae	*Foxemys mechinorum*	Bothremydidae
Araiochelys hirayamen	Bothremydidae	*Foxemys trabanti*	Bothremydidae
Arenila krebsi	Bothremydidae	*Labrostochelys galkini*	Bothremydidae
Azabbaremys moragjonesi	Bothremydidae	*Latentemys plowdeni*	Stereogenyina
Bairdemys hartsteini	Stereogenyina	*Lemurchelys diasphax*	Stereogenyina
Bairdemys healeyorum	Stereogenyina	*Mogharemys blackenhorni*	Stereogenyina
Bairdemys sanchezi	Stereogenyina	*Nigeremys gigantea*	Bothremydidae
Bairdemys thalassica	Stereogenyina	*Phosphatochelys tedfordi*	Bothremydidae
Bairdemys venezuelensis	Stereogenyina	*Polysternon provinciale*	Bothremydidae
Bairdemys winklerae	Stereogenyina	*Rhothonemys brinkmani*	Bothremydidae
Bothremys arabicus	Bothremydidae	*Rosasia soutoi*	Bothremydidae
Bothremys cooki	Bothremydidae	*Shweboemys pilgrimi*	Stereogenyina
Bothremys kellyi	Bothremydidae	*Stereogenys cromeri*	Stereogenyina
Bothremys maghrebiana	Bothremydidae	*Taphrosphys congolensis*	Bothremydidae
Brontochelys gaffneyi	Stereogenyina	*Taphrosphys ippolitoi*	Bothremydidae
Chedighaii barberi	Bothremydidae	*Taphrosphys sulcatus*	Bothremydidae
Chedighaii hutchisoni	Bothremydidae	*Ummulisani rutgersensis*	Bothremydidae
Cordichelys antiqua	Stereogenyina	*Zolhafah bella*	Bothremydidae

4.4. Time bin subtrees for diversification shifts analyses

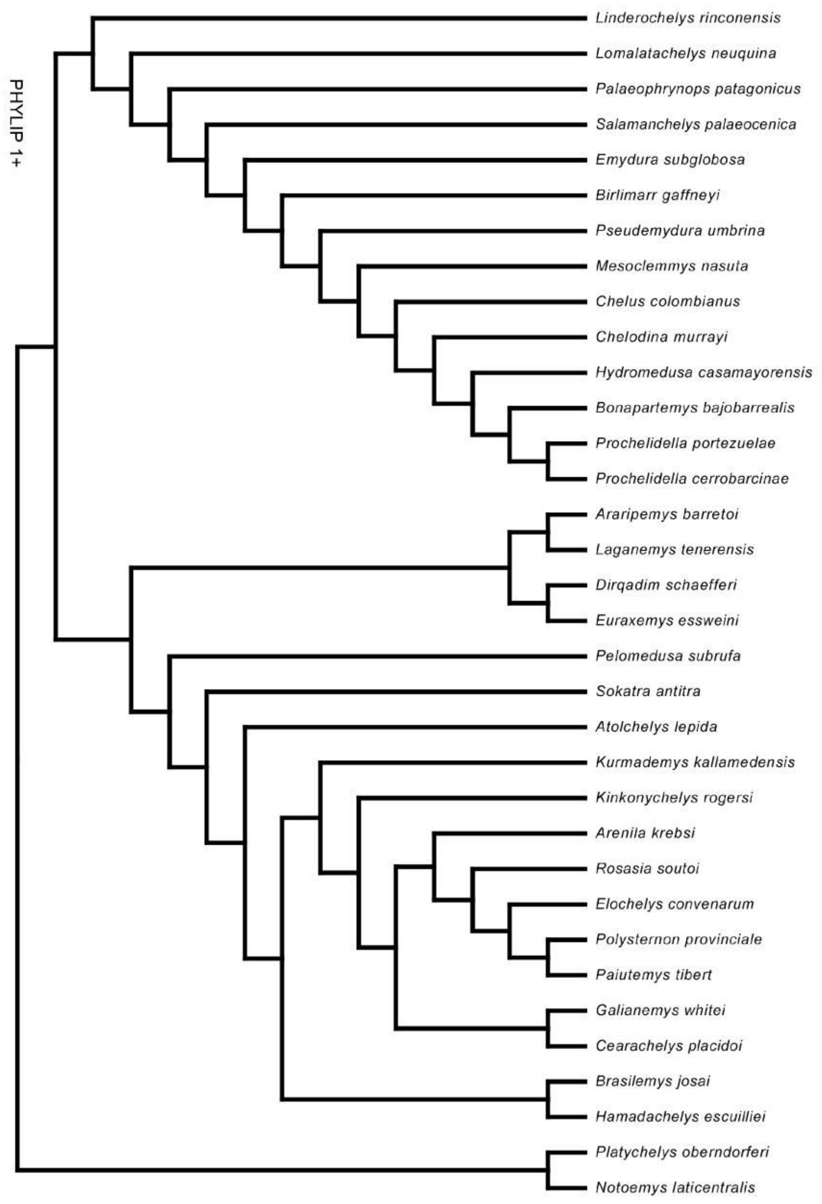

Supplementary Figure 11. Subtree of the 'supertree' used as the Time Bin 1 (Early Cretaceous) of the diversification analysis. Topology constructed on Mesquite v. 3.0 (Maddison & Maddison, 2009) by pruning taxa on Supplementary Figure 9.

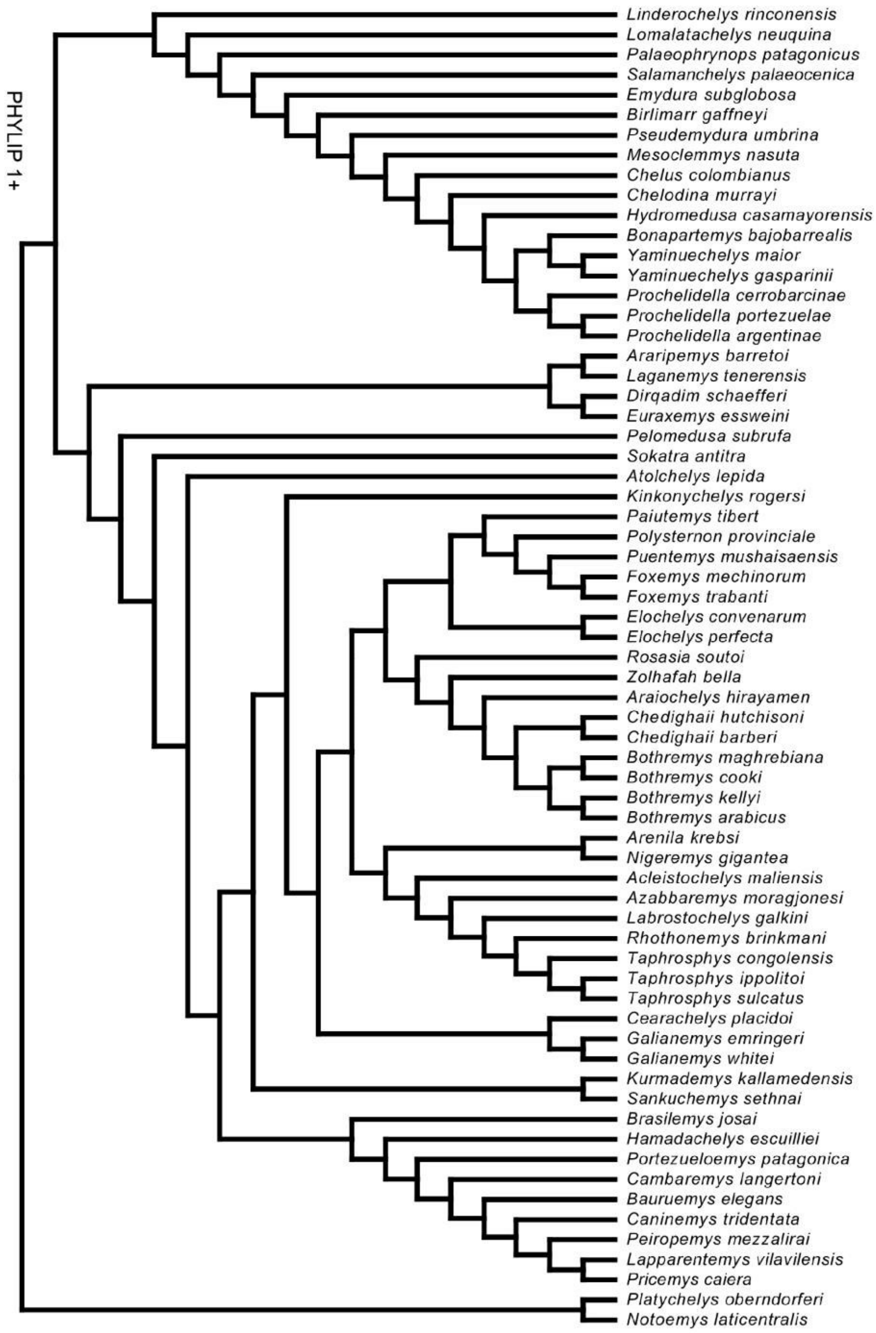

Supplementary Figure 12. Subtree of the 'supertree' used as the Time Bin 2 (Late Cretaceous) of the diversification analysis. Topology constructed on Mesquite v. 3.0 (Maddison & Maddison, 2009) by pruning taxa on Supplementary Figure 9.

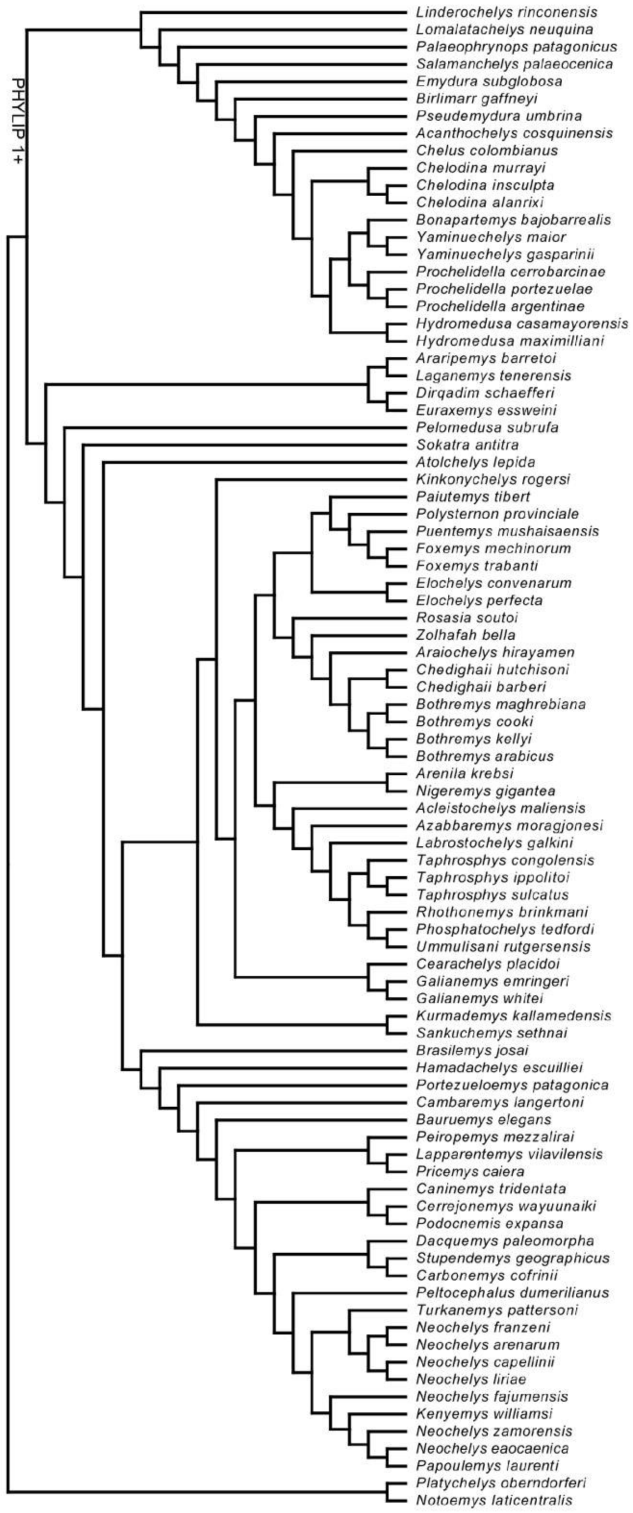

Supplementary Figure 13. Subtree of the 'supertree' used as the Time Bin 3 (Paleocene) of the diversification analysis. Topology constructed on Mesquite v. 3.0 (Maddison & Maddison, 2009) by pruning taxa on Supplementary Figure 9.

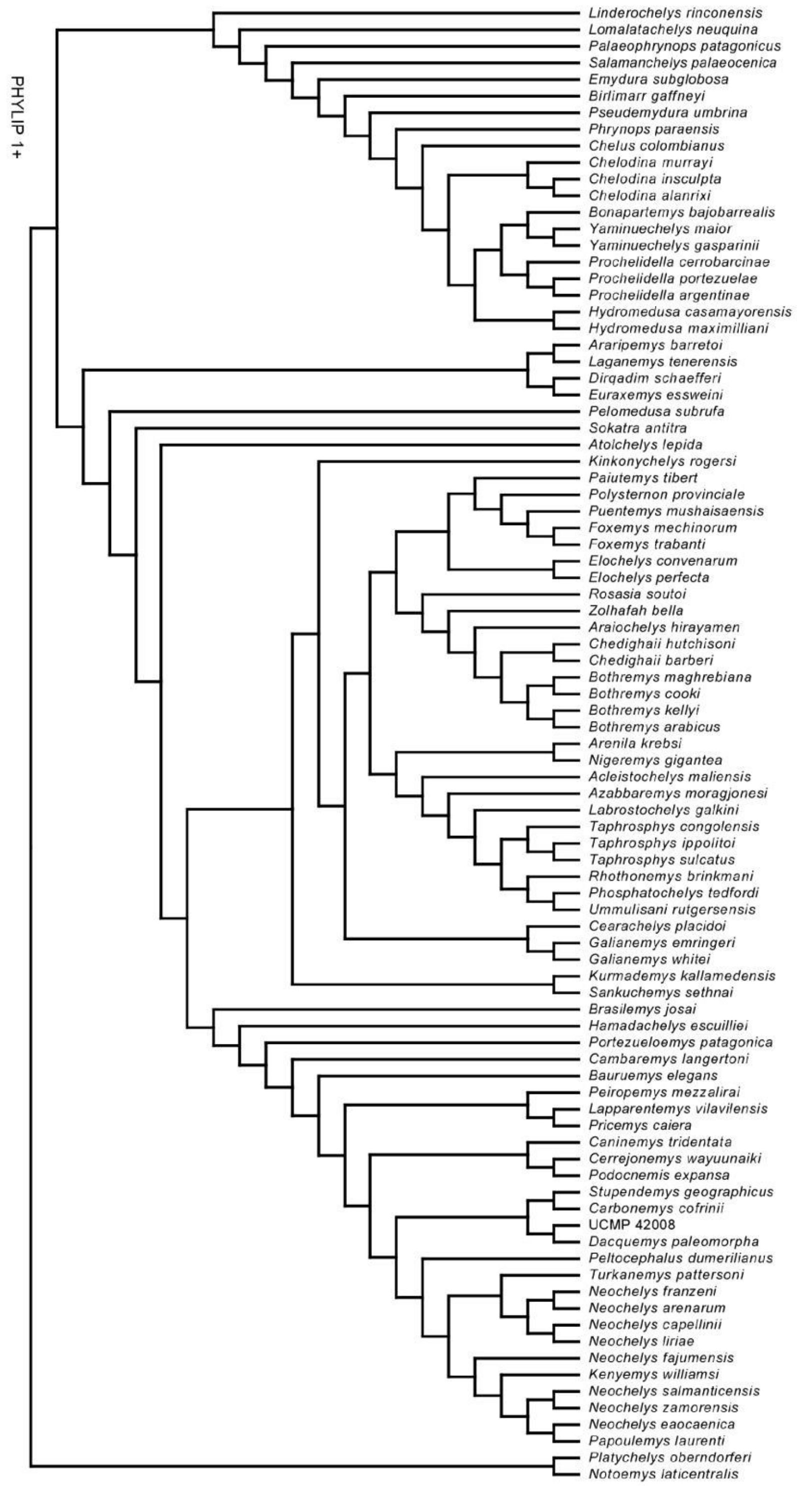

Supplementary Figure 14. Subtree of the 'supertree' used as the Time Bin 4 (Eocene) of the diversification analysis. Topology constructed on Mesquite v. 3.0 (Maddison & Maddison, 2009) by pruning taxa on Supplementary Figure 9.

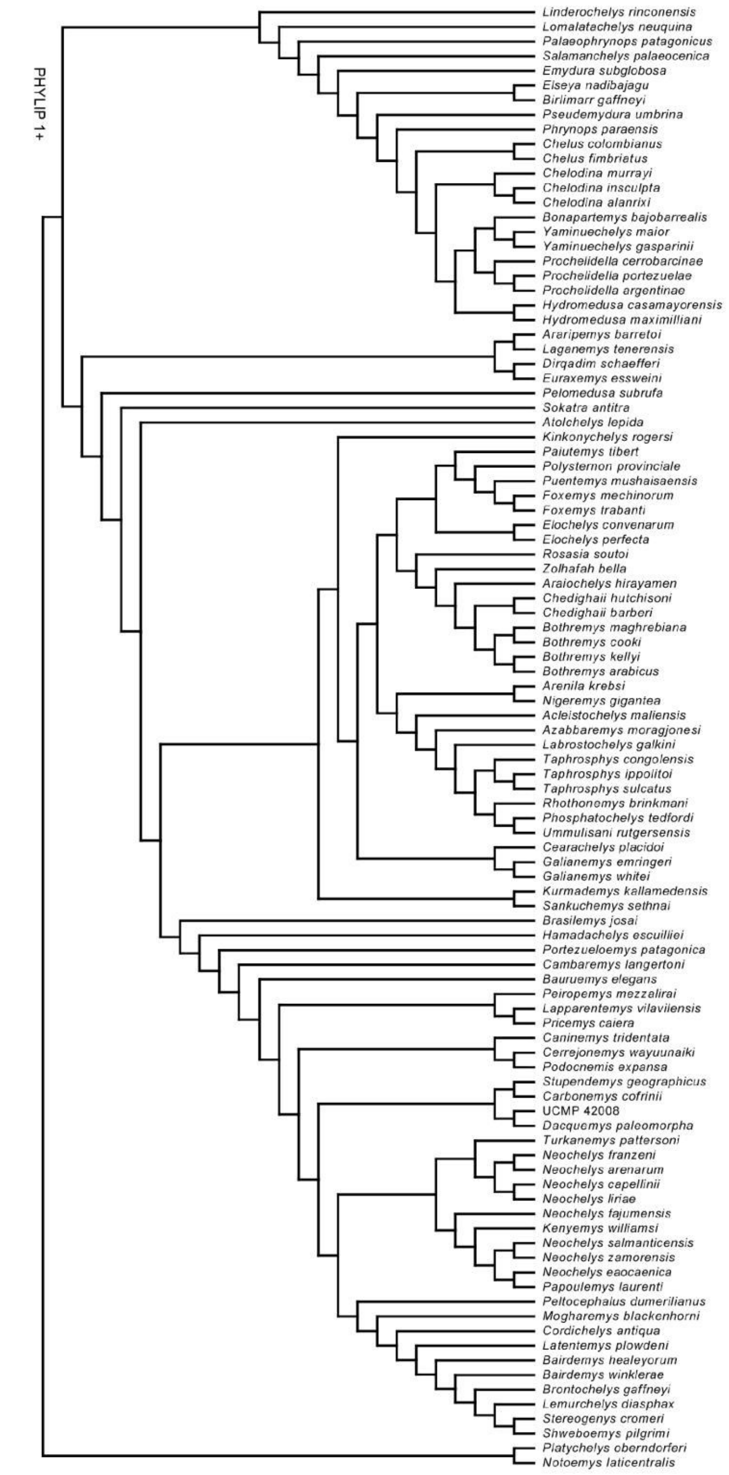

Supplementary Figure 15. Subtree of the 'supertree' used as the Time Bin 5 (Oligocene) of the diversification analysis. Topology constructed on Mesquite v. 3.0 (Maddison & Maddison, 2009) by pruning taxa on Supplementary Figure 9.

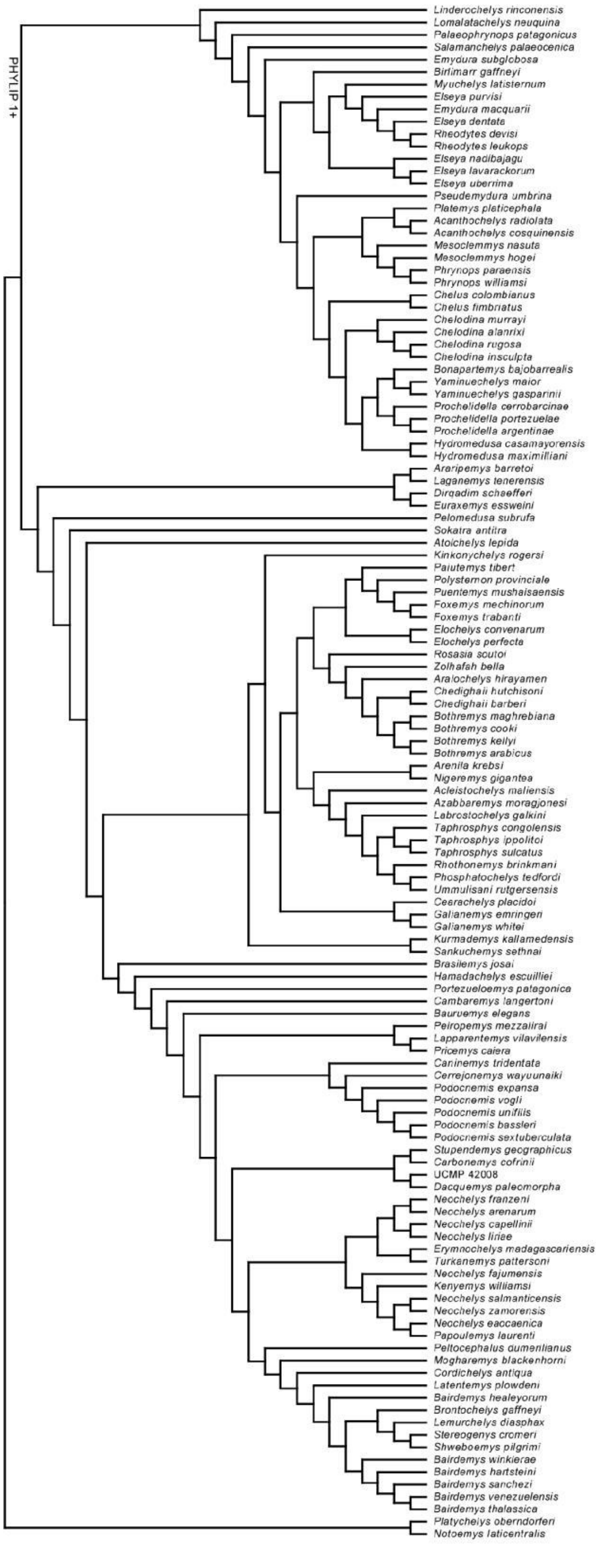

Supplementary Figure 16. Subtree of the ‘supertree’ used as the Time Bin 6 (Miocene) of the diversification analysis. Topology constructed on Mesquite v. 3.0 (Maddison & Maddison, 2009) by pruning taxa on Supplementary Figure 9.

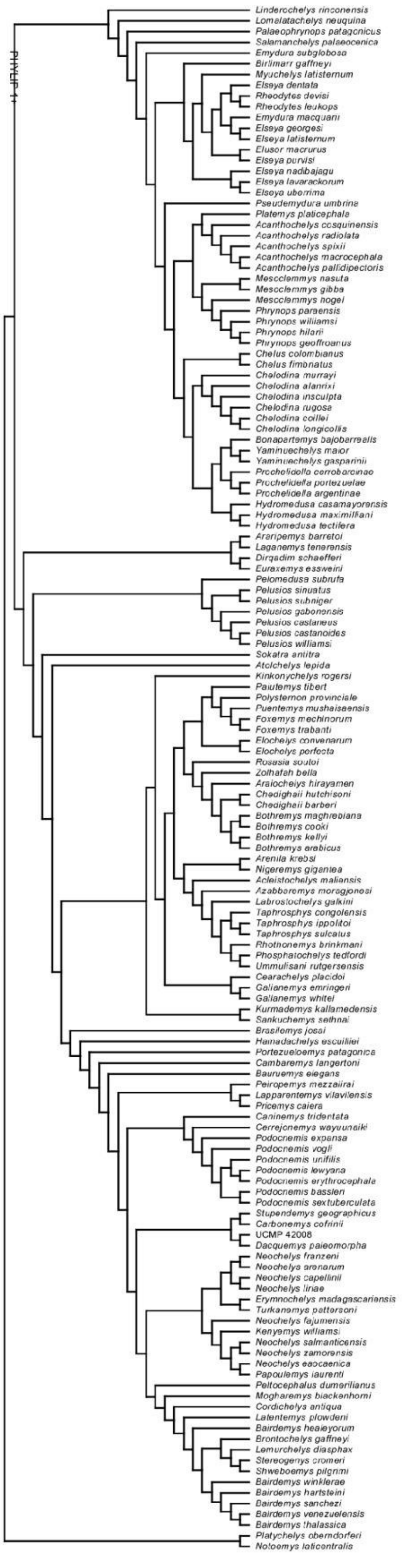

Supplementary Figure 17. Subtree of the 'supertree' used as the Time Bin 7 (Pliocene-Recent) of the diversification analysis. Topology constructed on Mesquite v. 3.0 (Maddison & Maddison, 2009) by pruning taxa on Supplementary Figure 9.

Section 5: Biogeographic analyses

5.1. Ancestral area reconstruction results and original outputs

Ancestral area reconstruction generally agrees between the best-fitted models (DIVALIKE-M_2 and DEC- M_2), with important exceptions being the areas of four nodes, namely Pelomedusoides + Araripemydidae, Araripemydidae + Euraxemydidae, Araripemydidae, and Euraxemydidae. DIVALIKE- M_2 favors an African distribution in all three topologies (Fig. 2), whereas DEC- M_2 favors South American ancestors (Appendix S2).

As expected, the probabilities for the alternative areas in the Pleurodira node are relatively low, but an African/Australian distribution is best supported by DIVALIKE-M_2, and African/Australian/South American distribution by DEC-M_2. For the Pan-Chelidae node an Australian distribution is favored and one dispersal event to South America occurred during the Early Cretaceous. From this area the ancestors of *Chelodina colliei* dispersed back to Australia before the Aptian (Fig. 2).

Pan-Pelomedusoides were distributed in Africa (Fig. 2, 3), with Pan-Pelomedusidae remaining in this continent, while its sister-clade, Pan-Podocnemididae, dispersed to South America during the Early Cretaceous. Other South America/Africa duplets are found during this period, for example in Araripemydidae, Euraxemydidae, Podocnemidoidea, Cearachelyini, and *Hamadachelys* + other Podocnemidoidae (Fig. 2, 3). However, in none of these cases a joint distribution is favored in the M_2 models, making it unlike that the descendant ranges are a result of vicariance in the best-fitted model (Ronquist & Sanmartín, 2011; Matzke, 2013). Bothremydids most likely originated in Africa and experienced several dispersal events from this area. Still during the Early Cretaceous the ancestor of the clade *Sankuchemys* + *Kurmademys* jumped to India and *Kinkonychelys rogersi* to Madagascar, while the Cearachelyini and Bothremydini ancestors dispersed, respectively, to South America and Europe. From the latter area, bothremydinis dispersed back to Africa and from there to North America twice during the Late Cretaceous, in the *Chedighaii* + *Bothremys* and in *B. cooki* + *B. maghrebiana* nodes (Fig. 2). A third dispersal event to North America occurred later by the end of the Cretaceous, when *Taphrosphys sulcatus* reached this area from the African Taphrosphyini ancestors (Fig. 2).

Two additional South America/Africa duplets occurred in the Podocnemididae clade. The Podocnemidinae node maintained the South American distribution while the ancestor of Erymnochelyinae dispersed to Africa, as did that of *Dacquemys paleomorpha* + UCMP 42008, both during the Late Cretaceous (Fig. 3). During the Paleogene podocnemidids experienced a series of dispersal events from African ancestors, with *Neochelys franzeni* + *Neo. arenarum* and *Papoulemys*

laurenti jumping to Europe, *Erymnochelys madagascariensis* to Madagascar, and *Peltocephalus dumerilianus* to South America. By the end of the Eocene and during the Oligocene, the Stereogenyini (similarly to the Bothremydini) experienced at least four dispersal events from African ancestors to North America, Asia (twice) and South America (Fig. 3).

Removing the marine taxa (Appendices S5, S6) from the analyses did not alter the results described above, except for the Bothremydidae node, which is estimated as distributed in Madagascar during the Early Cretaceous. On the other hand, the analysis of the 'molecular constrained tree' showed noteworthy differences (Appendices S7, S8). The Pan-Chelidae ancestor occupied the South American continent and from there, the ancestor of all Australian chelids dispersed to this area still during the Early Cretaceous. The different arrangements of *Erymnochelys*, *Peltocephalus*, and *Podocnemis* also affected the ancestral area reconstructions. The clade including *Erymnochelys* but not *Podocnemis* nor *Peltocephalus* dispersed from South America to Europe during the Late Cretaceous and from there to Africa by the end of this period. Also at this time, *Erymnochelys* ancestor jumped to Madagascar and it is restricted to this subcontinent since then. The clade including *Peltocephalus* and Stereogenyini, but not *Erymnochelys* nor *Podocnemis* were distributed in South America, instead of Africa as for the original tree analysis (Fig. 3). From these areas, Stereogenyini dispersed to Africa during the beginning of the Paleocene and then to the other areas during the Eocene and Oligocene.

Supplementary Table 4. Summary of results and statistical comparisons between the various models (i.e., DEC, DIVALIKE and BAYAREA, M_0, M_1 and M_2 models) used for the three sets of analyses (i.e., using the 'original', 'non-marine taxa' and 'supertree' topologies). Abbreviations: AIC, Akaike information criterion; AICwt, weight Akaike information criterion; LnL, likelihood; P, number of free parameters in the model; d, e, j, and x are the estimated parameter values for each model.

Tree	Model	LnL	P	d	e	j	x	AIC	AICwt
original	DEC-M_0	-209.30	2	0.0003	0.0028	0	1	422.59	$4.0e^{-18}$
	DEC-M_1	-191.71	3	0.0006	0.0008	0	-0.0003	389.42	$6.5e^{-11}$
	DEC-M_2	-135.82	4	$1.0e^{-12}$	$1.0e^{-12}$	0.0232	-0.0013	279.64	0.4459
	DIVALIKE-M_0	-231.30	2	0.0004	0.0075	0	1	466.60	$1.1e^{-27}$
	DIVALIKE-M_1	-203.47	3	0.0009	0.0011	0	0.0045	412.93	$5.1e^{-16}$
	DIVALIKE-M_2	-135.64	4	$1.0e^{-12}$	$1.0e^{-12}$	0.0209	0.0487	279.28	0.5327
	BAYAREA-M_0	-253.50	2	0.0002	0.0165	0	1	51.100	$2.6e^{-37}$
	BAYAREA-M_1	-245.92	3	0.0007	0.0159	0	-0.0072	497.84	$1.8e^{-35}$
	BAYAREA-M_2	-138.86	4	$1.0e^{-07}$	$1.0e^{-07}$	0.0238	-0.0102	285.71	0.0214
non-marine taxa	DEC-M_0	-134.58	2	0.0002	0.0053	0	1	273.17	$2.7e^{-09}$
	DEC-M_1	-122.36	3	0.0006	0.0015	0	-0.0039	250.72	$2.0e^{-04}$
	DEC-M_2	-81.83	4	$1.0e^{-12}$	$1.0e^{-12}$	0.0205	-0.0166	171.67	0.2957
	DIVALIKE-M_0	-143.28	2	0.0003	0.0084	0	1	290.56	$4.5e^{-13}$
	DIVALIKE-M_1	-121.82	3	0.0006	0.0007	0	0.0140	249.65	$3.4e^{-04}$
	DIVALIKE-M_2	-80.97	4	$1.0e^{-12}$	$1.0e^{-12}$	0.0203	0.0092	169.93	0.7043
constrained	DEC-M_0	-195.96	2	0.0003	0.0014	0	1	395.93	$8.1e^{-15}$
	DEC-M_1	-181.54	3	0.0008	0.0013	0	-0.0004	369.09	$5.4e^{-09}$
	DEC-M_2	-129.77	4	$1.0e^{-12}$	$1.0e^{-12}$	0.0212	-0.0182	267.53	0.6150
	DIVALIKE-M_0	-212.15	2	0.0005	0.0034	0	1	428.29	$7.6e^{-22}$
	DIVALIKE-M_1	-190.39	3	0.0011	0.0009	0	0.0037	386.78	$7.9e^{-13}$
	DIVALIKE-M_2	-130.23	4	$1.0e^{-12}$	$1.0e^{-12}$	0.0217	-0.0152	268.47	0.3850

5.2. Biogeographic stochastic mapping of DIVALIKE M_1 and M_2 models outputs

Supplementary Table 5. Number of dispersal events in the history of Pleurodira with biogeographical stochastic mapping. Counts of dispersal events were averaged across the 50 BSMs and are presented here with standard deviations in parentheses. Top table summarizes all dispersal event counts for the model DIVALIKE M_1 and bottom table for DIVALIKE M_2. Ancestral and descendant states are given on the rows and columns, respectively

	**S**	**N**	**E**	**A**	**M**	**I**	**K**	**L**	**O**	**T**
**S**	0.00 (0.00)	0.04 (0.20)	0.12 (0.33)	3.96 (2.01)	0.78 (0.82)	0.48 (0.54)	0.12 (0.33)	0.98 (0.25)	0.00 (0.00)	0.04 (0.20)
**N**	0.00 (0.00)	0.00 (0.00)	0.00 (0.00)	0.00 (0.00)	0.00 (0.00)	0.00 (0.00)	0.00 (0.00)	0.02 (0.14)	0.00 (0.00)	0.00 (0.00)
**E**	0.06 (0.24)	0.00 (0.00)	0.00 (0.00)	0.54 (0.71)	0.00 (0.00)	0.00 (0.00)	0.00 (0.00)	0.00 (0.00)	0.00 (0.00)	0.00 (0.00)
**A**	5.26 (2.15)	4.00 (0.00)	3.40 (0.73)	0.00 (0.00)	1.96 (0.78)	0.32 (0.47)	1.92 (0.40)	0.50 (0.58)	1.00 (0.00)	0.08 (0.27)
**M**	0.18 (0.44)	0.00 (0.00)	0.06 (0.31)	0.14 (0.35)	0.00 (0.00)	0.26 (0.44)	0.00 (0.00)	0.04 (0.20)	0.00 (0.00)	0.00 (0.00)
**I**	0.04 (0.20)	0.00 (0.00)	0.00 (0.00)	0.16 (0.37)	0.10 (0.30)	0.00 (0.00)	0.00 (0.00)	0.00 (0.00)	0.00 (0.00)	0.02 (0.14)
**K**	0.08 (0.27)	0.00 (0.00)	0.00 (0.00)	0.00 (0.00)	0.00 (0.00)	0.00 (0.00)	0.00 (0.00)	0.00 (0.00)	0.00 (0.00)	0.00 (0.00)
**L**	1.26 (0.49)	0.00 (0.00)	0.00 (0.00)	0.20 (0.40)	0.00 (0.00)	0.02 (0.14)	0.00 (0.00)	0.00 (0.00)	0.00 (0.00)	0.00 (0.00)
**O**	0.00 (0.00)	0.00 (0.00)	0.00 (0.00)	0.00 (0.00)	0.00 (0.00)	0.00 (0.00)	0.00 (0.00)	0.00 (0.00)	0.00 (0.00)	0.00 (0.00)
**T**	0.00 (0.00)	0.00 (0.00)	0.00 (0.00)	0.00 (0.00)	0.00 (0.00)	0.00 (0.00)	0.00 (0.00)	0.00 (0.00)	0.00 (0.00)	0.00 (0.00)

	**S**	**N**	**E**	**A**	**M**	**I**	**K**	**L**	**O**	**T**
**S**	0.00 (0.00)	0.00 (0.00)	0.38 (0.57)	4.44 (1.86)	0.96 (0.81)	0.58 (0.50)	0.00 (0.00)	1.04 (0.28)	0.00 (0.00)	0.00 (0.00)
**N**	0.00 (0.00)	0.00 (0.00)	0.00 (0.00)	0.14 (0.50)	0.00 (0.00)	0.00 (0.00)	0.00 (0.00)	0.00 (0.00)	0.00 (0.00)	0.00 (0.00)
**E**	0.44 (0.50)	0.00 (0.00)	0.00 (0.00)	0.76 (0.72)	0.06 (0.24)	0.00 (0.00)	0.00 (0.00)	0.00 (0.00)	0.00 (0.00)	0.00 (0.00)
**A**	4.84 (1.81)	3.98 (0.14)	3.32 (0.71)	0.00 (0.00)	1.80 (0.88)	0.30 (0.46)	1.92 (0.27)	0.24 (0.43)	1.00 (0.00)	0.00 (0.00)
**M**	0.32 (0.51)	0.00 (0.00)	0.02 (0.14)	0.32 (0.55)	0.00 (0.00)	0.12 (0.33)	0.00 (0.00)	0.00 (0.00)	0.00 (0.00)	0.00 (0.00)
**I**	0.04 (0.28)	0.00 (0.00)	0.00 (0.00)	0.14 (0.35)	0.16 (0.37)	0.00 (0.00)	0.00 (0.00)	0.00 (0.00)	0.00 (0.00)	0.00 (0.00)
**K**	0.12 (0.33)	0.00 (0.00)	0.00 (0.00)	0.22 (0.62)	0.00 (0.00)	0.00 (0.00)	0.00 (0.00)	0.00 (0.00)	0.00 (0.00)	0.00 (0.00)
**L**	1.10 (0.36)	0.00 (0.00)	0.02 (0.14)	0.12 (0.33)	0.00 (0.00)	0.00 (0.00)	0.00 (0.00)	0.00 (0.00)	0.00 (0.00)	0.00 (0.00)
**O**	0.00 (0.00)	0.00 (0.00)	0.00 (0.00)	0.00 (0.00)	0.00 (0.00)	0.00 (0.00)	0.00 (0.00)	0.00 (0.00)	0.00 (0.00)	0.00 (0.00)
**T**	0.00 (0.00)	0.00 (0.00)	0.00 (0.00)	0.00 (0.00)	0.00 (0.00)	0.00 (0.00)	0.00 (0.00)	0.00 (0.00)	0.00 (0.00)	0.00 (0.00)

Supplementary Table 6. Number of dispersal events in the history of Pleurodira with biogeographical stochastic mapping. Counts of dispersal events were averaged across the 50 BSMs and are presented here with standard deviations in parentheses. Summary of founder event only counts for DIVALIKE M_2. Ancestral and descendant states are given on the rows and columns, respectively.

	**S**	**N**	**E**	**A**	**M**	**I**	**K**	**L**	**O**	**T**
**S**	0.00 (0.00)	0.00 (0.00)	0.38 (0.57)	4.44 (1.86)	0.96 (0.81)	0.58 (0.50)	0.00 (0.00)	1.04 (0.28)	0.00 (0.00)	0.00 (0.00)
**N**	0.00 (0.00)	0.00 (0.00)	0.00 (0.00)	0.14 (0.50)	0.00 (0.00)	0.00 (0.00)	0.00 (0.00)	0.00 (0.00)	0.00 (0.00)	0.00 (0.00)
**E**	0.44 (0.50)	0.00 (0.00)	0.00 (0.00)	0.76 (0.72)	0.06 (0.24)	0.00 (0.00)	0.00 (0.00)	0.00 (0.00)	0.00 (0.00)	0.00 (0.00)
**A**	4.84 (1.81)	3.98 (0.14	3.32 (0.71)	0.00 (0.00)	1.80 (0.88)	0.30 (0.46)	1.92 (0.27)	0.24 (0.43)	1.00 (0.00)	0.00 (0.00)
**M**	0.32 (0.51)	0.00 (0.00)	0.02 (0.14)	0.32 (0.55)	0.00 (0.00)	0.12 (0.33)	0.00 (0.00)	0.00 (0.00)	0.00 (0.00)	0.00 (0.00)
**I**	0.04 (0.28)	0.00 (0.00)	0.00 (0.00)	0.14 (0.35)	0.16 (0.37)	0.00 (0.00)	0.00 (0.00)	0.00 (0.00)	0.00 (0.00)	0.00 (0.00)
**K**	0.12 (0.33)	0.00 (0.00)	0.00 (0.00)	0.22 (0.62)	0.00 (0.00)	0.00 (0.00)	0.00 (0.00)	0.00 (0.00)	0.00 (0.00)	0.00 (0.00)
**L**	1.10 (0.36)	0.00 (0.00)	0.02 (0.14)	0.12 (0.33)	0.00 (0.00)	0.00 (0.00)	0.00 (0.00)	0.00 (0.00)	0.00 (0.00)	0.00 (0.00)
**O**	0.00 (0.00)	0.00 (0.00)	0.00 (0.00)	0.00 (0.00)	0.00 (0.00)	0.00 (0.00)	0.00 (0.00)	0.00 (0.00)	0.00 (0.00)	0.00 (0.00)
**T**	0.00 (0.00)	0.00 (0.00)	0.00 (0.00)	0.00 (0.00)	0.00 (0.00)	0.00 (0.00)	0.00 (0.00)	0.00 (0.00)	0.00 (0.00)	0.00 (0.00)

Supplementary Table 7. Summary of BSMs event counts for DIVALIKE M1 and M2 models for the 'original tree' dataset. Abbreviations: d, range expansions; e, range contractions; ALL_disp, all dispersal events; ana_disp; anagenetic dispersal events; all_ana, all anagenetic events; all_clado, all cladogenetic events; stdevs; standard deviations.

		founder	d	e	subset	vicariance	sympatry	ALL_disp	ana_disp	all_ana	all_clado	total_events
DIVALIKE M_1	**means**	0	28,1	0	0	27,14	71,86	28,14	28,14	28,14	99	127,1
	**stdevs**	0	1,01	0	0	1,21	1,21	1,01	1,01	1,01	0	1,01
	**sums**	0	1407	0	0	1357	3593	1407	1407	1407	4950	6357
	%	0	22,1	0	0	21,34655	56,52037	22,13308	22,1331	22,133	77,8669	100

		founder	d	e	subset	vicariance	sympatry	ALL_disp	ana_disp	all_ana	all_clado	total_events
DIVALIKE M_2	**means**	28,9	0	0	0	1,76	68,34	28,9	0	0	99	99
	**stdevs**	0.86	0	0	0	0,48	0,92	0,86	0	0	0	0
	**sums**	1445	0	0	0	88	3417	1445	0	0	4950	4950
	%	0,291919	0	0	0	0,017778	0,690303	0,291919	0	0	1	1

Supplementary Table 8. Summary of BSMs event counts for DEC M1 and M2 models for the 'original tree' dataset. Abbreviations same as Table S7.

		founder	d	e	subset	vicariance	sympatry	ALL_disp	ana_disp	all_ana	all_clado	total_events
DEC M_1	**means**	0	19.74	0	23.52	18.48	57	19.74	19.74	19.74	99	118.7
	**stdevs**	0	1.23	0	3.04	1.27	2.76	1.23	1.23	1.23	0	1.23
	**sums**	0	987	0	1176	924	2850	987	987	987	4950	5937
	%	0	16,6246	0	19,808	15,56342	48,00404	16,62456	16,6246	16,625	83,3754	100

		founder	d	e	subset	vicariance	sympatry	ALL_disp	ana_disp	all_ana	all_clado	total_events
DEC M_2	**means**	28.74	0	0	0.82	1.54	67.9	28.74	0	0	99	99
	**stdevs**	1.35	0	0	1.37	0.65	1.13	1.35	0	0	0	0
	**sums**	1437	0	0	41	77	3395	1437	0	0	4950	4950
	%	29,0303	0	0	0,82828	1,555556	68,58586	29,0303	0	0	100	100

Section 6: Supplementary references

Cadena, E, Jaramillo, C, Paramo, ME 2008. New material of *Chelus colombiana* (Testudines; Pleurodira) from the lower Miocene of Colombia. *Journal of Vertebrate Paleontology*, 28, 1206–1212, doi:10.1671/0272-4634-28.4.1206.

Cadena, EA, Jaramillo, CA, Bloch JI 2013b. New material of the platychelyid turtle *Notoemys zapatocaensis* from the Early Cretaceous of Colombia; implications for understanding Pleurodira evolution. In *Morphology and evolution of turtles* (eds DB Brinkman, PA Holroyd, JD Gardner). Springer Netherlands, 105–120, doi:10.1007/978-94-007-4309-0_8.

Cadena, EA, Parham, JF. 2015. Oldest known marine turtle? A new protostegid from the Lower Cretaceous of Colombia. *PaleoBios*, 32, 1–42.

Gaffney, ES, Krause, DW, Zalmout, IS. 2009. *Kinkonychelys*, a new side-necked turtle (Pelomedusoides: Bothremydidae) from the Late Cretaceous of Madagascar. *American Museum Novitates*, 3662, 1–25, doi:10.1206/672.1.

Joyce, WG. 2007. Phylogenetic relationships of Mesozoic turtles. *Bulletin of the Peabody Museum of Natural History*, 48, 3–102.

Joyce, WG, Bell, CJ. 2004. A review of the comparative morphology of extant testudinoid turtles (Reptilia: Testudines). *Asiatic Herpetological Research*, 10, 53–109.

Upchurch, P., Andres, B., Butler, R.J., & Barrett, P.M. (2015) An analysis of pterosaurian biogeography: implications for the evolutionary history and fossil record quality of the first flying vertebrates. Historical Biology, 27, 697-717, doi:10.1080/08912963.2014.939077.

PLATE 1 - Characters 38, 46, 55, 66, 125, 161, 173, 177

38. SQ, lateral surface, origin site of the depressor mandibulae muscles

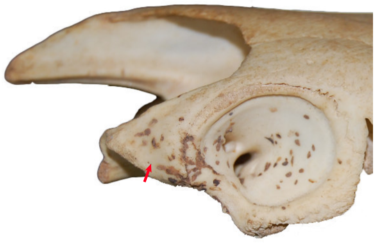

0. convex to slightly flat as in *Podocnemis expansa*

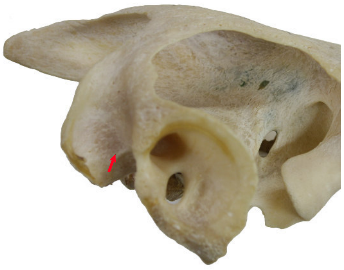

1. strongly concave as in *Elseya dentata*

46. PM, cranial pit on ventral surface

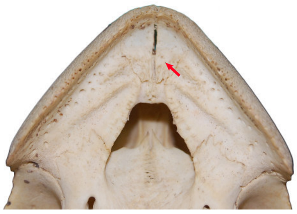

0. no (*Podocnemis expansa*)

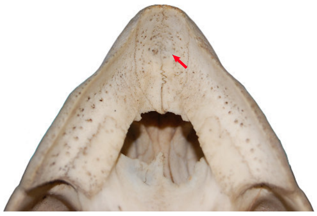

1. yes (*Peltocephalus dumerilianus*)

55. MX, posterior lingual ridge near the maxillar-palatine contact

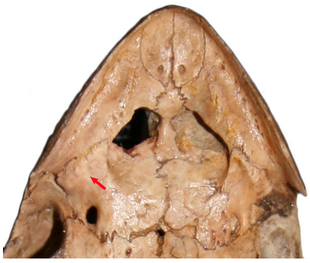

0. undistinguishable or very shallow ridge (*Euraxemys essweini*)

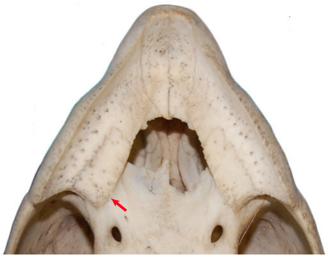

1 well defined ridge (*Peltocephalus dumerilianus*)

66. VO, VO-PM contact

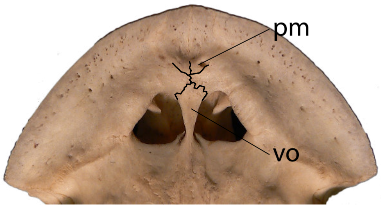

0 absent (*Phrynops geoffroanus*)

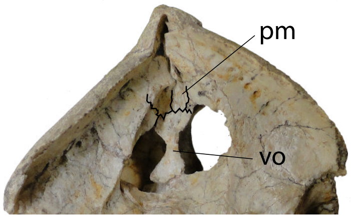

1 present (*Bauruemys elegans*)

125. OP, opisthotic and exoccipital project ventrally forming a flange over the foramen jugulare posterius

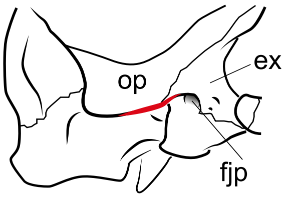

0. no (*Podocnemis expansa*)

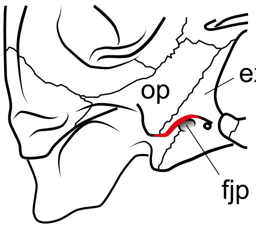

1. yes (*Bairdemys sanchezi*)

161. VT, Shape of the ventral keel on cervical 8

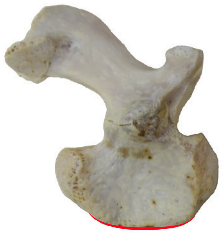

0. smooth straight or convex ventral edge (*Elseya dentata*)

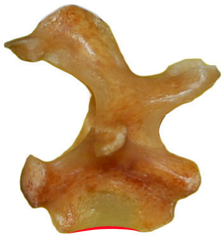

1. concave ventral edge (*Podocnemis sextuberculata*)

173. CAR, first pleural scute reaches nuchal

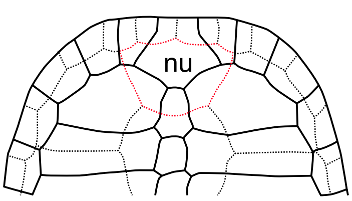

0. no (*Cearachelys placidoi*)

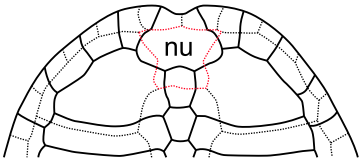

1. yes (*Chedighaii barberii*)

177. CAR, First neural sutured with nuchal

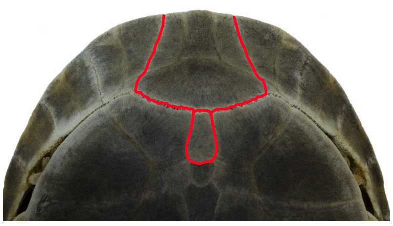

0. yes (*Phrynops geoffroanus*)

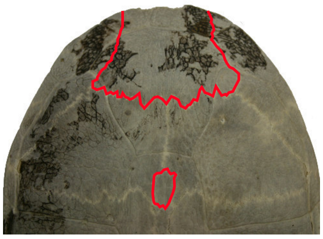

1. no (*Chelodina coillei*)

PLATE 2 - Characters 190, 210, 214, 215, 231, 235, 239

190. CAR, intumescence from axillary buttress to the rib area on costal 1

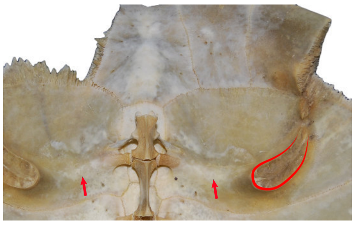

0. no (*Podocnemis unifilis*)

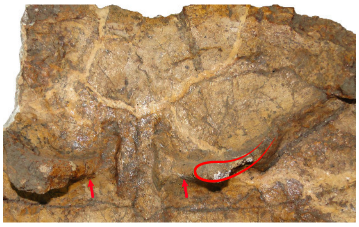

1. yes (*Bairdemys venezuelensis*)

210. CAR, Vertebral scute 5 reaches last neural

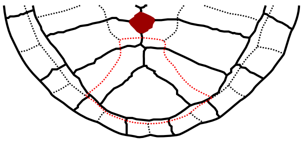

0. no (*Pelusios castanoides*)

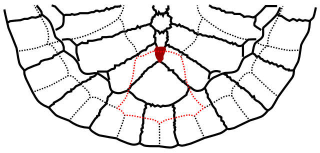

1. yes (*Araripemys barretoi*)

214. CAR, pleural scute 4 touches suprapygal

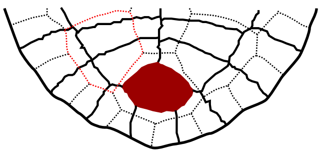

0. no (*Phrynops geoffroanus*)

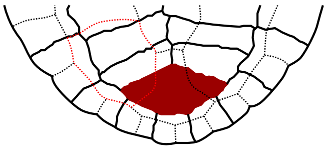

1. yes (*Platemys platicephala*)

215. Position of the posterior edge of the plastron related to carapace in ventral view

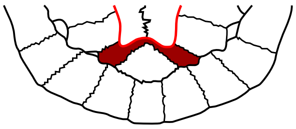

0. above C8 or more cranial, peripherals entirely visible (*Araripemys barretoi*)

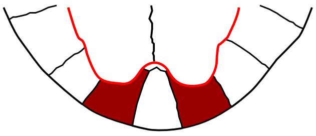

1. above peripherals 11 (*Podocnemis unifilis*)

231. PLA, extragulars size

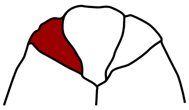

0. large, almost the same size as gular (*Chelus fimbriatus*)

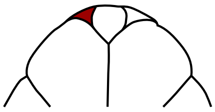

1. smaller than gular (*Pelomedusa subrufa*)

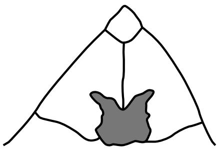

2. extragulars absent (*Araripemys barretoi*)

239. PLA, pubic scar shape

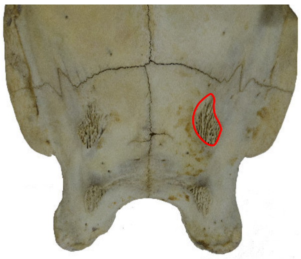

0. wider as in *Phrynops hilarii* (largest width/length >0.40)

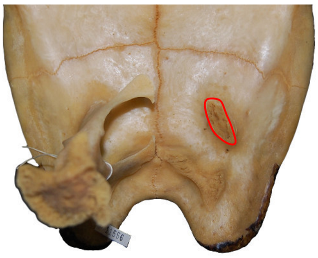

1. narrower as in *Podocnemis unifilis* (largest width/length <0.40)

235. PLA, humeral scale

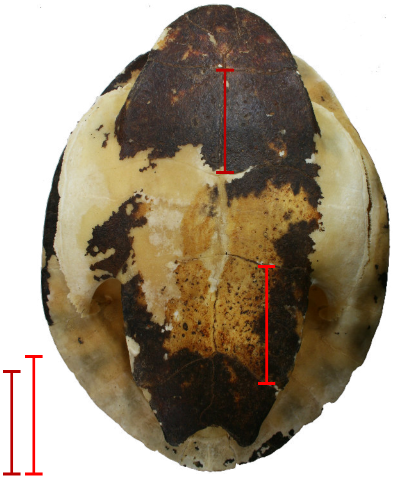

0. longest length humeral = > longest length pectoral (*Elseya dentata*)

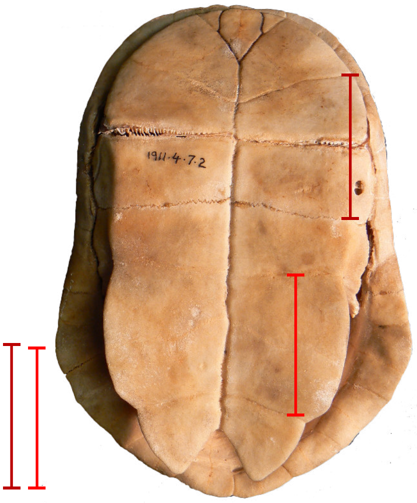

1. longest length humeral < longest length pectoral (*Pelusios castanoides*)

PLATE 3 - Characters 237, 241, 244

237. PLA, strong constriction on femoral-anal scute on xiphiplastra

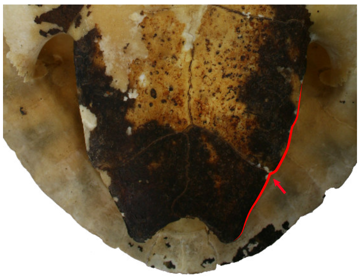

0. no, smooth edge (*Elseya dentata*)

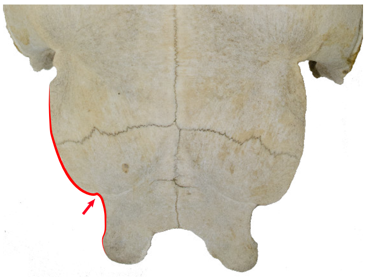

1. yes (*Phrynops hilarii*)

241. PLA, ischial scar position related to the lateral edges of xiphiplastron

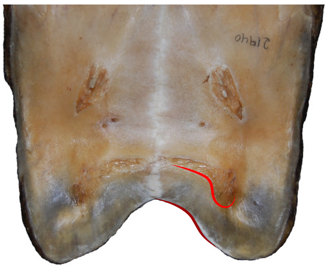

0. far from the caudal and lateral edges of xiphiplastron (*Peltoceph-alus dumerilianus*)

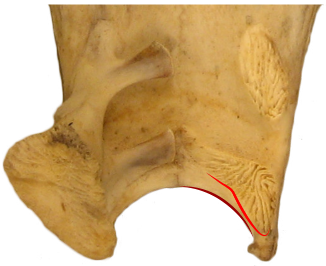

1. closer to the caudal and lateral edges of xiphiplastron (*Chelus fimbriatus*)

244. PLA, gular position

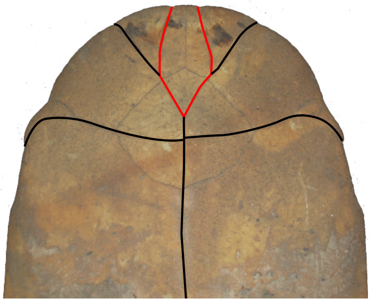

0. gular reaches cranial margin of plastron (*Podocnemis expansa*)

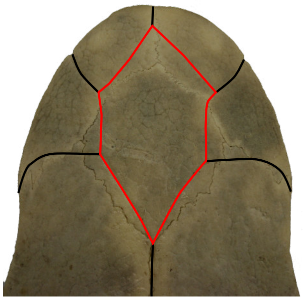

1. gular retracted from cranial margin of plastron (*Chelodina colliei*)
